# Integrating microarray analysis and the soybean genome to understand the soybeans iron deficiency response

**DOI:** 10.1186/1471-2164-10-376

**Published:** 2009-08-13

**Authors:** Jamie A O'Rourke, Rex T Nelson, David Grant, Jeremy Schmutz, Jane Grimwood, Steven Cannon, Carroll P Vance, Michelle A Graham, Randy C Shoemaker

**Affiliations:** 1 Department of Genetics, Developmental and Cellular Biology, Iowa State University, Ames, Iowa 50011 USA; 2 USDA-ARS, Corn Insect and Crop Genetics Research Unit, Iowa State University, Ames, Iowa 50011 USA; 3 Department of Agronomy, Iowa State University, Ames, Iowa 50011 USA; 4 Joint Genome Institute – Stanford Human Genome Center, Department of Genetics, Stanford University School of Medicine, Palo Alto, CA 94304 USA; 5 USDA-ARS, Plant Science Research Unit, University of Minnesota, St. Paul, MN 55108 USA

## Abstract

**Background:**

Soybeans grown in the upper Midwestern United States often suffer from iron deficiency chlorosis, which results in yield loss at the end of the season. To better understand the effect of iron availability on soybean yield, we identified genes in two near isogenic lines with changes in expression patterns when plants were grown in iron sufficient and iron deficient conditions.

**Results:**

Transcriptional profiles of soybean (*Glycine max*, L. Merr) near isogenic lines Clark (PI548553, iron efficient) and IsoClark (PI547430, iron inefficient) grown under Fe-sufficient and Fe-limited conditions were analyzed and compared using the Affymetrix^® ^GeneChip^® ^Soybean Genome Array. There were 835 candidate genes in the Clark (PI548553) genotype and 200 candidate genes in the IsoClark (PI547430) genotype putatively involved in soybean's iron stress response. Of these candidate genes, fifty-eight genes in the Clark genotype were identified with a genetic location within known iron efficiency QTL and 21 in the IsoClark genotype. The arrays also identified 170 single feature polymorphisms (SFPs) specific to either Clark or IsoClark. A sliding window analysis of the microarray data and the 7X genome assembly coupled with an iterative model of the data showed the candidate genes are clustered in the genome. An analysis of 5' untranslated regions in the promoter of candidate genes identified 11 conserved motifs in 248 differentially expressed genes, all from the Clark genotype, representing 129 clusters identified earlier, confirming the cluster analysis results.

**Conclusion:**

These analyses have identified the first genes with expression patterns that are affected by iron stress and are located within QTL specific to iron deficiency stress. The genetic location and promoter motif analysis results support the hypothesis that the differentially expressed genes are co-regulated. The combined results of all analyses lead us to postulate iron inefficiency in soybean is a result of a mutation in a transcription factor(s), which controls the expression of genes required in inducing an iron stress response.

## Background

Iron is a critical micronutrient for both plant and animal nutrition, serving as a required co-factor for a variety of cellular processes. Iron deficiency anemia is one of the leading human nutritional disorders worldwide, affecting 43% of the population of developing countries [1]For most of the world's population, legumes are a major source of dietary iron [1,2]. Though iron composes 5% of the earth's crust [3] it is largely unavailable to plants, particularly in calcareous soils with a pH greater than 7.5. Calcareous soils are especially prevalent in the upper Midwest of the US [4,5] and have been implicated in iron deficiency in soybeans. Iron deficiency chlorosis (IDC) in soybeans is characterized by interveinal chlorosis of the developing trifoliates [6]contributing to yield loss directly proportional to the severity of the chlorosis [6].

Plants have evolved two systems to uptake iron from the soil. These systems are termed strategy I and II [7,8]. Soybeans and other dicots utilize strategy I, in which the rhizosphere is acidified by the release of protons to produce a favorable environment for the release of iron from chelating agents in the soil. A membrane bound reductase reduces iron to the usable Fe^+2 ^form. The iron is then transported across the plasma membrane by a specific transporter for distribution and use within the plant. The reduction of the iron from Fe^3+ ^to Fe^2+ ^has been shown to be the rate-limiting step in IDC [9]. Graminaceous monocots utilize strategy II, whereby the roots release chelators called phytosiderophores to bind Fe^+3 ^ions. Once bound, the entire complex is transported into the root where it is uncoupled. The Fe^+3 ^ion is reduced to Fe^+2 ^and the phytosiderophores are re-released into the soil.

The quantitative nature of IDC makes field studies problematic. Previous studies have identified multiple Quantitative Trait Locus (QTL) associated with IDC [4,10]. Many of the same QTL have been identified in both field and greenhouse studies, where plants are grown in a hydroponics system designed specifically to induce IDC[10]. Growing plants in a controlled greenhouse environment with regulated nutritional availability allows for reproducible induction of iron deficiency stress. In addition, the advent of microarray technology now allows for the identification of individual transcripts whose expression levels are affected by iron availability[11,12]. The availability of a whole-genome sequence assembly for the soybean genome has, for the first time, allowed us to genetically position differentially expressed genes induced by iron deficiency.

Genomic studies in many organisms have shown genes in close proximity to one another in the genome are often co-expressed. These co-expressed genes create clusters of expression neighborhoods [13] which are conserved by natural selection [14] A study in Arabidopsis showed clusters of up to 20 different genes were coordinately regulated, with a median cluster size of 100 kb [15]. In rice, approximately five percent of the genome has been associated with co-expressed gene clusters [16]. Initially co-expressed genes were thought to belong to similar biological pathways [15], but further studies have shown co-functionality to be a poor predictor of co-expression [17]. Instead, promoter analysis has found co-regulated genes are often regulated by common transcription factors [13,17,18] The co-expression of clustered genes may be partially regulated by the interaction of common promoter elements and transcription factors [18]. Co-regulated genes often have common transcription factors [17], so an increase in the number of transcription factor binding sites in promoter regions would increase the likelihood of the transcription factor binding and aiding in the expression of the gene cluster.

The objectives of our research are to identify a list of candidate genes with a potential involvement in soybean iron deficiency and to associate these genes with the genome sequence to determine any correlation with previously identified QTL. We also wanted to determine whether the changes in candidate gene expression were due to structural or sequence differences in the candidate genes. The results from these analyses confirmed the co-expressed genes were co-localized and possibly coordinately regulated.

## Results

### Candidate Gene Identification and GO analysis

RNA from the second trifoliate of both iron efficient Clark and iron inefficient IsoClark grown under iron limiting conditions (50 uM Fe(NO_3_)_3_, iron inefficient plants show severe chlorotic symptoms) and iron sufficient conditions (100 uM Fe(NO_3_)_3_, no chlorotic symptoms in either genotype) were hybridized to the Affymetrix^® ^GeneChip^® ^Soybean Genome Array. Eight hundred and thirty-five transcripts were differentially expressed between Clark plants grown under iron sufficient and iron limiting conditions (Additional file 1). By comparison 200 transcripts differentially expressed between IsoClark plants grown under the same conditions (Additional file 2). Only 18 transcripts were common between the two lists (data not shown). Under iron deficient growth conditions, there were 179 genes differentially expressed between the two NILs (Additional file 3). However, an analysis of the data revealed only 21 transcripts met or exceed the two fold difference required to be considered differentially expressed between Clark and IsoClark genotypes grown under iron sufficient conditions (Table 1). This result confirms the NILs probably differ by only a limited number of genes.

**Table 1:** Differentially Expressed Genes between Clark and IsoClark Genotypes Grown Under Iron Sufficient Conditions

Affymetrix Probe ID	Fold Change	UniProt ID	Plant GOSlim AtHomolog	PlantGOSlim
GmaAffx.93650.1.S1_s_at	-12.383	Q6WE90	No Homolog	
Gma.12096.1.A1_at	-9.9	No UniProt	No Homolog	
Gma.18.1.S1_at	-9.416	Q39819	AT4G10250	response to stress
Gma.17141.1.S1_at	-8.776	Q9ZSA7	AT4G10490	other metabolic
Gma.14554.1.S1_at	-7.959	Q1SJ63		
GmaAffx.62046.1.S1_at	-5.922	Q5CAZ5	AT1G34210	developmental
Gma.2185.3.S1_at	-5.724	Q9FJL3	AT3G25230	response to stress
Gma.10282.1.A1_at	-5.591	Q1T3Y4	AT4G27670	response to stress
GmaAffx.90956.1.S1_s_at	-5.297	No UniProt	AT5G53740	biological process
Gma.11793.1.S1_at	-3.469	No UniProt	No Homolog	
GmaAffx.89665.1.A1_s_at	-3.365	Q9AY32	No Homolog	
Gma.12660.1.A1_at	-3.173	Q8H2B1	AT1G56300	protein metabolism
Gma.10282.2.S1_at	-2.899	Q1T3Y4	AT4G27670	response to stress
GmaAffx.93424.1.S1_x_at	-2.884	Q9S7H2	AT5G20620	protein metabolism
GmaAffx.56241.2.S1_at	-2.739	Q9SCW4	AT5G62020	transcription
Gma.8636.1.S1_at	-2.459	O80982	AT2G26150	transcription
GmaAffx.72322.1.S1_at	-2.32	Q7F1F2	AT5G48570	protein metabolism
Gma.1727.1.S1_at	-2.297	Q1SVQ0	AT2G39730	response to abiotic or biotic stimulus
GmaAffx.5924.1.S1_at	-2.295	Q8L7T2	AT1G52560	response to stress
GmaAffx.74022.1.S1_at	-2.267	Q1RY14	AT4G28480	protein metabolism
GmaAffx.93424.1.S1_s_at	-2.261	Q9S7H2	AT5G20620	protein metabolism

Fold Change between Iron Sufficient and Iron Deficient: Change in expression levels between Clark and IsoClark plants grown in iron sufficient conditions. A negative fold change is a down regulation of gene expression in the IsoClark genotype in comparison to the Clark genotype.

UniProt ID: Identifier for the UniProt sequence most similar to the consensus sequence used to create the Affymetrix probe set

Plant GO Slim At Homolog: The Arabidopsis homolog of the soybean consensus sequence represented by the Affymetrix probe set.

Plant GO Slim: The GO Slim annotation for the identified Arabidopsis homolog.

GO slim categories that were over represented in our lists of differentially expressed genes were identified for both the Clark and IsoClark comparisons. Transcripts with GO slim classifications that are over represented on our list of differentially expressed genes should be representative of the processes and pathways being affected in both the iron efficient and iron inefficient plants. The Clark genotype had 488 out of 835 unique transcripts with GO slim IDs. Of the corresponding GO slim IDs, 24 were over represented in our list of differentially expressed genes (Table 2) in comparison with the entire chip. The over represented GO slim categories could be further divided into 14 biological process IDs, 9 molecular function IDs, and 1 cellular component processes (Table 2). Of the 200 differentially expressed genes in the IsoClark genotype, 49 had corresponding Arabidopsis GO slim IDs. Of these, 21 genes had GO annotations that were over represented. These GO categories fell into two molecular function categories and three biological process categories (Table 3).

**Table 2:** GO Slim Terms Over Represented in Candidate Genes from Comparison Between Clark Plants Grown in Iron Sufficient and Iron Deficient Hydroponics Solutions

GO Slim ID	GO Term Description	Number of Genes with GO ID	Bonferroni Corrected P-Value
GO:0000004 BP	Unknown Function	128	0
GO:0006270 BP	DNA Replication Initiation	10	0
GO:0009611 BP	Response to Wounding	36	0
GO:0009695 BP	Jasmonic Acid Biosynthesis	24	0
GO:0006826 BP	Iron Ion Transport	9	1.2E-07
GO:0006879 BP	Iron Ion Homeostasis	10	3.6E-07
GO:0010039 BP	Response to Iron Ion	9	5.28E-06
GO:0009617 BP	Response to Bacterium	8	0.000018
GO:0006275 BP	Regulation of DNA Replication	4	0.00303
GO:0006972 BP	Hyperosmotic Response	4	0.00303
GO:0030397 BP	Membrane Disassembly	8	0.004381
GO:0008299 BP	Isoprenoid Biosynthesis	10	0.006706
GO:0009408 BP	Response to Heat	20	0.014334
GO:0019373 BP	Epoxygenase P450	5	0.045066
GO:0008199 MF	Ferric Iron Binding	9	0
GO:0008094 MF	DNA-dependent ATPase Activity	10	1.29E-06
GO:0016165 MF	Lipoxygenase Activity	12	0.000128
GO:0047763 MF	Cafeate O-Methyltransferase	8	0.001014
GO:0030337 MF	DNA Polymerase Processivity Factor	4	0.001426
GO:0005544 MF	Calcium Dependent Lipid Binding	8	0.001965
GO:0009978 MF	Allene Oxide Synthase	5	0.017949
GO:0046423 MF	Allene Oxide Cyclase	4	0.020157
GO:0008815 MF	Citrate (Pro-3S) Lyase	5	0.029946
GO:0009346 CC	Citrate Lyase	5	0.002807

GO Slim ID's statistically over or under represented in the list of differentially expressed genes comparing Clark plants grown in iron sufficient and iron limiting hydroponics conditions in comparison to their presence on the Affymetrix^® ^GeneChip^®^. The GO Slim Column provides the GO Slim ID number and corresponding category. GO Slim categories are Biological Process (BP), Molecular Function (MF), and Cellular Component (CC). The GO Term Description column contains the best description for the GO Slim ID. The number of genes with GO ID column is the number of genes differentially expressed between Clark plants grown in iron sufficient and iron deficient hydroponics solutions with the corresponding GO Slim ID. The Bonferroni Corrected P-value is the statistical significance of the GO Slim ID. Only GO Slim IDs with P-values equal to or less than 0.5 were considered statistically over or under represented in the list of differentially expressed genes in comparison to the presence of the GO Slim ID on the GeneChip^®^.

**Table 3:** GO Slim Terms Over or Under Represented in Candidate Genes from Comparison Between IsoClark Plants Grown in Iron Sufficient and Iron Deficient Hydroponics Solutions

GO Slim	GO Term Description	Number of Genes with GO ID	Bonferroni Corrected P Value
GO:0000004 BP	Unknown Function	49	0
GO:0006809 BP	Nitric Oxide Biosynthesis	4	0.0006102
GO:0010025 BP	Wax Biosynthesis	5	0.00736524
GO:0019953 BP	Sexual Reproduction	5	0.01223184
GO:0008940 MF	Nitrate Reductase	4	0.00006192
GO:0008382 MF	Iron Superoxide Dismutase	3	0.01245882

GO Slim ID's statistically over or under represented in the list of differentially expressed genes comparing Clark plants grown in iron sufficient and iron limiting hydroponics conditions in comparison to their presence on the Affymetrix^® ^GeneChip^®^. The GO Slim Column provides the GO Slim ID number and corresponding category. GO Slim categories are Biological Process (BP), Molecular Function (MF), and Cellular Component (CC). The GO Term Description column contains the best description for the GO Slim ID. The number of genes with GO ID column is the number of genes differentially expressed between IsoClark plants grown in iron sufficient and iron deficient hydroponics solutions with the corresponding GO Slim ID. The Bonferroni Corrected P-value is the statistical significance of the GO Slim ID. Only GO Slim IDs with P-values equal to or less than 0.5 were considered statistically over or under represented in the list of differentially expressed genes in comparison to the presence of the GO Slim ID on the GeneChip^®^.

Examining the GO terms associated with the candidate genes provides further insight into the disparity of the number of differentially expressed genes between genotypes. The IsoClark (inefficient) genotype does not appear to induce the full complement of genes induced in Clark in response to the iron deprivation stress. The most prevalent GO term in all three classifications for both genotypes was 'unknown function' (Tables 2 and 3). However, the Clark (efficient) genotype also had a high proportion of GO terms (and thus, transcripts) specifically related to iron availability and usage, (ferric iron binding (GO:0008199), iron ion transport (GO:0006826), and iron ion homeostasis (GO:0006879)) that were over-represented on our lists of candidate genes responding to iron stress. In addition, Clark genes encoded a number of GO terms not specifically related to iron, but which are associated with a more general stress response (GO:0009611 – response to wounding, GO:00099 jasmonic acid biosynthesis, and GO:0009408 – response to heat).

### Real Time PCR Confirmation

The differential expression observed through sqRT-PCR analysis mirrored, in direction, the expression differences observed in the microarray study (Table 4). The difference in expression levels seen in between the sqRT-PCR and the microarray experiment is most likely due to cross hybridization. Multiple members of the same gene family may hybridize to the same spot on the microarray, while the sqRT-PCR experiment is designed to amplify only single members of the gene family. The sqRT-PCR experiments confirmed the iron deficient plants had lower expression levels of the transcripts than the iron sufficient plants, replicating the results seen in the microarray data.

**Table 4:** Semi Quantitative Real Time PCR Results

Affymetrix Probe Set	Annotation	Conditions of Differential Expression	Differential Expression in Microarray	Diff Express in sqRT-PCR
Gma.13296.3.S1_at	Lipid Transfer	CSSvCSD	190.34	2.62
Gma.17724.3.S1_at	GDSL motif	CSSvCSD	6.26	8.46
Gma.17825.1.A1_at	GDSL motif	CSSvCSD	4.69	10.26
GmaAffx.89896.1.S1_at	Heat Shock Protein	CSSvCSD	23.45	2.04
GmaAffx.93268.1.S1_at	Heat Shock Protein	CSSvCSD	19.91	1.36
GmaAffx.51733.1.A1_at	Ribonuclease T2	CSSvCSD	-13.35	-2.98
GmaAffx.88242.1.S1_at	SnRNP protein	CSSvCSD	3.58	11.18
Gma.16500.1.S1_at	Lipoxygenase	CSSvCSD	11.36	16.9
Gma.3705.1.S1_at	Nitrate Reductase	CSDvICSD	-3.53	-1.101
Gma.9609.1.S1_at	Reductase	CSDvICSD	2.26	2.23
GmaAffx.36066.1.S1_at	Replication Factor	CSDvICSD	2.63	3.58

Details of sqRT-PCR experiments. Affymetrix Probe set denotes the differentially expressed sequence for which primers were designed. Conditions of Differential Expression represent the genotype and growth conditions in the comparison where the sequence was differentially expressed (CSS – Clark Shoots Iron Suficient, CSD – Clark Shoots Iron Deficient, ICSD – IsoClark Shoots Iron Deficient). Differential Expression in Microarray, the change in gene expression for the sequence from the microarray (in fold change). Differential Expression in sqRT-PCR, the change in gene expression for the sequence seen in the sqRT-PCR experiment (in fold change).

### Positioning Candidate Genes on the 7X Genome Assembly

**Figure 1:**
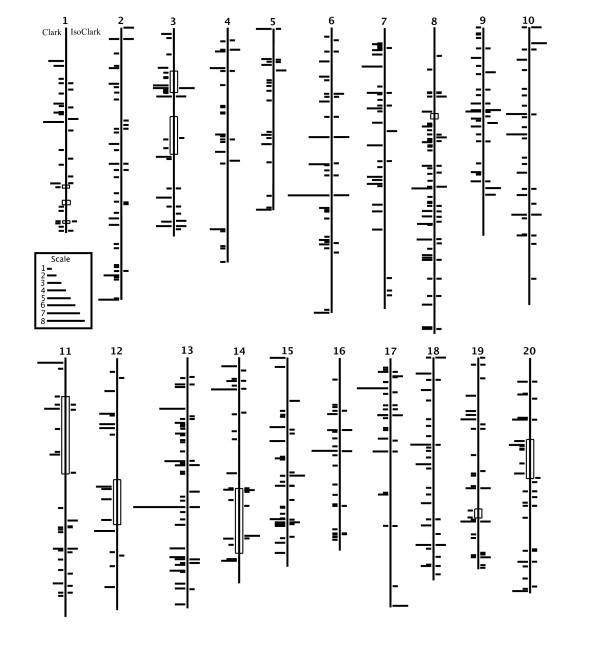
**Differentially Expressed Genes on Soybean Chromosomes**. Genes identified as differentially expressed in the microarray experiment have been aligned on the 7X build of the soybean genome, assembled into chromosomes. Each horizontal line represents one gene; longer lines represent multiple genes. Lines to the left of the chromosome are genes in Clark; lines to the right of the chromosome are genes in IsoClark. Open boxes on the chromosomes represent previously identified iron QTL regions. Clusters of differentially expressed genes are apparent throughout the genome.

Sequencing of the soybean genome by the Department of Energy, Joint Genome Institute currently has produced 7X sequence coverage of the genome http://www.phytozome.net, which has been assembled by USDA-ARS researchers into twenty draft pseudo chromosomes based on marker homology, allowing us to place our candidate genes on specific chromosomes (Figure 1).

The sequences of transcripts identified as differentially expressed by microarray analysis (see above) were obtained from the Affymetrix^® ^website http://www.affymetrix.com. These sequences were then queried against the 7X soybean genome using BLASTN [19] and an e-value cutoff of 10E^-50 ^to ensure a high sequence similarity between the aligned sequences. The same parameters were used to compare the sequences of SFPs to the 7X genomic sequence assembly. Markers used in previous iron QTL studies were also identified on the pseudo chromosomes to delineate known iron QTL regions (Figure 1). The iron efficiency QTL were scaled to the 7X build and used to determine if any of the candidate genes from the microarray experiment were encoded within the iron QTL regions. Fifty-eight genes in the Clark genotype and twenty-one genes in the IsoClark genotype were located within previously identified iron QTL (Figure 1, Additional files 1 and 2).

Sequences of the delineated iron QTL regions were analyzed using FGENESH http://www.softberry.com using Arabidopsis as the training model to identify gene structure predictions. The identified gene sequences were mined from the genome sequence and compared to known transcription factors in Arabidopsis http://datf.cbi.pku.edu.cn/download.php[20] rice, soybean, barley, and medicago http://planttfdb.cbi.pku.edu.cn/index.php[21]. This comparison identified 780 predicted genes within the previously identified QTL regions that show high sequence similarity (10E^-50^) to known transcription factors. One of these, within a QTL region on chromosome 12, showed a 100% identity to the Arabidopsis FIT gene (AT2G28160).

### Cluster Analysis

The gene distribution simulation randomly placed genes across the assembled genome. A second analysis assumed 36% of the genome was heterochromatic as proposed by Singh and Hymowitz [22] and reflected in the distribution of predicted gene locations http://www.phytozome.net. This analysis further constrained the algorithm, reducing the probability of candidate genes exhibiting clustering within the genome. If the candidate genes were randomly located throughout the genome, we would expect the experimental results to closely mirror the simulated data study. However our results, for both clustering analyses, strongly indicate that the differentially expressed genes exhibit clustering of two or more genes within 1,000,000 bp, 100,000 bp, and 10,000 bp in the genome (Tables 5 and 6, Additional files 4 and 5).

**Table 5:** Clusters of Candidate Genes on 7X build of Soybean Genome from Clark genotype assuming 36% of the genome is heterochromatic.

1,000,000 base bins				
# of Genes	Stimulation Mean	Stimulation SD	Experimental Data	Sds from Sim Mean
0	523	7.94	569	5.79
1	221	11.92	188	-2.77
2	137	9.34	99	-4.07
3	56	6.37	44	-1.88
4	17	3.76	36	5.05
5	4	1.96	13	4.59
6	1	0.90	2	1.11
7	0	0.41	2	4.88
8	0	0.14	1	7.14

100,000 base bins				

# of Genes	Stimulation Mean	Stimulation SD	Experimental Data	Sds from Sim Mean

0	8885	6.47	8926	6.34
1	671	12.50	486	-14.8
2	42	6.05	98	9.26
3	2	1.29	22	15.5
4	0	0.22	2	9.09
5	0	0.03	1	33.33

100 base bins				

# of Genes	Stimulation Mean	Stimulation SD	Experimental Data	Sds from Sim Mean

0	95243	2.16	94678	-2.62
1	751	4.31	593	-36.66
2	5	2.16	71	30.55
3	0	0.10	7	70
4	0		0	
5	0		1	

Details of sqRT-PCR experiments. Affymetrix Probe set denotes the differentially expressed sequence for which primers were designed. Conditions of Differential Expression represent the genotype and growth conditions in the comparison where the sequence was differentially expressed (CSS – Clark Shoots Iron Suficient, CSD – Clark Shoots Iron Deficient, ICSD – IsoClark Shoots Iron Deficient). Differential Expression in Microarray, the change in gene expression for the sequence from the microarray (in fold change). Differential Expression in sqRT-PCR, the change in gene expression for the sequence seen in the sqRT-PCR experiment (in fold change).

**Table 6:** Clusters of Candidate Genes on 7X build of Soybean Genome from IsoClark Genotype assuming 36% of the genome is heterochromatic.

1,000,000 base bins				
# of Genes	Stimulation Mean	Stimulation SD	Experimental Data	SDs from Sim Mean
0	757	4.98	783	5.22
1	165	8.81	118	-5.33
2	33	4.40	38	1.14
3	4	2.02	8	1.98
4	0	0.66	7	10.60

100,000 base bins				
# of Genes	Stimulation Mean	Stimulation SD	Experimental Data	Sds from Sim Mean

0	9359	2.13	9325	-15.96
1	236	4.21	178	-13.78
2	5	2.07	28	11.11
3	0	0.25	4	16

100 base bins				
# of Genes	Stimulation Mean	Stimulation SD	Experimental Data	Sds from Sim Mean

1	95754	0.72	95128	-869.44
1	245	1.44	199	-31.94
2	1	0.72	22	29.17
3	0	0.03	1	33.33

Details of sqRT-PCR experiments. Affymetrix Probe set denotes the differentially expressed sequence for which primers were designed. Conditions of Differential Expression represent the genotype and growth conditions in the comparison where the sequence was differentially expressed (CSS – Clark Shoots Iron Suficient, CSD – Clark Shoots Iron Deficient, ICSD – IsoClark Shoots Iron Deficient). Differential Expression in Microarray, the change in gene expression for the sequence from the microarray (in fold change). Differential Expression in sqRT-PCR, the change in gene expression for the sequence seen in the sqRT-PCR experiment (in fold change).

The candidate genes do not show a high concordance with known iron QTL regions, but do serve to identify additional genomic regions of IDC transcriptional importance (Figure 1). The largest cluster contained eight candidate genes located within 1 MB on chromosome 6 (Figure 1). There was also a cluster of seven genes on chromosome 2 (Figure 1). Chromosome 2 contained six clusters of four or more genes within 1,000,000 bases, as did chromosome 13 (Figure 1). None of these clusters were located within known iron QTL. However, another cluster of seven genes falls on chromosome 12 (Figure 1), which has three known iron QTL, together spanning 24 cM of the linkage group. Chromosome 7 contained the most gene clusters, eight separate clusters, each with four candidate genes (Figure 1). Again, chromosome 7 has not been previously shown to contain regions of genetic importance to soybean IDC. A number of the gene clusters contain multiple copies of genes encoding proteins with similar functions. A cluster of six candidate genes on chromosome 9 all encode proline rich proteins while three of the genes in a cluster of 5 on chromosome 6 encode caffeic acid O-methyltransferases (Additional file 4). The close physical proximity of co-expressed genes of the same function provides further support for the clustering of co-expressed and co-functional gene clusters.

The identification of these gene clusters on chromosomes not previously known to be involved in soybean IDC opens new regions of genetic interest to investigate in future studies. The majority of the candidate genes identified were not within the iron QTL, nor were the largest clusters of differentially expressed genes. Genes affecting chlorosis and yield loss may not be confined to the previously identified QTL regions. However, the iron QTL must contain sequence of importance to IDC. It is likely a transcription factor(s) controlling the expression of genes required to induce an iron stress response is encoded within the QTL regions. We have identified 780 predicted transcription factors, including the soybean homolog to the Arabidopsis FIT gene, within the previously identified QTL. This, or another of these transcription factors may be responsible for inducing a cascade of gene expression changes due to limited iron conditions. The transcription factor/factors may also affect the expression of the canonical iron genes, such as IRT and FRO, none of which are encoded within the previously identified QTL, nor identified as differentially expressed in our microarray experiment.

### Motif Analysis

Previous research has demonstrated that genes clustered in close proximity in a genome may be coordinately regulated. To determine if clusters of IDC genes were coordinately regulated, we examined 500 bases from the 5' untranslated regions (putative promoters) of all differentially expressed IDC genes and used these as input into the MEME software program. As an internal control, sequences were not analyzed as members of clusters; rather, all sequences were analyzed as a single large group. If IDC genes were coordinately regulated, MEME could also be used to independently identify potential gene clusters. In total, the putative promoters of 835 iron deficiency induced genes from the Clark genotypic comparison and 200 genes from the IsoClark comparison were analyzed using MEME. There were no motifs found using MEME in the IsoClark (inefficient) promoter regions. All motifs identified by MEME were found in the promoter regions of genes differentially expressed due to iron deficiency in the Clark (efficient) genotype. Twenty-one motifs with E-values more significant than 10E^-6 ^were identified by MEME analysis. Following visual inspection, this number was reduced to 11. Motifs were eliminated if they contained repetitive sequence or had lower significance E-values. The 11 motifs were identified in 248 IDC genes, representing 129 of the clusters of two or more genes as mentioned above. One mechanism by which genes can be coordinately regulated is through the action of transcription factors that bind to the promoter to regulate gene expression. Therefore, the 11 motifs identified above were compared to known transcription factor binding sites in the TRANSFAC [23] database (Table 7). Three motifs showed significant sequence similarity (99% identity) to known transcription factor binding sites (Table 7). These three sites bind a helix-loop-helix transcription factor (bHLH), an elongation factor (EF2), and a Myb transcription factor. These binding sites were identified in the promoter regions of 42, 40, and 28 iron responsive genes respectively. Both helix loop helix and Myb transcription factors are known to be involved in regulating the iron stress response and general stress responses in other plant species.

**Table 7:** Motifs in Promoter Regions of Differentially Expressed Genes Identified by MEME and Their Similarity to Transcription Factor Binding Sites in the TRANSFAC Database

Identified Motif Sequence	# Seqs withMotif	Motif E-Value	TRANS FAC Hit ID	TRANS AC Binding Site Sequence	TRANS FAC annotation
CATCCAACGGC	29	1.2E-1	M00227	TCCAACGGC	Myb
CCCGCCACGCGCCAC	48	5.1E-26	M00187	GCCACGTGCC	Helix Loop Helix
TGGCGGGA	50	5.8E-13	M00024	TGGCGCGA	Elongation Factor 2
CCAAACCC	50	2.7E-5	No Hit		
CCACCACCACC	48	3.8E-16	No Hit		
ACACAACACAC	45	2.2E-10	No Hit		
AAAATAAAAATAAAA	9	2.27E-7	No Hit		
AATAAAAAAATAAAA	8	1.51E-7	No Hit		
AGCTAGCTAGC	6	1.47E-7	No Hit		
AGCGAGCGAGC	4	6.23E-8	No Hit		
AGCAAGCTAGC	3	2.47E-7	No Hit		

Identified Motif Sequence: The motif sequence identified by MEME analysis in the promoter region of differentially expressed genes from the Clark genotype.

# of Seqs with Motif: The number of differentially expressed genes whose promoter regions contain the identified motif.

Motif E-Value: E-value of the motif assigned by MEME.

TRANSFAC Hit ID: The TRANSFAC ID of the transcription factor binding site showing high homology to the identified motifs. Only three identified motifs showed homology to known transcription factor binding sites in the TRANSFAC database.

TRANSFAC Binding Site Sequence: The transcription factor binding site sequence. Mismatched bases are between the identified motif and the reported binding sites are underlined.

TRANSFAC Annotation: The type of transcription factor that binds to the identified TFBS according to TRANSFAC.

### SFP Analysis

The SFP analysis identified 170 single feature polymorphisms, seventy-two SFPs were unique to the Clark genotype and 98 unique to the IsoClark genotype. A number of the Affymetrix^® ^sequences found to contain an SFP perfectly matched more than one genomic location, giving a potential of 208 predicted genes with an SFP (Additional file 6). Only one of the 170 SFPs identified in this study (GmaAffx.41460.1.S1_at) was encoded within a gene identified as differentially expressed between IsoClark plants grown under iron sufficient and iron deficient conditions. The remaining SFPs were not in differentially expressed genes in either Clark or IsoClark genotypes. This suggests most of the SFPs are encoded in regulatory elements, which would not necessarily be differentially expressed. GO slim ID analysis, as previously described, was performed with the gene sequences containing SFPs. Of the genes containing SFPs, 20% had an unknown biological process annotation. The most prevalent group with known annotations was related to transcriptional regulation. Genes involved with electron transport, ATP binding, ligases, and transferases were also identified as over-represented by their GO IDs (data not shown).

## Discussion

### Microarray Analysis

Through a combination of a suite of analyses we have extended the fundamental understanding of the genetics underlying iron uptake and homeostasis in plants, but specifically soybean. Affymetrix gene chip analysis allowed us to identify candidate genes that are induced by iron deficiency in the leaf tissue of two NILs, complementing previous studies done in roots [11,12]. The Clark genotype analysis identified 835 differentially expressed genes when grown under iron sufficient and iron insufficient conditions while in IsoClark only 200 were identified (Additional files 1 and 2). These genes have been aligned with the genomic sequence to determine their location. A sliding window analysis determined the co-expressed genes are clustered in the genome, suggesting co-regulation. The SFP analyses determined the differentially expressed genes are not a result of structural differences in the genes between the two NILs, providing further support that the differentially expressed genes are being co-regulated. Finally, motif analysis identified 11 short conserved motifs in the promoter regions of the candidate genes, which are most likely transcription factor binding sites. The cumulative results of all analyses leads us to propose the differential iron response in the NILs is a result of a mutation in the iron inefficient NIL of a transcription factor, or factors, probably encoded within one or more of the previously identified QTL, that prevents the induction of the iron deficiency gene expression responses seen in the iron efficient NIL.

The candidate genes identified with the microarray experiment suggest the Clark genotype is capable of recognizing the iron deficiency and eliciting a change in transcription patterns as a response to the stress. We hypothesize that the iron deficient Clark plant compensates for the lack of iron availability by adjusting its physiological processes to conserve available iron. Alternatively, the IsoClark genotype does not appear to initiate an effective response to the iron deficient conditions. The lack of differentially expressed genes in the IsoClark genotype, when comparing iron sufficient and iron deficient conditions, implies the iron deficient IsoClark plant continues to function as if still in iron sufficient conditions. However, the lack of iron as a cofactor in many of the basic biological processes results in a multitude of biological pathway failures, resulting in chlorotic plants.

In Arabidopsis, iron deficiency stress causes an increase in the transcription of electron transport chain components. Specifically, cytochromes are upregulated [24]. Our experiment identified seventeen genes associated with cytochrome P450 in iron stressed Clark plants. All seventeen genes were down-regulated in iron stressed tissue compared to non stressed tissue, the opposite response as seen in Arabidopsis plants [24]. Thimm et al. proposed a correlation between iron deficiency stress and in induction of phosphoenolpyruvate carboxylase activity [24]. Four genes associated with phosphoenolpyruvate activity were identified as differentially expressed in the Clark genotype by microarray analysis. All four of these genes were down regulated in plants grown under iron stress rather than in iron sufficient conditions. Iron deficiency has also been shown to induce glycolytic activity [25]. Three enzymes involved in glycolysis; glyceraldehydes 3 phosphate dehydrogenase (G3PD), pyruvate kinase (PK), and fructose 6 phosphate kinase (F6PK) have been shown to be up-regulated in Arabidopsis [24] and cucumber [25] under iron deficiency stress. Microarray analysis comparing Clark plants grown in iron sufficient and iron stressed conditions only identified a single G3PD and a single F6PK, both of which were down-regulated in the iron stressed tissue compared to iron sufficient tissue. Seven genes associated with PK were identified in our microarray. Again, all seven were down regulated. The down regulation of the three main components of glycolysis suggests soybean, unlike Arabidopsis, does not increase non photosynthetic carbon fixation or phosphoenolpyruvate carboxylase activity under iron stressed conditions. The contrasting results support the hypothesis proposed by Zocchi et al. that soybeans do not follow canonical iron deficiency responses [26].

Soybean does follow some of the established responses to iron deficient stress conditions. It has been proposed that under iron deficient conditions citrate provides a carbon skeleton for chlorotic leaves to allow for sustained growth and respiration [27]. Clark iron deficient stressed plants show a down regulation of citrate lyase (GO: 0008815) in comparison to non-stressed plants. The reduced breakdown of citrate in iron stressed plants lends credence to this hypothesis. Additionally, iron deficient conditions cause decreased activity of lipoxygenases [24]. All thirteen lipoxygenases identified by microarray analysis in the Clark genotype showed decreased expression in the iron stressed tissue compared to the iron sufficient tissue.

The discrepancies between previously reported literature and the soybean iron deficiency response highlight the complexity of the iron stress response. However, it is important to remember that transcriptional regulation is only one form of regulation. Post-transcriptional modification may be an important component to understanding soybean's iron deficiency response, but that is beyond the scope of this investigation.

### GO Slim ID Analysis

The Clark (iron efficient) genotype had an over-representation of genes in GO slim categories specific to iron availability/usage and categories associated with a more general stress response (Table 2). This reinforces the hypothesis that Clark responds specifically to iron but also to a more general stress response. A similar pattern was observed in a cDNA microarray experiment [12]. Additionally, the Clark genotype showed a statistically significant number of GO slim IDs that were over-represented related to DNA replication and DNA binding activity. The increased expression levels of genes involved in these processes is probably a result of the DNA repair required to prevent lethal mutations from ROS [28], which are more prevalent under conditions of stress [28]. DNA binding activity suggests the activity of transcription factors, which lead to dramatic expression changes downstream. However, the down regulation of genes related to translation ie: GO0006412 (translation) and GO: 0006468 (protein amino acid phosphorylation) is indicative that the plant is not synthesizing proteins at a normal rate as it would under optimal growth conditions and is instead reducing the expression of genes involved in cellular processes not imperative to survival.

The IsoClark genotype had many fewer GO categories significantly over represented on our lists of candidate genes (Table 3) in comparison to Clark. Only two of the GO classifications were related to iron GO:0008940 (nitrate reductase activity) and GO:0008382 (iron superoxide dismutase activity). The remaining GO categories show little association to either a general or an iron specific stress response. It appears the IsoClark genotype is unable to recognize or respond to the iron stress. The IsoClark genotype had fewer genes differentially expressed due to iron deficiency and most of the genes that were differentially expressed are not associated with stress related pathways.

### Clusters of Co-Expressed Genes

Expression analysis has been used in some model organisms to identify differentially expressed genes that are clustered together within the genome [13-18,29,30]. These genomic neighborhoods are thought to be conserved by natural selection [14] but are not entirely explained by co-functionality [17]. The combined use of expression data with known QTL positions and expression clusters should further narrow the list of candidate genes to identify functionally important differences in the soybean genome affecting iron efficiency.

Co-expressed genes show a non random distribution throughout the genome [14,17]; where similarly expressed genes are located in clusters. Localized co-expression of genes has been reported in many different species including (but not limited to Arabidopsis [15,29,30], rice [16], human [13,31]and yeast [14]). Williams et al (2004) found genes located nearby in the genome and genes involved in the same pathways are more likely to be co-expressed. The incidence of co-expressed gene clusters has been widely studied [13-15,17,31]. One proposed explanation is that the co-expressed genes are regulated by a common transcription factor. Grouping these genes creates an increase in the abundance of binding sites specific to that transcription factor [31]. A related hypothesis suggests the co-expressed genes are regulated by similar promoter sequences, so a co-expression 'neighborhood' would increase the availability of these promoter sequences [18]. However, genomic studies have, as of yet, been unable to confirm either of the two hypotheses.

Cluster analysis, as first reported by Grant et al.(2000), was performed to determine if candidate genes identified by the microarray experiment were randomly distributed across the genome. Iterative simulations modeling our data showed our candidate genes were not distributed evenly throughout the genome. Using a sliding window of 1,000,000 bases, we identified more genes in smaller regions of the genome than expected by a random distribution of the differentially expressed genes with 3 – 8 candidate genes per 1,000,000 bases (Tables 5 and 6). The same patterning held true when the sliding window was reduced to 100,000 and 100 bases. The statistical significance, from comparing the experimental data to the simulated data, is found in the number of simulated standard deviations (SDs) the experimental data is from the simulated data (Simulation SD column in Tables 5 and 6). When comparing clusters of three or more genes in either Clark or IsoClark, there are only four instances (3 and 6 genes per cluster, Table 7; 2 and 3 genes per cluster, Table 8) where the difference between the experimental data and the simulation study is not statistically different.

In the Clark genotype, with a window of 1,000,000 bases, there were thirty-six clusters of four genes and thirteen clusters of five genes per window identified in the experimental data. There were only seventeen clusters of four genes and only four occurrence of five genes clustering together in the simulation study. The difference in SDs is 5.05 and 4.59 respectively, indicating a highly statistically significant difference. The million base window allowed larger gene clusters to be identified in the experimental data (two clusters of seven genes and a single cluster containing eight genes). No clusters of these sizes were identified in the simulation study, further supporting the clustering hypothesis. When the window size is decreased to 100,000 or 100 bases, three genes in a cluster become significantly over represented in the experimental data compared to the simulation study. The microarray experiment identified 22 clusters of three genes per 100,000 bases. No clusters of three or more genes were identified in the simulation study at either window size.

The IsoClark genotype identified fewer candidate genes in the microarray experiment, which reduces the number of gene clusters identified. However, even with a reduced number of candidates, IsoClark still exhibited clustering of co-expressed genes. With a window of 1,000,000 bases, there were eight clusters of three genes identified in the experimental data, but only four clusters are identified in the simulation study. There were seven clusters of four genes per million bases identified in the IsoClark simulation, but none in the simulated data. The retention of clusters, even among so few candidate genes, lends further support that the soybean genome has conserved genomic regions with co-expressed genes.

Individual gene clusters are interesting because so many of them contain multiple copies of similar genes (Additional files 4 and 5). For example, all six genes in the cluster on chromosome 9 encode proline rich proteins while three of the five genes in a cluster on chromosome 6 encode caffeic acid O-methyltransferase. The co-expression of these genes coupled with their close physical proximity lends further credence to the hypothesis that they are regulated by a common transcription factor.

### Single Feature Polymorphisms (SFPs)

Identifying candidate genes for a trait of interest is the most widely used method of analyzing the data provided by microarray experiments. However, mining the hybridization data to identify single feature polymorphisms (SFPs) provides a high throughput platform for detecting polymorphisms [32]. Single Nucleotide Polymorphisms (SNPs) are the most commonly recognized polymorphism, but identification is labor intensive and SNP coverage across the genome is fairly sparse [33]. It has been suggested that there is a greater probability of identifying a causal polymorphism for the trait of study using SFPs than traditional SNPs [33], perhaps due to better genic coverage.

To date, only 3 molecular markers (Satt 481, Satt114, and Satt239) segregate with the iron efficiency trait in soybean across multiple populations [34,35]. The 72 SFPs identified in the Clark genotype and the 98 SFPs identified in the IsoClark genotype relative to Williams 82 in this study have the potential to be developed into molecular markers specific to IDC. Initially, we hypothesized the SFPs would correlate with the differentially expressed genes. However, only one SFP (GmaAffx.41460.1.S1_at) was found in a gene differentially expressed in IsoClark leaf tissue. In Arabidopsis this gene is essential for NADH mediated reduction of the plastiquinone pool in respiratory electron transport and is up-regulated under mild heat stress [36]. It is logical that this gene might be differentially expressed in the iron inefficient plant as photosynthesis slows due to a lack of iron serving as electron transporters. The remaining169 SFPs were not differentially expressed due to iron stress. The majority of the sequences identified containing an SFP have an unknown function and the largest class of annotated SFPs is transcription factors (Additional file 6). These SFP polymorphisms may alter transcription and/or translation rates of key genes and proteins, or serving some other regulatory function in soybean iron homeostasis.

### Promoter Motifs

Analysis of the 500 bp upstream of the start codon for the predicted genes in soybean http://www.phytozome.net that coordinate with the differentially expressed candidate genes identified 11 conserved motifs (Table 7). These small motifs were notable for both their highly conserved sequences and conserved positions. A comparison of these motif sequences to the TRANSFAC database identified three of the 11 motifs as transcription factor binding sites (TFBS). It is likely that the remaining eight motifs represent previously uncharacterized transcription factor binding sites. One of the three motifs that showed high similarity to a TRANSFAC TFBS was a Myb TFBS. Myb transcription factors have been implicated in inducing the stress response in plants in response to various abiotic stresses including phosphate stress [37] and asian soybean rust [38]. The identification of the Myb TFBS in the promoter region of candidate genes from the Clark genotype supports the idea that Clark is able to induce both an iron specific stress response and a more generalized stress response.

The identification of a basic helix loop helix (bHLH) TFBS motif in the promoter region of candidate genes from the Clark genotype may be indicative of the iron specific stress response induced in Clark under iron deficient conditions. In Arabidopsis, bHLH proteins have been identified as essential components in mediating iron uptake under iron stress conditions. Specifically, AtbHLH38 and AtbHLH39 both form heterodimers with AtbHLH29 to regulate iron uptake gene expression under iron deficient conditions in Arabidopsis [39]. AtbHLH29 encodes a transcription factor known as FIT (FER like iron deficiency induced transcription factor [40]), which dimerizes with either AtbHLH38 or 39 to induce FRO2 and IRT1 gene expression [39]. Though the soybean FIT homolog was not identified as differentially expressed due to iron deficiency, it was one of the 780 transcription factors predicted to be encoded within previously identified QTLs. The importance of bHLH transcription factors in regulating iron uptake gene expression makes the identification of a bHLH TFBS in the promoter region of iron deficiency induced genes particularly exciting. This is the first evidence that iron uptake gene expression may be similarly regulated in Arabidopsis and soybean.

### Iron QTL and the Soybean Genome

QTL mapping and marker assisted selection have been utilized by plant breeders for decades in the pursuit of crop improvement. This approach has been especially important for quantitative traits such as iron deficiency chlorosis [4,34,35,41]. Only in recent years have scientists been able to utilize microarray technology to examine gene expression on a global scale to identify candidate genes for their trait of interest. The development of the Affymetrix^® ^GeneChip^® ^Soybean genome array [42], representing approximately 75% of the predicted genes in soybean (data not shown), means repeatable precision, providing more confidence to the microarray experiments than cDNA arrays.

The availability of the whole-genome soybean sequence has provided the ability to visualize the placement of candidate gene sequences within the genome. This view will allow further insight into soybeans' response to iron deficiency stress. Nineteen QTL regions have been previously identified for iron deficiency chlorosis, both in field and hydroponic studies [4,10,41]. These regions represent approximately 182 cM of genetic information. Our initial hypothesis was that the majority of the genes identified in the microarray experiment would map within known iron QTL regions. However, only 58 of the 835 (7%) candidates in the Clark genotype and 21 of the 200 (10%) in the IsoClark genotype mapped within known QTL regions (Figure 1, Additional files 1 and 2). Thus, the majority of the candidate genes identified in this study lie outside of regions identified as iron QTL. However, given the evidence of coordinate gene expression, gene clustering and conserved promoter motifs in our data, we have revised our previous hypothesis. We now propose the previously identified QTL regions likely correspond to transcription factors that regulate gene expression during iron stress. While microarray experiments would identify IDC regulated genes whose expression changes in response to a transcription factor, they may not identify the transcription factor itself. In contrast, QTL mapping would identify a mutation in a transcription factor, which is at the top of the signaling pathway. The mutation would effect either the expression of the transcription factor or its ability to bind to target promoters. This hypothesis is supported by our data. The clustering of co-expressed genes suggests they are being coordinately regulated. This is supported by the conserved motifs identified in the promoter regions of candidate genes. Most often, motifs are conserved throughout a previously identified cluster of genes in Clark. It is unlikely these motifs are missing or are altered in the promoter regions of the IsoClark genome. More likely, IsoClark may have a mutation in the transcription factor that controls the expression of these genes. Only by combining QTL analyses, microarray analyses of NILs, and the genome sequence could this conclusion be reached.

## Conclusion

The use of near isogenic soybean lines, microarray analysis, SFP identification, and the sequence of the soybean genome has allowed us to identify individual genes lying within known iron efficiency QTL whose expression levels are affected by iron availability. We have also identified 11 conserved motifs in the promoter sequence of genes differentially expressed due to iron deficiency stress. The 58 differentially expressed genes identified in Clark and 21 in IsoClark, located within known QTL regions, are the first genes identified by microarray analysis within QTL regions specific to iron deficiency stress. The conserved motifs throughout the promoter regions of the differentially expressed genes in the Clark genotype provide compelling evidence that the differential iron response is likely due to the differential expression or binding of a transcription factor. Co-expressed genes clustered either by physical proximity (Tables 5 and 6) or through shared promoter motifs (Table 7) provide new regions of genetic interest in the study of iron deficiency chlorosis in soybean. Additionally, both types of clustering suggest the control of soybeans' iron deficiency response is regulated by the differential expression of a transcription factor or a mutation within the transcription factor, which affects its ability to bind to target promoter regions. This implies the eight transcription factors differentially expressed in Clark under iron deficiency stress which are located within known iron QTL regions are likely candidate genes for the QTL. An analysis of the 780 transcription factors predicted within the IDC QTL regions, specifically the FIT homolog, additional 52 bHLH transcription factors, and the other 50 genes in Clark and 21 genes in IsoClark that map within the QTL regions may further elucidate the response induced in soybean due to iron deficiency stress. Additionally, the conserved motifs identified by MEME in the promoter regions of iron deficiency induced genes can be used to mine the soybean genome for additional genes potentially affected by IDC, but which are not represented on the soybean Affymetrix^® ^GeneChip^®^.

## Materials and methods

### Plant Growth and RNA Extractions

NILs developed for their characteristic response to limited iron conditions, were developed by the USDA-ARS [43]. The iron efficient PI548533 [18] was crossed with iron inefficient T203 (PI54619). Five repeated backcrosses to Clark yielded the iron inefficient line PI547430 (IsoClark). Both the iron efficient Clark and the iron inefficient IsoClark were germinated in sterile vermiculite and transferred to a DTPA buffered nutrient hydroponics system 7 days after planting. Each 10L hydroponic unit contained 2 mM MgSO_4 _*7H_2_O, 3 mM Mg(NO_3_)_2 _*6H_2_O, 2.5 mM KNO_3_, 1 mM CaCl_2 _*2H_2_O, 4.0 mM Ca(NO_3_)_2 _*4H_2_O, 0.020 mM KH_2_PO_4_, 542.5 μM KOH, 217 μM DTPA, 1.52 μM MnCl_2 _*4H_2_O, 4.6 μM ZnSO_4 _*7H_2_O, 2 μM CuSO_4 _*5H_2_O, 0.20 μM NaMoO_4 _*2H_2_0, 1 μM CoSO_4 _*7H_2_O, 1 μM NiSO_4 _*6H_2_O, 10 μM H_3_BO_3_, and 20 mM HCO_3_. A pH of 7.8 was maintained by the aeration of a 3% CO_2_: air mixture. A supplemental nutrient solution containing 16 mM potassium phosphate, 0.287 mM boric acid and 355 mM ammonium nitrate was added daily to maintain proper plant nutrition. Both iron efficient and iron inefficient plants were grown in iron sufficient (100 uM Fe(NO_3_)_3_) and iron limiting (50 uM Fe(NO_3_)_3_) hydroponic conditions. Leaf tissue from the 2^nd ^trifoliate was collected 21 days after planting, or after 14 days in the hydroponics system. Tissue was flash frozen in liquid nitrogen and stored at -80°C until RNA could be extracted. Three independent biological replicates were used as the experimental tissue. RNA extractions were performed using the Qiagen RNeasy Plant Mini Kit (catalog # 74904). RNA samples were submitted to the Iowa State University GeneChip^® ^facility to be hybridized and scanned using the Soybean Affymetrix^® ^GeneChip^®^. Chip data has been uploaded to Gene Expression Omnibus as accession number GSE10730. Hybridization data was visualized using Bioconductor to ensure all hybridizations had normal distributions. The data was then loaded into the Gene Traffic Microarray Analysis program where it was normalized using the invariant set command, using the Clark 100 uM Fe as the control group, and a model based expression index (MBEI) [44] analysis was performed on perfect match probes only. Hybridization statistics were used to determine a two-fold change in expression, consistent across all replicates, reflected a statistically significant (p values and standard errors generated by analysis not shown) difference in expression between genotype and iron concentrations. An analysis of Clark plants grown in iron sufficient and iron deficient conditions showed 835 transcripts differentially expressed at two-fold or greater (Supplemental Table 1). IsoClark plants grown in identical conditions showed 200 transcripts that met the criteria for differential expression (Supplemental Table 2).

### Candidate Gene Annotation

The candidate genes were queried against the SoyBase Affymetrix^® ^GeneChip^® ^Soybean Genome Array Annotation page, publicly available at http://www.soybase.org/AffyChip/. Here, researchers with the USDA-ARS have used BLASTX and TBLASTX [19] to compare the sequences from which all Affymetrix probes were derived to the UniProt database and the Arabidopsis genome gene calls (TAIR7, http://www.arabidopsis.org/). The top three UniProt BLAST hits and the Arabidopsis best hit GO annotation is reported for each Affymetrix probe set. To assign a putative function and classification to the differentially expressed genes (Table 1, Additional files 1, 2, and 3) the three UniProt annotations were compared. If all three were identical that annotation was assigned to the gene. If the top three BLAST hits were not in concordance, that sequence was re-examined to determine if one of the annotations was more likely correct than the others. If no annotation could be confidently identified by BLAST analysis with UniProt, the differentially expressed gene was annotated as an unknown. If the gene sequence for the Affymetrix^® ^probe showed no sequence homology to any of the proteins in the UniProt database, the sequence was annotated as No UniProt Hit.

### GO Slim Term Analysis

For expressed genes with homology greater than 10e^-6 ^to an Arabidopsis gene, custom perl scripts were written to parse and tally each transcript GO slim ID for biological process, molecular function, and cellular process. The same scripts were used to tally GO slim IDs for the entire chip. Differences between the expressed genes and the entire chip were compared using a Fisher exact test [45]. This test was performed to identify the GO slim terms within each of the three GO slim classifications that were over-or under-represented in the lists of differentially expressed genes in relation to their presence on the soybean Affymetrix^® ^chip. A Bonferroni correction [46], using the number of identifiers present on the Affymetrix^® ^chip, was applied to the two-tailed probability value (p-value) of each GO slim identifier. GO slim identifications with a p-value of less than or equal to 0.05 after the Bonferroni correction were considered statistically over-or under-represented in our list of differentially expressed genes (Tables 2 and 3). This correction is likely to underestimate the number of categories of genes either over-or under-represented on the lists of differentially expressed genes in comparison to their prevalence on the Affymetrix^® ^chip.

### Real Time PCR Confirmation

The differential expression observed in the microarray experiment to identify candidate genes was confirmed using semi quantitative Real Time Reverse Transcriptase PCR (sqRT-PCR). Eleven transcripts identified as differentially expressed in the microarray experiment were tested using sqRT-PCR (Table 4 and Additional file 7). Genes for sqRT-PCR confirmation were chosen based on differential expression levels in the microarray. We tested genes showing both extreme differential expression and those just exceeding the two-fold criteria. Primers were designed from the EST sequence used to construct the Affymetrix probe to produce 250 bp amplicons. The sqRT-PCR was conducted as described by the Stratagene protocol (Catalog #600532) using the Stratagene Brilliant qRT-PCR kit with 25 uL reactions. For each experimental reaction, 200 ng of total RNA was added as initial template along with 125 mM MgCl_2 _and 100 nM forward and reverse primers. Cycling parameters were as follows: 45 min at 42°C for reverse transcription, 10 min at 95°C to denature reverse transcriptase StrataScript, 40 cycles of 30 sec at 95°C, 1 min at proper annealing temperature, 30 sec at 72°C. All sqRT-PCR reactions were performed in the Stratagene Mx3000P followed by a dissociation curve, taking a fluorescence reading at every degree between 55°C and 95°C to ensure only one PCR product was amplified. As controls, a passive reference dye was added to each reaction to ensure the increase in fluorescence was due to an increase in amplicon and not an artifact of the PCR. Additionally, each sample was run in triplicate and normalized against tubulin amplification to ensure differential expression was not due to differing amounts of initial template RNA added to each sample.

To be considered differentially expressed, samples had to differ in cycle thresholds (Ct) by more than 1 cycle, which corresponds to the two-fold difference in gene transcripts between the NILs identified by the microarray experiment. The resulting fold change of the sqRT-PCR was calculated from the differences in Ct using the 2^Δ^Ct method [47].

### SFP Identification and Association with known IDC QTL on Soybean Genome

Single Feature Polymorphisms (SFPs) were identified following the protocol outlined by West et al. 2006 [48]. In brief, the microarray data from plants grown under iron sufficient conditions was transformed by robust multichip analysis (RMA) [49]. Custom perl scripts were used to examine each of the ten individual probes comprising a single perfect match probe. These perl scripts assigned each perfect mach probe set an SFPdev score by subtracting the average hybridization signal from the other ten probes from the hybridization signal of the probe in question and dividing that by the hybridization signal of the probe being examined ((hyb signal probe 1 - (hyb signal probe 1+ hyb signal probe 2 + hyb signal probe 3 + hyb signal probe 4 ...hyb signal probe 10)/10)/hyb signal probe 1). SFPdev scores with an absolute value greater than or equal to two on all replicates indicated an SFP (Additional file 6).

### Statistical Modeling and Cluster Analysis

To determine if gene distribution along the assembled genome could be explained by random chance, a simulation program originally reported by Grant [50]was applied to a theoretical genome. A genome of 996,903,313 bp (the combined size of the 7x genome assembly which has been assigned to soybean molecular linkage groups) was partitioned into 1,000,000 bp, 100,000 bp, and 10,000 bp windows resulting in 953 bins, 9,530 bins and 95,300 bins respectively. The program positioned 760 or 200 genes depending on the genotype (see below) being simulated on the genome and determined the number of genes within the window. The simulation was repeated 1,000 times. The mean number of bins with 0 – 8 genes was calculated for the 1,000 repetitions. A standard deviation for each gene bin size was also calculated. To determine how this compared with our experimental data, the sequences assigned to chromosomes were concatenated together and the sliding window analysis was performed to identify clusters. Chromosomes are designated as shown in http://www.soybase.org. The difference between the microarray data and the simulated data is calculated in terms of the number of simulated data standard deviations [43]. A difference greater than two SD is considered statistically significant. The sign of the difference is indicative of whether there are more or fewer genes than expected.

### Motif Identification and Analysis

The consensus sequence used by Affymetrix^® ^to generate the probes on the Soybean GeneChip^® ^identified as differentially expressed between Clark plants grown under iron sufficient and iron deficient conditions were queried against the 7X genome gene calls. The top hit for each differentially gene was used as the gene call for the differentially expressed sequence on the Affymetrix^® ^GeneChip^®^. The 835 differentially expressed sequences in the Clark genotype correlated with 760 of the predicted genes in the 7X genome release http://www.phytozome.net while the 200 predicted genes from the IsoClark genotype correlated with 200 predicted genes from the 7X genome. Custom perl scripts identified the 500 bases upstream of the start codon for each gene from the 7X genome assembly. The reverse complement of each of the 500 bp promoter regions was also identified. The program MEME (Multiple Em for Motif Elicitation [51]) was run against the 500 base promoter regions of all IDC genes to identify short conserved sequences in the promoter regions of the differentially expressed genes using the -dna -mod anr -evt 1 commands. Identified motifs with E-values < 1E^-6 ^were then compared against a modified TRANSFAC database using BLASTN [19] to determine if identified motifs contained any known transcription factor binding sites (Table 7).

## Authors' contributions

JAO carried out the sample collection and preparation, RT PCR validation, data analysis, and drafted the manuscript. RTN wrote custom Perl scripts and performed various bioinformatic analyses. SC, JS, and JG performed the genome sequence alignments and assembly. DG performed the genome cluster analysis. CPV. provided comments, suggestions, and revisions to the manuscript. MAG performed various bioinformatic analyses, did the SFP identification, GO category analysis, wrote custom Perl scripts for various analyses, and provided editorial assistance. RCS conceived the study, coordinated the design of the project, and drafted the manuscript. This research was funded in part by the North Central Soybean Research Program.

## Supplementary Material

Additional file 1 **Differentially expressed genes in the Clark genotype comparing plants grown in iron sufficient and iron deficient conditions**. A table of differentially expressed genes in the Clark genotype comparing plants grown in iron sufficient and iron deficient conditions including the identified fold changes and gene annotations. Click here for file

Additional file 2 **Differentially expressed transcripts in the IsoClark genotype between plants grown in iron sufficient and iron deficient conditions**. A table of differentially expressed genes in the IsoClark genotype comparing plants grown in iron sufficient and iron deficient conditions including the identified fold changes and gene annotations. Click here for file

Additional file 3 **Differentially Expressed Genes between Clark and IsoClark genotypes grown under Iron Deficient Conditions**. A table of differentially expressed genes between Clark and IsoClark genotypes grown under iron deficient conditions including the identified fold changes and gene annotations. Click here for file

Additional file 4 **Differentially Expressed Genes in Clusters identified in the Clark genotype with a sliding window of 1,000,000 bases**. A table of differentially expressed genes in the Clark genotype illustrating the identified gene clusters using a sliding window of 1,000,000 bases, their chromosomal location, and gene annotation. Click here for file

Additional file 5 **Differentially Expressed Genes in Clusters identified in the IsoClark genotype with a sliding window of 1,000,000 bases**. A table of differentially expressed genes in the IsoClark genotype illustrating the identified gene clusters using a sliding window of 1,000,000 bases, their chromosomal location, and gene annotation. Click here for file

Additional file 6 **Identified and annotated SFPs between two NILs Soybean Genome Chip consensus sequences**. A table identifying the Affymetrix probes containing a SFP between the Clark and IsoClark genotypes. The data also identifies the chromosome containing the identified SFP and the annotation of the gene containing the SFP. Click here for file

Additional file 7 **Primer sequences used for semi quantitative real time RT-PCR**. A table listing the Affymetrix probe IDs and the associated forward and reverse sequences of the primers used in the semi quantitative real time RT-PCR. Click here for file
