# Supplementary material for: Integrating microarray analysis and the soybean genome to understand the soybeans iron deficiency response
Source: BMC Genomics. 2009 Aug 13;10:376. doi: 10.1186/1471-2164-10-376 (PMC2907705; doi:10.1186/1471-2164-10-376)
Supplement: Additional file 1 — Differentially expressed genes in the Clark genotype comparing plants grown in iron sufficient and iron deficient conditions. A table of differentially expressed genes in the Clark genotype comparing plants grown in iron sufficient and iron deficient conditions including the identified fold changes and gene annotations. [file 1471-2164-10-376-S1.doc]

Additional file 1: Differentially Expressed Genes in the Clark Genotype Comparing Plants grown in Iron Sufficient and Iron Deficient Hydroponics

| Affymetrix Probe ID | Fold Change of Iron Sufficient vs Iron Deficient | Best Hit UniProt ID | In  QTL | Confirmed Annotation | Plant GoSlim |
| --- | --- | --- | --- | --- | --- |
| Gma.1221.1.S1_s_at | -58.948 | P54233 |  | Inducible nitrate reductase [NADH] 1 | response to abiotic or biotic stimulus |
| Gma.8416.1.S1_at | -56.611 | Q9SYR0 |  | Inducible nitrate reductase [NADH] 2 | response to abiotic or biotic stimulus |
| GmaAffx.87317.1.S1_s_at | -50.729 |  |  | No Hits on UniProt |  |
| Gma.12681.1.S1_at | -47.753 | Q9FLI7 |  | Hypothetical Protein | biological process unknown |
| GmaAffx.69544.1.S1_s_at | -41.97 | P05477 |  | class II heat shock protein | response to stress |
| GmaAffx.69544.2.S1_at | -41.131 | P05477 |  | class II heat shock protein | response to stress |
| Gma.17947.1.S1_at | -38.632 | P02519 |  | class I heat shock protein | response to stress |
| GmaAffx.88762.1.S1_at | -35.555 | P32110 |  | Glutathione S-transferase (EC | other cellular |
| GmaAffx.93591.1.S1_s_at | -34.439 | O24320 |  | Lipoxygenase | electron transport or energy pathways |
| Gma.10581.1.S1_at | -33.011 | Q8GUC2 |  | Pphosphatase | other metabolic |
| GmaAffx.69544.1.S1_x_at | -32.548 | P05477 |  | class II heat shock protein | response to stress |
| GmaAffx.82745.1.S1_at | -31.975 |  |  | No Hits on UniProt |  |
| GmaAffx.39719.1.S1_at | -30.862 |  |  | No Hits on UniProt |  |
| GmaAffx.20332.1.A1_at | -25.897 |  |  | No Hits on UniProt |  |
| Gma.3481.1.S1_at | -25.096 | Q29VI1 |  | Patatin-like protein | other metabolic |
| GmaAffx.69544.1.S1_at | -23.841 | P05477 |  | class II heat shock protein | response to stress |
| Gma.10073.1.A1_at | -23.45 | Q8LB81 | * | GDSL-motif lipase/acylhydrolase | other metabolic |
| GmaAffx.89896.1.S1_at | -23.45 | Q2HTU2 |  | class I heat shock protein | response to stress |
| GmaAffx.89246.1.S1_s_at | -23.387 | Q8L683 |  | Lectin precursor | protein metabolism |
| GmaAffx.89697.1.S1_s_at | -22.95 | O23961 |  | Peroxidase | response to stress |
| Gma.17974.1.S1_at | -22.873 | Q43019 | * | Lipid-transfer protein | transport |
| GmaAffx.30771.1.S1_at | -21.843 | Q9FNI1 |  | Cyclin B | biological process unknown |
| Gma.10073.2.S1_at | -21.276 | Q6Y0F0 | * | GSDL-motif lipase | other metabolic |
| GmaAffx.89407.1.A1_s_at | -20.282 | Q1KL62 |  | Non-specific lipid transfer-like protein | transport |
| GmaAffx.93268.1.S1_at | -19.916 | P04794 |  | 17.5 kDa class I heat shock protein | response to stress |
| Gma.15856.2.S1_at | -19.137 | Q84UE0 |  | Apyrase-like protein | other cellular |
| Gma.3043.1.S1_at | -19.111 | Q9S728 |  | Protodermal factor, putative |  |
| Gma.16812.1.S1_s_at | -18.025 | O23961 |  | Peroxidase | response to stress |
| GmaAffx.93553.1.S1_s_at | -17.952 | P38417 |  | Lipoxygenase | response to abiotic or biotic stimulus |
| Gma.2480.1.S1_at | -17.651 | Q6EJD0 |  | 1-deoxy-D-xylulose 5-phosphate reductoisomerase | other cellular |
| GmaAffx.88546.1.S1_at | -17.318 | Q1SYS9 |  | GDSL-like Lipase/Acylhydrolase | other metabolic |
| GmaAffx.90538.1.A1_s_at | -17.002 | Q9FSG7 |  | Thaumatin-like protein 1a precursor | response to stress |
| GmaAffx.15215.1.S1_at | -16.987 | Q9ZRF1 |  | Oxidoreductase/dehydrogenase | response to stress |
| GmaAffx.89896.1.S1_s_at | -16.781 | Q2HTU2 |  | class I heat shock protein (HSP 18.5) | response to stress |
| Gma.2239.1.S1_at | -16.404 | Q43019 |  | Lipid-transfer protein | transport |
| Gma.8522.1.S1_at | -16.346 | Q9LEJ8 |  | Major latex protein homologue | biological process unknown |
| Gma.10763.1.S1_at | -16.236 | Q9XGS6 |  | Cytosolic class II low molecular weight heat shock protein | response to stress |
| GmaAffx.89245.1.S1_s_at | -15.369 |  |  | No Hits on UniProt |  |
| Gma.7766.1.S1_at | -15.278 | P19242 |  | class II heat shock protein | response to stress |
| Gma.12746.1.A1_at | -15.19 | Q9SQJ3 |  | Short-chain alcohol dehydrogenase | other metabolic |
| GmaAffx.766.1.S1_at | -14.991 | O65749 |  | Copper amine oxidase precursor | biological process unknown |
| GmaAffx.73904.1.S1_at | -14.95 | Q94ET0 | * | Hydroxymethylglutaryl coenzyme A synthase | other cellular |
| GmaAffx.77637.1.S1_at | -14.805 | Q2ENC4 |  | Chalcone synthase | other cellular |
| Gma.15610.1.S1_at | -14.714 | Q1T055 |  | MLP-like protein | biological process unknown |
| Gma.6550.2.S1_x_at | -14.455 | Q3SCM5 |  | Caffeic acid O-methyltransferase | other cellular |
| Gma.6550.2.S1_s_at | -14.442 | Q3SCM5 |  | Caffeic acid O-methyltransferase | other cellular |
| Gma.10923.2.S1_at | -13.969 | Q29VI1 |  | Patatin-like protein | other metabolic |
| Gma.16846.1.A1_at | -13.777 | Q9M9Y7 |  | Lipase, putative | other metabolic |
| GmaAffx.90263.1.S1_s_at | -13.375 | P24095 |  | Lipoxygenase | electron transport or energy pathways |
| GmaAffx.51733.1.A1_at | -13.356 | Q1S6Z1 |  | Ribonuclease T2 | developmental |
| Gma.4659.1.S1_at | -13.259 | Q6A174 |  | Copper amine oxidase | biological process unknown |
| HgAffx.12962.1.S1_at | -13.216 |  |  | No Hits on UniProt |  |
| Gma.10689.1.S1_at | -13.213 | P25272 |  | Kunitz-type trypsin inhibitor KTI1 precursor | biological process unknown |
| GmaAffx.93342.1.S1_s_at | -12.953 | Q6A4W8 | * | Glutathione peroxidase | response to stress |
| GmaAffx.71308.1.S1_at | -12.793 | O82042 |  | Heat shock transcription factor (HSFA) |  |
| GmaAffx.13179.1.S1_at | -12.735 | Q1RYE3 |  | Myosin heavy chain like protein | biological process unknown |
| GmaAffx.28120.1.S1_at | -12.569 | Q9ZWS2 |  | Flavonoid 3-O-galactosyl transferase | other metabolic |
| GmaAffx.76187.1.S1_at | -12.491 | Q8LP23 |  | UDP-glucosyltransferase | other metabolic |
| Gma.1594.1.S1_at | -12.32 | P93697 |  | CPRD12 protein | other cellular |
| Gma.18.1.S1_at | -11.904 | Q39819 |  | class IV heat shock protein precursor | response to stress |
| Gma.5496.1.S1_at | -11.873 |  |  | No Hits on UniProt | other metabolic |
| Gma.14554.1.S1_at | -11.84 | Q1SJ63 |  | Hypothetical Protein |  |
| GmaAffx.6142.1.S1_at | -11.628 | Q4ZJ73 |  | 12-oxophytodienoate reductase | response to stress |
| Gma.15996.1.S1_x_at | -11.529 | Q39887 |  | Proline-rich protein | cell organization and biogenesis |
| Gma.10969.1.S1_x_at | -11.526 | Q42780 | * | Lipoxygenase | response to abiotic or biotic stimulus |
| Gma.10216.3.A1_x_at | -11.507 | Q2HTB5 |  | O-methyltransferase, family 2 | other cellular |
| Gma.10969.1.S1_at | -11.419 | Q42780 |  | Lipoxygenase | response to abiotic or biotic stimulus |
| Gma.9202.1.S1_at | -11.368 | Q7M1S6 |  | Trypsin inhibitor p20 | biological process unknown |
| Gma.16500.1.S1_at | -11.363 | P27480 |  | Lipoxygenase 1 | response to abiotic or biotic stimulus |
| GmaAffx.69311.1.S1_at | -11.344 | P04794 |  | 17.5 kDa class I heat shock protein | response to stress |
| Gma.13045.1.S1_at | -11.256 | Q707M7 |  | Acid phosphatase | other cellular |
| Gma.10969.1.S1_a_at | -11.185 | Q42780 |  | Lipoxygenase | response to abiotic or biotic stimulus |
| GmaAffx.70981.1.S1_at | -11.164 | Q3C1F4 |  | Nonsymbiotic hemoglobin | response to stress |
| GmaAffx.93268.1.S1_s_at | -10.97 | P04794 |  | 17.5 kDa class I heat shock protein | response to stress |
| Gma.7580.1.S1_at | -10.796 | Q1SJ63 |  | Hypothetical Protein |  |
| Gma.791.1.S1_at | -10.794 | Q1SSI3 |  | Hypothetical Protein | biological process unknown |
| Gma.9956.1.S1_at | -10.735 | Q6WMU5 |  | Polygalacturonase-inhibiting protein precursor | response to abiotic or biotic stimulus |
| Gma.10216.1.S1_at | -10.637 | Q2HTB5 |  | O-methyltransferase, family 2 | other cellular |
| Gma.2505.1.S1_at | -10.599 | Q94IC4 |  | Ferritin-2, chloroplast precursor | other cellular |
| Gma.7880.1.S1_at | -10.578 | Q1S265 |  | Hypothetical Protein | biological process unknown |
| Gma.12166.1.S1_at | -10.575 | Q599T8 |  | Allene-oxide cyclase precursor | response to stress |
| Gma.18014.2.S1_x_at | -10.422 | Q9S728 |  | Protodermal factor, putative |  |
| Gma.17985.2.S1_at | -10.353 | O64470 |  | Transferase | biological process unknown |
| Gma.13352.1.S1_at | -10.2 | P19976 |  | Ferritin, chloroplast precursor | other cellular |
| Gma.15722.1.S1_at | -10.191 | Q2XV15 |  | Lectin | protein metabolism |
| Gma.13058.1.A1_s_at | -10.172 | Q1RV95 |  | Cytochrome P450 | electron transport or energy pathways |
| GmaAffx.81415.1.S1_at | -10.13 | O24320 |  | Lipoxygenase |  |
| GmaAffx.76826.1.S1_at | -10.095 | Q9STM6 |  | GDSL-like lipase/acylhydrolase | other cellular |
| Gma.18014.1.S1_a_at | -10.03 | Q9S728 |  | Protodermal factor, putative |  |
| Gma.4294.3.S1_a_at | -10.023 | Q1SFJ0 |  | Hypothetical Protein |  |
| Gma.153.1.S1_x_at | -9.984 | O81972 |  | Cytochrome P450 82A2 | electron transport or energy pathways |
| Gma.17985.1.S1_at | -9.627 | Q1T1P6 |  | Transferase | biological process unknown |
| GmaAffx.59009.1.S1_at | -9.617 | Q700A6 |  | Lipid transfer protein GPI-anchored | biological process unknown |
| Gma.5461.1.S1_at | -9.573 | Q53B69 |  | Flavanone 3-hydroxylase | other cellular |
| Gma.529.1.S1_a_at | -9.552 | Q6YGT9 |  | Acid phosphatase | biological process unknown |
| Gma.2220.1.S1_s_at | -9.503 | Q9LLX2 |  | Trypsin inhibitor | biological process unknown |
| GmaAffx.93185.1.S1_s_at | -9.478 | Q9SYM5 |  | Rhamnose biosynthetic enzyme 1 | other cellular |
| Gma.12966.1.S1_at | -9.45 | Q67ZI9 |  | Putative APG protein | other metabolic |
| Gma.2316.1.S1_at | -9.294 | Q9SWY6 | * | 2OG-Fe(II) oxygenase |  |
| GmaAffx.88762.1.S1_x_at | -9.29 | P32110 |  | Glutathione S-transferase | other cellular |
| GmaAffx.85837.1.S1_at | -9.195 | Q5J7N0 |  | Early nodulin gene related protein, putative | other metabolic |
| Gma.10923.1.A1_at | -9.04 | Q29VI1 |  | Patatin-like protein | other metabolic |
| GmaAffx.93243.1.S1_s_at | -8.906 | O65848 |  | Annexin | other biological |
| Gma.17.1.S1_at | -8.784 | Q39820 |  | 22.0 kDa class IV heat shock protein precursor | response to stress |
| Gma.16827.1.S1_at | -8.697 | Q71UA1 |  | Iron-superoxide dismutase | response to abiotic or biotic stimulus |
| Gma.16098.1.A1_at | -8.691 |  |  | No Hits on UniProt |  |
| Gma.13058.1.A1_at | -8.687 | Q1RV95 |  | Cytochrome P450 | electron transport or energy pathways |
| Gma.3617.1.S1_at | -8.647 | Q9ZR88 |  | Bifunctional nuclease (Fragment) | DNA or RNA metabolism |
| GmaAffx.55607.1.S1_at | -8.611 |  |  | No Hits on UniProt | biological process unknown |
| Gma.13140.4.S1_at | -8.591 | Q1SCN9 |  | Aldehyde dehydrogenase (NAD) family protein | other metabolic |
| Gma.1089.1.S1_s_at | -8.581 | Q941G7 |  | Ferritin | other cellular |
| GmaAffx.92499.1.S1_s_at | -8.473 | Q6JYQ9 |  | HEV1.2 | response to stress |
| Gma.6211.2.A1_at | -8.472 | Q84WI7 |  | Glycerophosphoryl diester phosphodiesterase | other cellular |
| Gma.13918.1.A1_at | -8.466 | Q1SSW0 |  | Protein phosphatase 2C | protein metabolism |
| GmaAffx.90617.1.S1_s_at | -8.462 | Q1SWW1 |  | Glycoside hydrolase, family 1 | other metabolic |
| Gma.48.1.S1_at | -8.439 | Q39889 |  | 101 kDa heat shock protein | response to stress |
| Gma.10342.1.S1_at | -8.432 | Q84R94 |  | Hypothetical Protein | biological process unknown |
| Gma.6550.3.S1_s_at | -8.404 | P28002 |  | Caffeic acid 3-O-methyltransferase | other cellular |
| GmaAffx.54632.1.S1_at | -8.336 | Q5J7N0 |  | GSDL-motif lipase | other metabolic |
| Gma.7623.1.A1_at | -8.25 | Q6K237 |  | Hypothetical Protein | other cellular |
| Gma.4305.3.S1_a_at | -8.191 | Q1RWG4 |  | Cyclic nucleotide-gated ion channel 8 | transport |
| Gma.13327.2.S1_a_at | -8.152 | P48399 |  | Chalcone synthase A | other cellular |
| Gma.3026.1.S1_at | -8.118 |  |  | No Hits on UniProt |  |
| GmaAffx.92420.1.S1_s_at | -8.031 | P04793 |  | 17.5 kDa class I heat shock protein | response to stress |
| Gma.12096.1.A1_at | -8.009 |  |  | No Hits on UniProt |  |
| GmaAffx.92620.1.S1_s_at | -7.973 | O81972 |  | Cytochrome P450 | electron transport or energy pathways |
| GmaAffx.93030.1.S1_s_at | -7.967 | Q3LHL1 |  | Short-chain type dehydrogenase/reductase | other metabolic |
| Gma.4457.1.S1_a_at | -7.935 | Q9LZJ5 |  | Multidrug resistance-associated protein 10 | transport |
| Gma.4305.1.S1_at | -7.849 | Q43854 |  | Peroxidase | response to stress |
| Gma.10282.1.A1_at | -7.832 | Q1T3Y4 |  | Small heat shock protein | response to stress |
| Gma.9185.1.S1_at | -7.823 | Q9M8Y5 |  | GDSL-motif lipase/acylhydrolase | other metabolic |
| GmaAffx.85017.1.S1_s_at | -7.822 |  |  | No Hits on UniProt |  |
| GmaAffx.30428.1.S1_at | -7.745 | P26413 |  | Heat shock 70 kDa protein | response to stress |
| GmaAffx.57878.2.S1_at | -7.737 | P93218 |  | Polygalacturonase non-catalytic subunit AroGP3 precursor | biological process unknown |
| GmaAffx.64238.1.S1_at | -7.721 | Q89A90 |  | Cold shock-like protein cspE | transcription |
| GmaAffx.32845.1.A1_at | -7.715 | Q9SKX5 |  | Amine oxidase, putative | electron transport or energy pathways |
| Gma.2185.3.S1_at | -7.64 | Q9FJL3 |  | Peptidylprolyl isomerase | response to stress |
| Gma.1586.1.S1_at | -7.583 | Q948P5 |  | Ferritin-4, chloroplast precursor | other cellular |
| GmaAffx.89772.9.A1_s_at | -7.57 | Q9LEN5 |  | Patellin/ glycoprotein precursor | transport |
| Gma.4366.1.S1_at | -7.536 | Q6F4I6 |  | GSH-dependent dehydroascorbate reductase 1, putative |  |
| Gma.6477.1.S1_at | -7.444 | Q9XFI8 |  | Peroxidase | response to stress |
| Gma.736.1.A1_at | -7.423 | Q8GWQ8 |  | Tryptophan synthase beta chain 2, chloroplast precursor | other metabolic |
| Gma.9069.1.S1_at | -7.413 | Q9SBR4 |  | Geranyl diphosphate synthase small subunit | other cellular |
| GmaAffx.61184.1.S1_at | -7.372 | Q6J540 |  | Cytochrome P450 | electron transport or energy pathways |
| Gma.4558.1.A1_at | -7.299 | Q1S8F9 |  | Lipolytic enzyme, G-D-S-L | other biological |
| Gma.11871.2.S1_at | -7.286 | Q3Y6V1 |  | Cellulose synthase like protein | other cellular |
| GmaAffx.73749.1.S1_at | -7.285 | Q1SD91 |  | Multi antimicrobial extrusion protein MatE | transport |
| Gma.15792.1.S1_at | -7.274 | Q2HW67 |  | CER1 like protein | other cellular |
| GmaAffx.70494.1.A1_at | -7.258 | Q9ARX2 |  | Hypothetical Protein | biological process unknown |
| Gma.17890.1.S1_at | -7.251 |  |  | No Hits on UniProt |  |
| GmaAffx.93393.1.S1_s_at | -7.234 | Q76LA6 |  | Cytosolic ascorbate peroxidase 2 | electron transport or energy pathways |
| Gma.431.2.S1_a_at | -7.234 | O82134 |  | Proliferating cell nuclear antigen | DNA or RNA metabolism |
| Gma.13729.1.A1_at | -7.19 |  | * | No Hits on UniProt |  |
| Gma.529.2.S1_at | -7.176 | Q764C1 |  | Acid phosphatase | biological process unknown |
| Gma.11116.1.S1_at | -7.146 |  |  | No Hits on UniProt |  |
| Gma.11803.1.S1_at | -7.145 | Q1SMR5 |  | Rubredoxin-type Fe(Cys)4 protein | electron transport or energy pathways |
| Gma.13369.1.S1_at | -7.139 | Q7XA30 |  | Serine/threonine kinase | protein metabolism |
| Gma.4386.1.S1_at | -7.132 | Q9ZWQ5 |  | UDP-glucosyl transferase | other metabolic |
| GmaAffx.90343.1.S1_at | -7.128 | Q1T1E0 |  | Gibberellin regulated protein | other biological |
| GmaAffx.93220.1.S1_at | -7.124 | Q52QR3 | * | NAC domain protein NAC3 |  |
| Gma.79.1.S1_s_at | -7.118 | Q39887 |  | Proline-rich protein | cell organization and biogenesis |
| Gma.16812.1.S1_x_at | -7.105 | O23961 |  | Peroxidase | response to stress |
| Gma.431.2.S1_x_at | -7.094 | O82134 |  | Proliferating cell nuclear antigen | DNA or RNA metabolism |
| GmaAffx.40080.1.A1_at | -7.08 | Q1RV95 |  | Putative cytochrome P450 | electron transport or energy pathways |
| Gma.1518.2.S1_a_at | -7.07 | Q8W4L0 |  | Calcium binding protein | biological process unknown |
| GmaAffx.12887.2.S1_at | -7.012 | Q84YI1 |  | Polyphenol oxidase |  |
| Gma.11166.1.S1_x_at | -6.957 | O24320 |  | Lipoxygenase | response to abiotic or biotic stimulus |
| Gma.3433.1.S1_at | -6.918 | Q948Z4 |  | Gibberellin regulated protein | other biological |
| Gma.4457.1.S1_at | -6.918 | Q9LZJ5 |  | Multidrug resistance-associated protein 10 | transport |
| Gma.15715.1.S1_at | -6.901 | Q1SL51 |  | Major intrinsic protein | transport |
| GmaAffx.34532.1.S1_at | -6.877 | Q1RV93 |  | Glycoside hydrolase, family 1 | other metabolic |
| Gma.3539.2.S1_at | -6.868 | Q9STY1 |  | glycerol 3phosphate transporter | transport |
| Gma.17606.2.S1_at | -6.861 | Q1SD30 |  | Transferase | biological process unknown |
| Gma.17702.2.S1_a_at | -6.856 | Q2LAK7 |  | Cytochrome P450 monooxygenase CYP72A1 | electron transport or energy pathways |
| Gma.10216.2.S1_at | -6.852 | Q2HTB5 |  | O-methyltransferase, family 2 | other cellular |
| Gma.3813.1.S1_at | -6.817 | Q1RW87 |  | Hypothetical Protein |  |
| GmaAffx.82240.1.S1_at | -6.815 | Q1S2F4 | * | UDP-glucose glucosyltransferase | response to stress |
| GmaAffx.5414.1.S1_s_at | -6.788 | Q39883 |  | Tonoplast intrinsic protein | transport |
| GmaAffx.3256.2.S1_at | -6.755 | Q8LP22 |  | Flavonol synthase | other cellular |
| Gma.17702.2.S1_x_at | -6.747 | Q2LAK7 |  | Cytochrome P450 monooxygenase | electron transport or energy pathways |
| Gma.12160.1.S1_at | -6.731 | Q5N800 |  | Oxidoreductase | other metabolic |
| Gma.1361.1.S1_at | -6.713 | Q8L9B2 |  | Hypothetical Protein | biological process unknown |
| GmaAffx.80064.1.S1_at | -6.668 | Q93YH3 |  | ATP citrate lyase b-subunit | other cellular |
| GmaAffx.61222.1.S1_s_at | -6.667 | Q1SAQ5 |  | Annexin | biological process unknown |
| Gma.878.1.S1_at | -6.667 | Q8GSL0 |  | Phosphoenolpyruvate carboxylase kinase | protein metabolism |
| Gma.584.3.S1_at | -6.651 | Q94FP3 | * | Succinate dehydrogenase subunit 3 (Fragment) | other cellular |
| GmaAffx.43239.1.S1_at | -6.636 | Q9T072 |  | bHLH transcription factor | transcription |
| Gma.5496.1.S1_s_at | -6.616 |  |  | No Hits on UniProt | other metabolic |
| Gma.10447.2.A1_a_at | -6.598 | Q1T635 | * | Multi antimicrobial extrusion protein (MatE) | transport |
| GmaAffx.72198.1.A1_at | -6.583 | Q8L5C7 |  | UDP glucronosyltransferase | other metabolic |
| Gma.1532.1.S1_at | -6.579 | O49855 |  | Stem 28 kDa glycoprotein precursor | biological process unknown |
| GmaAffx.92964.1.S1_at | -6.569 |  |  | No Hits on UniProt |  |
| Gma.8867.2.S1_at | -6.558 | Q8L5C7 |  | UDP-glucuronosyltransferase | other metabolic |
| Gma.2141.1.S1_at | -6.554 | Q1S2D1 | * | UDP-glucose glucosyltransferase | other metabolic |
| Gma.12487.2.S1_at | -6.549 | Q941F1 | * | Lipase | other metabolic |
| Gma.15856.1.S1_at | -6.541 | Q9FVC2 |  | Apyrase | other cellular |
| Gma.12482.1.A1_at | -6.525 | Q7F2A8 |  | Hypothetical Protein | biological process unknown |
| Gma.9508.3.S1_s_at | -6.514 | Q1SQA1 |  | GDSL-motif lipase/hydrolase-like protein | other metabolic |
| GmaAffx.88571.1.A1_s_at | -6.493 |  |  | No Hits on UniProt |  |
| Gma.1089.1.S1_at | -6.454 | Q941G7 |  | Ferritin | other cellular |
| GmaAffx.45805.2.S1_at | -6.414 | Q3SCM5 |  | Caffeic acid O-methyltransferase | other cellular |
| GmaAffx.60443.1.S1_at | -6.404 | Q9ZST3 |  | Pyrophosphate-dependent phosphofructokinase beta subunit | other cellular |
| Gma.16124.1.S1_at | -6.388 | Q9ZQX0 |  | Albumin 1 precursor |  |
| GmaAffx.43575.1.S1_at | -6.376 | Q9MB25 |  | Pathogenesis-related protein 10 |  |
| GmaAffx.59986.2.S1_at | -6.346 | Q94C37 |  | At1g05230/YUP8H12_16 | transcription |
| Gma.10752.2.S1_at | -6.319 | Q2HUA9 |  | Embryo-specific 3 | biological process unknown |
| Gma.9839.2.S1_at | -6.318 | Q41350 |  | Osmotin-like protein | response to stress |
| Gma.1379.3.S1_at | -6.294 | Q1S753 |  | Gibberellin regulated protein | other biological |
| Gma.17724.3.S1_at | -6.265 | Q5J7N0 |  | GDSL-motif lipase/acylhydrolase | other metabolic |
| Gma.13058.2.S1_at | -6.261 | Q1RV95 |  | Putative cytochrome P450 | electron transport or energy pathways |
| Gma.6211.1.S1_at | -6.249 | Q9FLM1 |  | Hypothetical Protein | other cellular |
| Gma.18041.1.A1_at | -6.242 | Q9FFF6 | * | Leucoanthocyanidin dioxygenase-like protein |  |
| Gma.13905.1.A1_at | -6.233 |  |  | No Hits on UniProt |  |
| Gma.3893.3.S1_at | -6.212 | Q9LMA8 |  | Hypothetical Protein | biological process unknown |
| Gma.5510.2.S1_s_at | -6.212 | Q9XHC6 |  | Cytochrome P450 monooxygenaseCYP93D1 | electron transport or energy pathways |
| Gma.17733.1.S1_s_at | -6.211 | Q1S278 |  | Proteinase inhibitor | response to stress |
| Gma.13796.1.A1_at | -6.211 | Q71BZ3 |  | Type A response regulator | signal transduction |
| Gma.15947.1.S1_at | -6.207 | Q6Z1Z2 | * | HMG type nucleosome/chromatin assembly factor D | transcription |
| Gma.12160.1.S1_a_at | -6.205 | Q5N800 |  | Oxidoreductase | other metabolic |
| GmaAffx.90206.1.S1_s_at | -6.203 | Q9SGP6 |  | Glutaredoxin-like | electron transport or energy pathways |
| Gma.4305.2.S1_a_at | -6.186 | Q43854 |  | Peroxidase | response to stress |
| GmaAffx.89635.1.A1_s_at | -6.179 | O49855 |  | Vegatative storage protein | biological process unknown |
| Gma.9894.1.S1_at | -6.132 | Q2LAJ5 |  | Cytochrome P450 monooxygenase | response to stress |
| GmaAffx.93650.1.S1_s_at | -6.067 | Q6WE90 |  | Polyprotein |  |
| Gma.4331.1.S1_a_at | -6.054 | Q1S9L0 |  | Lipid-transfer protein | transport |
| Gma.4239.2.S1_at | -6.051 | Q947K4 |  | Thiohydroximate S-glucosyltransferase | other metabolic |
| GmaAffx.90703.1.A1_at | -6.038 | Q7XYR7 |  | Peroxidase | response to stress |
| Gma.1008.1.A1_at | -6.025 | Q8RY68 |  | IDS-4-like protein | biological process unknown |
| GmaAffx.12887.1.S1_at | -6.008 | Q84YI1 |  | Polyphenol oxidase |  |
| Gma.3216.3.A1_at | -6.006 | Q8GWZ3 |  | IDS4-like protein | biological process unknown |
| Gma.13140.1.A1_at | -5.985 | Q1SCN9 |  | Aldehyde dehydrogenase (NAD) family protein | other metabolic |
| Gma.3539.1.S1_at | -5.959 | Q9STY1 |  | glycerol 3phosphate transporter, putative | transport |
| Gma.31.1.S1_at | -5.952 | O04840 |  | Nitrite reductase | electron transport or energy pathways |
| GmaAffx.27787.1.A1_at | -5.951 | Q6K4D2 |  | ABC transporter |  |
| GmaAffx.83880.1.A1_at | -5.939 | Q9FY71 |  | Putative phi-1 | biological process unknown |
| GmaAffx.73536.1.S1_at | -5.921 | Q2MJ08 |  | Cytochrome P450 monooxygenase | electron transport or energy pathways |
| Gma.10072.1.S1_at | -5.908 | Q9S746 |  | Adhesion of calyx edges protein ACE (HOTHEAD precursor) | other cellular |
| GmaAffx.1301.59.S1_at | -5.851 | O82090 |  | Annexin | response to stress |
| Gma.17840.1.S1_at | -5.849 | Q1SHX9 |  | Hypothetical Protein |  |
| Gma.9508.2.S1_at | -5.845 | Q1SQA1 |  | Lipolytic enzyme, G-D-S-L | other metabolic |
| Gma.1578.1.S1_at | -5.841 | Q9SKX5 |  | Amine oxidase | electron transport or energy pathways |
| GmaAffx.91749.1.S1_s_at | -5.813 | Q6A171 |  | Polygalacturonase inhibiting protein precursor | response to abiotic or biotic stimulus |
| GmaAffx.90393.1.S1_s_at | -5.811 | Q2L8A7 |  | Acetoacetyl-CoA thiolase | developmental |
| Gma.529.1.S1_x_at | -5.803 | Q6YGT9 |  | Acid phosphatase | biological process unknown |
| GmaAffx.83607.1.S1_at | -5.798 | Q1SUN0 |  | Lipase | other cellular |
| Gma.5786.2.S1_at | -5.794 | Q1SAY6 |  | Proline-rich protein | other metabolic |
| GmaAffx.24879.1.S1_at | -5.787 | Q1SGA4 |  | Hypothetical Protein |  |
| Gma.7771.1.S1_at | -5.771 | Q1S0D0 | * | Glyoxalasee/bleomycin resistance protein | other metabolic |
| GmaAffx.50097.1.S1_at | -5.749 | Q8LB81 |  | GDSL-motif lipase | other metabolic |
| GmaAffx.72944.1.S1_x_at | -5.727 | Q6TXD0 |  | 3-ketoacyl-CoA thiolase | other cellular |
| Gma.6613.2.S1_at | -5.696 | Q42922 |  | Annexin |  |
| GmaAffx.66400.1.A1_s_at | -5.689 |  |  | No Hits on UniProt |  |
| Gma.16877.1.S1_at | -5.683 | Q8H2B0 |  | Beta-amyrin synthase | other metabolic |
| Gma.431.2.S1_at | -5.681 | O82134 |  | Proliferating cell nuclear antigen | DNA or RNA metabolism |
| Gma.1002.2.S1_at | -5.678 | Q9ZWS2 |  | Flavonoid 3-0-glucosyltransferase | other metabolic |
| Gma.3048.1.S1_a_at | -5.659 | Q8W4Z5 |  | Hypothetical Protein |  |
| Gma.3314.1.S1_at | -5.653 | Q6V4X1 |  | Multicystatin (Fragment) | biological process unknown |
| Gma.4630.1.S1_at | -5.63 | Q1KL62 |  | Lipid transfer protein precursor | transport |
| Gma.6942.1.S1_at | -5.625 | Q9ZVN4 |  | Tyrosine specific protein phosphatase family protein |  |
| GmaAffx.80581.1.S1_at | -5.62 | O04840 |  | Nitrite reductase | electron transport or energy pathways |
| GmaAffx.57661.1.S1_at | -5.62 | Q1KSB9 |  | Hypothetical Protein |  |
| Gma.17388.1.S1_at | -5.598 | Q69LA5 | * | Sulfolipid synthase | response to stress |
| GmaAffx.79275.1.S1_s_at | -5.596 | Q2LAJ5 |  | 9/13 hydroperoxide lyase |  |
| GmaAffx.16966.1.S1_at | -5.596 | Q8VXF4 |  | Metallophosphatase | biological process unknown |
| Gma.1614.1.S1_at | -5.594 | Q84J37 |  | Laccase (Diphenol oxidase) family protein | other cellular |
| Gma.11166.1.S1_at | -5.593 | O24320 |  | Lipoxygenase | response to abiotic or biotic stimulus |
| Gma.1649.1.A1_at | -5.581 |  |  | No Hits on UniProt |  |
| GmaAffx.92868.1.S1_s_at | -5.568 | Q6LEG8 |  | Calmodulin | other cellular |
| Gma.12365.2.S1_at | -5.564 |  |  | No Hits on UniProt |  |
| GmaAffx.93212.1.S1_at | -5.551 | Q6WAT9 |  | Lipid transfer protein | other biological |
| Gma.14319.1.A1_at | -5.509 | Q8W1X8 |  | Gamma-glutamyltransferase | biological process unknown |
| GmaAffx.1877.1.A1_at | -5.483 | Q02992 |  | homeodomain transcription factor | response to stress |
| GmaAffx.88681.1.S1_s_at | -5.477 | Q8W1A1 |  | Adenosine 5'-phosphosulfate reductase | other cellular |
| Gma.43.1.A1_at | -5.475 | Q39883 |  | Aquaporin TIP3 | transport |
| GmaAffx.93430.1.S1_s_at | -5.465 | Q9LL80 |  | Purple acid phosphatase 1 |  |
| Gma.8368.1.S1_at | -5.458 | Q40161 |  | Polygalacturonase-1 non-catalytic beta subunit precursor | biological process unknown |
| Gma.8081.1.A1_at | -5.455 | Q39822 |  | Aquaporin-like protein | transport |
| GmaAffx.88681.1.S1_at | -5.455 | Q8W1A1 |  | Adenosine 5'-phosphosulfate reductase | other cellular |
| Gma.3881.2.S1_x_at | -5.439 | P37115 | * | Trans-cinnamate 4-monooxygenase | response to abiotic or biotic stimulus |
| GmaAffx.93511.1.S1_s_at | -5.435 | Q9SDZ0 |  | Isoflavone reductase homolog 2 | biological process unknown |
| GmaAffx.25347.1.S1_at | -5.427 | Q8VWP9 |  | Fiddlehead-like protein | other cellular |
| GmaAffx.24762.1.S1_at | -5.416 | Q9ZPK4 |  | Glutamyl-tRNA reductase | response to abiotic or biotic stimulus |
| Gma.9728.1.S1_at | -5.399 | Q1SD89 |  | Multi antimicrobial extrusion protein MatE | transport |
| Gma.5524.1.S1_at | -5.38 | Q6T1C8 |  | Peroxidase precursor | response to stress |
| Gma.2505.2.S1_x_at | -5.376 | Q94IC4 | * | Ferritin-2, chloroplast precursor | other cellular |
| GmaAffx.40203.1.S1_at | -5.375 | Q2HVN0 |  | Transferase | biological process unknown |
| Gma.8684.1.S1_at | -5.36 | Q8L8A5 |  | GRF1-interacting factor 1 | other cellular |
| Gma.8455.1.S1_at | -5.353 | Q8W3Y5 |  | Flavonoid 3'-hydroxylase | response to abiotic or biotic stimulus |
| Gma.11026.1.S1_at | -5.347 | Q1SMD8 |  | Kunitz inhibitor ST1-like | biological process unknown |
| Gma.1951.2.S1_a_at | -5.341 | Q67Y83 |  | Carboxypeptidase | protein metabolism |
| GmaAffx.3021.1.A1_at | -5.34 |  |  | No Hits on UniProt |  |
| Gma.1518.2.S1_x_at | -5.333 | Q8W4L0 |  | Calcium binding protein | biological process unknown |
| Gma.3881.2.S1_at | -5.327 | P37115 |  | Trans-cinnamate 4-monooxygenase | response to abiotic or biotic stimulus |
| Gma.11035.1.S1_at | -5.314 | Q9LMA8 |  | Hypothetical Protein | biological process unknown |
| GmaAffx.89899.1.S1_s_at | -5.308 | Q2L8A7 |  | Acetoacetyl-CoA thiolase | developmental |
| Gma.2096.2.S1_x_at | -5.307 | Q9ZW03 |  | Tropinone reductase | developmental |
| Gma.957.1.S1_at | -5.264 | Q27JA0 | * | Dirigent like - disease resistane response protein | response to abiotic or biotic stimulus |
| Gma.17032.1.S1_at | -5.263 | Q9AYM8 |  | Reticuline oxidase-like protein | response to stress |
| Gma.1704.1.S1_at | -5.262 | Q9SGP6 |  | Glutaredoxin-like | electron transport or energy pathways |
| GmaAffx.91313.1.S1_s_at | -5.26 | Q7X9B3 |  | 9/13 hydroperoxide lyase | response to stress |
| Gma.12438.1.S1_s_at | -5.247 | Q49RB3 |  | Gibberellin regulated protein | signal transduction |
| Gma.1545.2.S1_at | -5.238 | O82515 |  | NAD-dependent mannitol dehydrogenase |  |
| Gma.15584.2.S1_x_at | -5.229 | Q38JG0 |  | Adenylate kinase family-like protein | electron transport or energy pathways |
| Gma.2505.1.S1_a_at | -5.224 | Q94IC4 |  | Ferritin-2, chloroplast precursor ( | other cellular |
| Gma.1518.1.S1_at | -5.223 | Q2QY10 |  | Calcium binding protein | biological process unknown |
| Gma.9622.1.S1_at | -5.209 | P28002 |  | Caffeic acid 3-O-methyltransferase | other cellular |
| GmaAffx.93041.1.S1_s_at | -5.177 | Q1SSC0 |  | Thaumatin, pathogenesis-related | response to stress |
| GmaAffx.29360.1.S1_s_at | -5.174 |  |  | No Hits on UniProt |  |
| Gma.12616.1.A1_at | -5.173 | Q58GF4 |  | Peroxidase | response to stress |
| GmaAffx.93442.1.S1_at | -5.166 | Q4ZJ73 |  | 12-oxophytodienoate reductase | response to stress |
| Gma.17967.1.S1_at | -5.15 |  |  | No Hits on UniProt |  |
| GmaAffx.90548.1.S1_x_at | -5.141 |  |  | No Hits on UniProt |  |
| GmaAffx.90001.1.S1_at | -5.138 | Q9STG6 | * | dUTP pyrophosphatase | other cellular |
| GmaAffx.87317.1.S1_at | -5.132 |  |  | No Hits on UniProt |  |
| GmaAffx.15861.1.S1_at | -5.13 | Q8LJS8 |  | Homeobox protein | developmental |
| GmaAffx.75820.1.S1_at | -5.126 | Q6ZIK5 |  | Growth-regulating factor 1 |  |
| GmaAffx.8902.1.A1_at | -5.119 | Q1S4Z1 |  | Protein tyrosine kinase, putative | protein metabolism |
| GmaAffx.52941.1.S1_at | -5.111 | Q5J7N0 |  | Early nodulin gene related protein, putative | other metabolic |
| Gma.529.1.S1_at | -5.104 | Q6YGT9 |  | Acid phosphatase | biological process unknown |
| Gma.15564.3.S1_s_at | -5.087 |  |  | No Hits on UniProt |  |
| Gma.15233.1.S1_at | -5.084 | Q8VXF4 |  | Pruple acid phosphatase | biological process unknown |
| Gma.1359.1.S1_at | -5.084 | Q8GUC2 |  | Phosphatase | other metabolic |
| GmaAffx.29025.1.A1_at | -5.084 | Q1SLD2 |  | Homeodomain related |  |
| Gma.4438.3.S1_x_at | -5.08 | Q1T6K5 |  | Flavanone 3-hydroxylase-like protein | other cellular |
| Gma.15048.2.S1_at | -5.062 | Q3S345 |  | Zinc finger protein-like protein | biological process unknown |
| Gma.2096.2.S1_a_at | -5.054 | Q9ZW03 |  | Tropinone reductase | developmental |
| GmaAffx.28861.1.S1_at | -5.043 | Q1S0Z4 |  | Subtilisin serine protease | protein metabolism |
| GmaAffx.26533.1.A1_s_at | -5.041 | Q6V8P5 |  | Hypothetical Protein | biological process unknown |
| GmaAffx.45449.1.S1_at | -5.036 | Q8LAF8 |  | Fatty acid elongase-like protein | biological process unknown |
| GmaAffx.92830.1.S1_s_at | -5.027 |  |  | No Hits on UniProt |  |
| Gma.6452.1.A1_at | -5.026 | O24056 |  | Polyphenol oxidase precursor |  |
| GmaAffx.91725.1.S1_at | -5.015 | Q1SI90 |  | Disease resistance protein; AAA ATPase Leucine Rich Repeat |  |
| GmaAffx.90865.1.S1_s_at | -5.003 | Q39817 |  | Calnexin |  |
| GmaAffx.19480.1.S1_at | -4.99 | P32110 |  | Glutathione S-transferase | other cellular |
| GmaAffx.81366.1.S1_at | -4.99 | Q9LYC1 | * | Gibberellin receptor | biological process unknown |
| Gma.1917.1.S1_s_at | -4.98 | Q9FQE8 |  | Glutathione S-transferase | response to stress |
| GmaAffx.79990.1.S1_s_at | -4.979 | O48781 |  | Ids4-like protein | biological process unknown |
| GmaAffx.16212.1.S1_at | -4.972 | P15490 |  | Stem 28 kDa glycoprotein precursor | biological process unknown |
| Gma.4462.1.S1_at | -4.957 | Q6AVB5 |  | Vegetative storage prtein, | other cellular |
| GmaAffx.93250.1.S1_at | -4.935 | Q93YH4 |  | ATP citrate lyase a-subunit | other cellular |
| HgAffx.12572.1.S1_at | -4.92 |  |  | No Hits on UniProt |  |
| Gma.14471.4.S1_x_at | -4.914 | Q506K1 |  | Aquaporin | transport |
| GmaAffx.64197.1.S1_at | -4.912 | P34899 |  | Serine hydroxymethyltransferase, mitochondrial precursor ( | other cellular |
| GmaAffx.82887.1.S1_at | -4.912 | Q9SYM5 |  | Rhamnose biosynthetic enzyme 1 | other cellular |
| GmaAffx.71692.1.S1_at | -4.908 | Q1STD8 |  | UDP-glucuronosyl/UDP-glucosyltransferase | response to stress |
| Gma.2342.1.S1_at | -4.906 | Q43457 |  | Heat shock transcription factor | response to stress |
| Gma.5831.3.S1_s_at | -4.905 | Q944T2 |  | Translationally controlled tumor protein homolog | biological process unknown |
| Gma.15584.2.S1_at | -4.901 | Q38JG0 |  | Adenylate kinase family-like protein | electron transport or energy pathways |
| GmaAffx.23381.1.S1_at | -4.901 | Q9SD82 |  | Replication protein A1 | DNA or RNA metabolism |
| GmaAffx.89678.1.S1_s_at | -4.874 |  |  | No Hits on UniProt |  |
| GmaAffx.87441.1.S1_at | -4.861 | Q1S2D1 |  | UDP-glucose glucosyltransferase | response to stress |
| Gma.5141.1.S1_at | -4.854 | O22917 | * | Laccase |  |
| Gma.15996.1.S1_at | -4.844 | Q39887 |  | Proline-rich protein | cell organization and biogenesis |
| Gma.8472.1.S1_at | -4.834 | P31687 |  | 4-coumarate--CoA ligase 2 | response to abiotic or biotic stimulus |
| Gma.436.1.S1_at | -4.828 | Q07502 |  | Kunitz trypsin inhibitor protein | response to abiotic or biotic stimulus |
| GmaAffx.92117.1.S1_s_at | -4.814 | Q9FUK4 |  | Cytosolic glutamine synthetase | other metabolic |
| GmaAffx.91167.1.S1_s_at | -4.812 | Q9LS40 |  | CND41, chloroplast nucleoid DNA binding protein-like | protein metabolism |
| GmaAffx.92244.1.S1_at | -4.806 | O81016 |  | Pleiotropic drug resistance protein | transport |
| Gma.12567.1.S1_at | -4.793 | Q8LD01 |  | Lipase/hydrolase, putative | other metabolic |
| GmaAffx.277.1.S1_at | -4.789 | Q66PF2 |  | UDP-glucosyltransferase |  |
| Gma.3314.1.S1_a_at | -4.785 | Q6V4X1 |  | Multicystatin | biological process unknown |
| Gma.6602.2.S1_s_at | -4.777 | Q6EJD1 |  | Isopentenyl-diphosphate delta-isomerase, type 1 | other cellular |
| Gma.9221.1.S1_at | -4.72 | Q1SNG7 |  | Glycoside hydrolase, family 18 | other metabolic |
| GmaAffx.81892.1.S1_at | -4.707 | Q9ZRF1 |  | NAD-dependent mannitol dehydrogenase | response to abiotic or biotic stimulus |
| AFFX-Gm_P450_M_s_at | -4.701 |  |  | No Hits on UniProt |  |
| Gma.17825.1.A1_at | -4.697 | Q1S8F6 |  | Lipolytic enzyme, G-D-S-L | other biological |
| Gma.12974.1.S1_at | -4.693 | Q1SN75 |  | Beta-Ig-H3/fasciclin | other cellular |
| Gma.13402.1.A1_at | -4.682 | Q75HS3 |  | Crooked neck protein | other cellular |
| GmaAffx.69470.1.S1_at | -4.657 |  |  | No Hits on UniProt |  |
| Gma.3880.1.S1_at | -4.653 | O64865 |  | Hypothetical Protein | transport |
| GmaAffx.79567.1.A1_at | -4.645 |  | * | No Hits on UniProt |  |
| GmaAffx.72944.1.S1_at | -4.645 | Q6TXD0 |  | 3-ketoacyl-CoA thiolase | other cellular |
| Gma.7599.3.S1_at | -4.629 | Q9M381 | * | Mevalonate disphosphate decarboxylase | other cellular |
| GmaAffx.89049.1.S1_s_at | -4.621 | Q1SAQ5 |  | Annexin, | biological process unknown |
| Gma.338.2.S1_s_at | -4.62 | Q9XFI6 |  | Peroxidase | response to stress |
| GmaAffx.15794.1.S1_at | -4.615 | Q5MJZ4 |  | 1-deoxy-D-xylulose-5-phosphate synthase | other cellular |
| Gma.13350.1.S1_x_at | -4.606 | O22639 |  | Luminal-binding protein precursor | response to stress |
| GmaAffx.23059.1.S1_at | -4.598 | Q9ZWS2 |  | Hypothetical Protein | other metabolic |
| GmaAffx.57970.1.S1_at | -4.598 | Q52QR3 | * | NAC domain protein NAC3 | developmental |
| Gma.8020.2.S1_at | -4.594 | Q68IP6 |  | Allene oxide cyclase C4 | response to stress |
| Gma.2505.2.S1_at | -4.576 | Q94IC4 |  | Ferritin-2, chloroplast precursor | other cellular |
| GmaAffx.86334.2.S1_at | -4.571 | Q2PJB7 |  | Latex allene oxide synthase | response to stress |
| Gma.11215.1.S1_at | -4.567 |  |  | No Hits on UniProt |  |
| Gma.13327.1.S1_at | -4.561 | Q2ENC2 |  | Chalcone synthase | other cellular |
| GmaAffx.77177.1.S1_at | -4.556 | Q2LAK7 |  | Cytochrome P450 monooxygenase CYP72A1 | electron transport or energy pathways |
| Gma.5089.1.S1_at | -4.542 | Q94EY7 |  | Hypothetical Protein | biological process unknown |
| Gma.10151.1.S1_at | -4.542 |  |  | No Hits on UniProt |  |
| Gma.3289.1.S1_at | -4.54 | Q1S0T2 |  | NADH dehydrogenase 1 alpha subunit |  |
| GmaAffx.88982.1.A1_s_at | -4.53 | Q3Y6V1 |  | Cellulose synthase isolog | other cellular |
| Gma.13994.1.A1_at | -4.527 | Q1S1C2 |  | Chalcone and stilbene synthases, N-terminal | other metabolic |
| GmaAffx.25607.1.A1_at | -4.521 |  |  | No Hits on UniProt |  |
| Gma.4468.1.S1_at | -4.521 | Q2L8A7 |  | Acetyl-CoA acetyltransferase, cytosolic 1 | developmental |
| Gma.5632.1.S1_at | -4.511 | Q1S608 |  | ML domain protein | biological process unknown |
| Gma.838.1.S1_at | -4.508 | Q2MCJ4 | * | Beta-D-xylosidase | other metabolic |
| GmaAffx.84474.1.S1_at | -4.481 | Q8LBR3 |  | Alcohol dehydrogenase |  |
| Gma.6281.1.S1_at | -4.473 | Q7X9B3 |  | 9/13 hydroperoxide lyase | response to stress |
| Gma.4739.1.S1_at | -4.471 | Q8LA20 | * | Hypothetical Protein | biological process unknown |
| Gma.3216.2.S1_at | -4.468 | O48781 |  | IDS4-like protein | biological process unknown |
| Gma.1165.1.S1_at | -4.465 | Q1SE79 |  | BRH1 RING finger protein |  |
| GmaAffx.3256.1.S1_at | -4.464 | Q8LP22 |  | Flavonol synthase | other cellular |
| Gma.3189.1.S1_a_at | -4.462 | Q1SJ01 |  | Serine/threonine protein kinase, active site | protein metabolism |
| Gma.17768.1.A1_at | -4.46 | Q1RV93 |  | Glycoside hydrolase, family 1 | other metabolic |
| Gma.10482.2.S1_a_at | -4.454 | Q6UEJ2 |  | Mini-chromosome maintenance 7 | DNA or RNA metabolism |
| GmaAffx.55661.1.S1_at | -4.43 | Q9FGX1 |  | ATP-citrate lyase subunit B | other cellular |
| GmaAffx.75004.1.S1_at | -4.429 | Q94C37 | * | Homeobox protein | transcription |
| Gma.13342.1.A1_at | -4.425 | P52581 |  | Isoflavone reductase homolog | biological process unknown |
| Gma.5018.1.S1_at | -4.41 | Q1SXK5 |  | ATP citrate synthase, small subunit | other cellular |
| GmaAffx.81791.1.S1_at | -4.41 | Q9FY93 |  | NAM-like protein | developmental |
| Gma.612.1.A1_at | -4.398 | Q6SA75 |  | Hypothetical Protein |  |
| GmaAffx.82936.1.S1_at | -4.373 | Q2PER5 |  | CT099 | electron transport or energy pathways |
| GmaAffx.73659.1.S1_at | -4.372 |  |  | No Hits on UniProt |  |
| GmaAffx.83079.1.S1_at | -4.36 |  |  | No Hits on UniProt |  |
| Gma.8020.2.S1_a_at | -4.365 | Q68IP6 |  | Allene oxide cyclase C4 | response to stress |
| Gma.5649.3.S1_a_at | -4.358 | Q38JJ2 |  | Disulfide-isomerase-like protein | electron transport or energy pathways |
| GmaAffx.90937.1.A1_at | -4.354 | P24095 |  | Seed lipoxygenase | response to abiotic or biotic stimulus |
| GmaAffx.87089.1.S1_at | -4.346 | Q60D21 |  | Hypothetical Protein | biological process unknown |
| GmaAffx.90548.1.S1_s_at | -4.34 |  |  | No Hits on UniProt |  |
| GmaAffx.70608.1.S1_at | -4.337 | Q1S3G7 |  | UDP-glucuronosyl/UDP-glucosyltransferase | response to stress |
| GmaAffx.93161.1.S1_s_at | -4.336 | Q1SQB5 |  | Actin | cell organization and biogenesis |
| GmaAffx.93522.1.S1_s_at | -4.33 | Q9M631 |  | Cinnamoyl-CoA reductase | other cellular |
| Gma.1345.1.S1_at | -4.32 |  |  | No Hits on UniProt |  |
| GmaAffx.48718.1.S1_at | -4.318 |  |  | No Hits on UniProt |  |
| Gma.14850.1.S1_at | -4.316 | Q1S104 |  | Embryo-specific 3 | biological process unknown |
| Gma.2635.1.A1_at | -4.315 | Q6JJ29 |  | Prephenate dehydratase | other cellular |
| Gma.6540.1.S1_at | -4.313 | Q1S8H7 |  | Alcohol dehydrogenase superfamily, zinc-containing |  |
| GmaAffx.70258.1.S1_s_at | -4.306 |  |  | No Hits on UniProt |  |
| GmaAffx.72179.1.A1_at | -4.306 | O65739 |  | Nucleoid DNA binding like protein | protein metabolism |
| GmaAffx.56975.1.S1_at | -4.305 | Q1RY98 |  | Hydroxymethylglutaryl-coenzyme A reductase | other cellular |
| Gma.795.1.A1_at | -4.301 |  |  | No Hits on UniProt |  |
| Gma.1110.1.S1_at | -4.293 | Q66PF2 |  | Anthocyanidine rhamosyl transferase | other metabolic |
| Gma.13015.1.A1_at | -4.291 | Q7Y0W0 |  | Outer cell layer homeo domain protein, putative | transcription |
| GmaAffx.86334.1.S1_at | -4.291 | Q2LAJ7 |  | Cytochrome P450 monooxygenase CYP74C | response to stress |
| GmaAffx.89971.1.S1_s_at | -4.285 | Q9ZR53 |  | Annexin | other biological |
| GmaAffx.62364.1.S1_at | -4.284 | Q9FG66 |  | Aminopeptidase C |  |
| GmaAffx.60849.1.A1_at | -4.284 | Q8S995 | * | Glucosyltransferase | response to stress |
| GmaAffx.51326.1.S1_at | -4.272 | Q1SCJ7 |  | Glycerol-3-phosphate acyltransferase 4 | other metabolic |
| Gma.2086.1.S1_at | -4.255 | O49046 | * | Arginase | other cellular |
| GmaAffx.80375.1.S1_at | -4.243 | Q1SMA6 |  | Hypothetical Protein |  |
| Gma.16984.1.A1_at | -4.239 | Q1RW87 |  | Hypothetical Protein | biological process unknown |
| GmaAffx.86773.1.S1_s_at | -4.239 | Q1RYR2 |  | Phenylalanine/histidine ammonia-lyase | response to abiotic or biotic stimulus |
| GmaAffx.93250.1.S1_s_at | -4.239 | Q93YH4 |  | ATP citrate lyase a-subunit | other cellular |
| GmaAffx.80073.1.S1_at | -4.239 | Q70EW1 |  | Copper amine oxidase | biological process unknown |
| GmaAffx.47157.1.A1_at | -4.236 | Q8LJU3 |  | L-ascorbate oxidase precursor |  |
| Gma.11299.3.S1_a_at | -4.236 | Q2XPW6 |  | NAD-dependent epimerase/dehydratase family protein | other cellular |
| Gma.9832.1.S1_at | -4.235 | Q8L9J9 | * | Hypothetical Protein | biological process unknown |
| Gma.9839.1.S1_at | -4.233 | Q41350 |  | Osmotin-like protein | response to stress |
| Gma.13350.1.S1_s_at | -4.23 | O22639 |  | Luminal-binding protein precursor | response to stress |
| GmaAffx.42517.1.A1_at | -4.227 | Q1RVA3 |  | Hypothetical Protein |  |
| Gma.16290.1.S1_s_at | -4.215 | Q9FX61 | * | Hypothetical Protein | biological process unknown |
| Gma.6602.1.S1_x_at | -4.213 | Q6EJD1 |  | Isopentenyl pyrophosphate isomerase | other cellular |
| GmaAffx.27192.2.S1_s_at | -4.207 | Q9S750 |  | Thymidine kinase | electron transport or energy pathways |
| GmaAffx.88786.1.A1_s_at | -4.192 | Q40313 |  | Caffeoyl-CoA O-methyltransferase |  |
| GmaAffx.76520.1.S1_at | -4.191 | P35016 |  | Heat shock protein 90 | protein metabolism |
| GmaAffx.91168.1.S1_at | -4.176 | O23961 |  | Peroxidase precursor | response to stress |
| Gma.1715.1.S1_at | -4.176 | Q9AYM7 |  | GDSL-motif lipase/hydrolase | other metabolic |
| GmaAffx.5510.1.S1_s_at | -4.171 |  |  | No Hits on UniProt |  |
| GmaAffx.85292.1.S1_at | -4.162 | Q6Z671 |  | Chloroplast nucleoid DNA-binding protein cnd41 | protein metabolism |
| GmaAffx.37281.1.S1_at | -4.161 | Q1S047 | * | Minichromosomal maintenance factor | DNA or RNA metabolism |
| Gma.15113.1.S1_a_at | -4.161 | Q8RU51 |  | Glucan 1,3-beta-glucosidase |  |
| Gma.3189.1.S1_at | -4.155 | Q1SJ01 |  | Serine/threonine protein kinase, active site | protein metabolism |
| GmaAffx.60035.1.S1_at | -4.153 | P93217 |  | Polygalacturonase non-catalytic subunit AroGP2 precursor | biological process unknown |
| Gma.385.1.S1_at | -4.143 | Q9ZR53 |  | Annexin-like protein | other biological |
| GmaAffx.89821.1.A1_at | -4.132 | Q2I0H4 |  | Glyceraldehyde-3-phosphate dehydrogenase | other cellular |
| GmaAffx.66617.1.S1_at | -4.129 | O82432 |  | Leucine-rich repeat receptor-like protein kinase | protein metabolism |
| GmaAffx.39734.2.S1_at | -4.123 | O81348 |  | bHLH transcription factor | transcription |
| GmaAffx.58559.1.A1_at | -4.121 | Q1S6U2 |  | Anthranilate N-hydroxycinnamoyl/benzoyltransferase | protein metabolism |
| GmaAffx.89049.1.S1_at | -4.113 | Q1SAQ5 |  | Annexin, | biological process unknown |
| Gma.11035.1.S1_s_at | -4.113 | Q9LMA8 |  | Hypothetical Protein | biological process unknown |
| GmaAffx.72444.1.S1_at | -4.097 | Q5ZF77 |  | Plasma membrane intrinsic protein | transport |
| GmaAffx.72720.1.S1_at | -4.084 | Q58J25 |  | Phosphate/phosphoenolpyruvate translocator | transport |
| Gma.10666.1.S1_at | -4.082 | Q7G753 | * | Alcohol dehydrogenase |  |
| Gma.6231.1.S1_at | -4.08 | Q8RVX2 |  | Protease inhibitor | biological process unknown |
| GmaAffx.87400.1.S1_at | -4.074 | Q94CH6 |  | Lipolytic enzyme, GDSL | other biological |
| GmaAffx.17924.1.S1_at | -4.07 | Q1SED4 |  | Alpha/beta hydrolase fold | other cellular |
| GmaAffx.92142.1.S1_s_at | -4.062 | Q9SE94 |  | Methylenetetrahydrofolate reductase 1 | other cellular |
| GmaAffx.75159.1.S1_x_at | -4.062 | Q1SQB5 |  | Actin | cell organization and biogenesis |
| PsAffx.C45000050_at | -4.058 |  |  | No Hits on UniProt |  |
| Gma.4563.2.S1_at | -4.055 | Q41101 |  | Phaseolin G-box binding protein PG1 | transcription |
| Gma.17292.1.A1_s_at | -4.052 | Q8LDY8 |  | Hypothetical Protein | biological process unknown |
| Gma.4589.2.S1_at | -4.039 | Q1RY98 |  | 3-hydroxy-3-methylglutaryl-coenzyme A reductase 3 | other cellular |
| GmaAffx.45122.1.S1_at | -4.033 | Q6QNI1 |  | Cytochrome P450 | electron transport or energy pathways |
| Gma.10482.1.A1_a_at | -4.024 | Q6UEJ2 |  | Replication licensing factor MCM7 homologue | DNA or RNA metabolism |
| GmaAffx.25551.1.S1_at | -4.017 | Q9LK44 |  | Hypothetical Protein | biological process unknown |
| Gma.11299.3.S1_x_at | -4.016 | Q2XPW6 |  | NAD-dependent epimerase/dehydratase family protein | other cellular |
| GmaAffx.38907.1.S1_at | -4.012 | Q1SH82 |  | Lipase | other metabolic |
| Gma.10234.1.S1_at | -4.008 |  |  | No Hits on UniProt |  |
| GmaAffx.34785.7.S1_s_at | -4.001 |  |  | No Hits on UniProt |  |
| Gma.153.1.S1_at | -3.996 | O81972 |  | Cytochrome P450 | electron transport or energy pathways |
| Gma.15683.1.S1_at | -3.996 | Q9SC38 |  | Germin-like protein | biological process unknown |
| GmaAffx.13543.1.A1_at | -3.994 | O82074 |  | Beta-D-glucosidase precursor | other metabolic |
| GmaAffx.62256.1.S1_at | -3.992 | Q2PEZ2 |  | Nucleoid DNA binding like protein | protein metabolism |
| Gma.8020.3.S1_at | -3.99 | Q599T8 |  | Allene oxide cyclase C4 | response to stress |
| GmaAffx.46567.1.S1_at | -3.99 | Q9FJ26 |  | DNA polymerase alpha subunit IV (Primase)-like protein | DNA or RNA metabolism |
| GmaAffx.93603.1.S1_s_at | -3.982 | P19976 |  | Ferritin-1, chloroplast precursor | other cellular |
| GmaAffx.89783.1.S1_s_at | -3.982 | Q8W2E3 |  | Hydroxymethylglutaryl-coenzyme A reductase | other cellular |
| Gma.9961.1.S1_at | -3.977 | P33083 |  | Auxin-induced protein | other biological |
| Gma.6427.3.S1_a_at | -3.977 | Q39817 |  | Calnexin homolog precursor |  |
| GmaAffx.52252.1.A1_at | -3.975 |  |  | No Hits on UniProt |  |
| Gma.3730.2.S1_a_at | -3.972 | Q2PJR9 |  | Transcription factor WRKY4 | transcription |
| Gma.17785.1.S1_at | -3.967 | Q9ZR83 |  | Ethylene-responsive element binding protein homolog | transcription |
| GmaAffx.92441.1.S1_s_at | -3.961 | Q1SWS7 |  | TIR | response to abiotic or biotic stimulus |
| GmaAffx.21045.1.A1_at | -3.96 |  |  | No Hits on UniProt |  |
| Gma.300.1.A1_s_at | -3.959 | Q1RU46 |  | Response regulator | signal transduction |
| Gma.12599.1.S1_at | -3.958 | P42814 |  | S-like RNAse 28 | developmental |
| Gma.12658.1.A1_at | -3.954 |  |  | No Hits on UniProt |  |
| Gma.10447.1.S1_at | -3.952 | Q1T635 |  | Multi antimicrobial extrusion protein (MatE) | transport |
| Gma.5712.1.S1_s_at | -3.941 | Q1S0T2 |  | NADH dehydrogenase 1 alpha subunit |  |
| GmaAffx.90450.1.S1_at | -3.931 | Q1SD74 |  | Hypothetical Protein | other cellular |
| GmaAffx.75159.1.S1_at | -3.93 | Q1SQB5 |  | Actin | cell organization and biogenesis |
| GmaAffx.86609.1.S1_at | -3.925 | Q9FJT8 |  | Histone acetyltransferase type B catalytic subunit | biological process unknown |
| Gma.7646.2.S1_at | -3.924 | Q9FHM8 |  | Receptor-like protein kinase | protein metabolism |
| GmaAffx.62941.1.S1_at | -3.923 | Q9SZ28 |  | STIG1 protein | biological process unknown |
| Gma.10687.1.S1_at | -3.922 | O65759 |  | Histone H2AX | cell organization and biogenesis |
| Gma.17607.2.S1_s_at | -3.921 |  |  | No Hits on UniProt |  |
| GmaAffx.83981.1.S1_at | -3.902 | Q9LFB4 |  | Sulfolipid synthase | response to stress |
| GmaAffx.25369.1.S1_s_at | -3.901 |  | * | No Hits on UniProt |  |
| Gma.16576.2.S1_a_at | -3.898 | P46266 |  | 14-3-3-like protein |  |
| Gma.1127.1.S1_at | -3.898 |  |  | No Hits on UniProt |  |
| Gma.3183.3.S1_at | -3.891 | Q8H9B2 |  | T-complex protein 1 | protein metabolism |
| Gma.17130.1.S1_at | -3.882 | Q1RTA0 |  | UDP-glucose-fructose-phosphate glucosyltransferase |  |
| Gma.6128.1.S1_at | -3.879 | Q8LEX2 |  | Hypothetical Protein | transport |
| GmaAffx.52029.1.S1_at | -3.877 | Q8H0G9 |  | Minichromosomal maintenance factor | DNA or RNA metabolism |
| Gma.15818.1.S1_at | -3.876 | Q1S8S7 |  | Hypothetical Protein |  |
| Gma.6331.1.S1_at | -3.869 | Q6J8X2 |  | Cellulose synthase | other cellular |
| Gma.13370.1.A1_at | -3.866 | Q1S8F9 |  | Lipolytic enzyme, G-D-S-L | other biological |
| GmaAffx.72251.1.S1_at | -3.86 | Q7XZT4 |  | Replication protein A1 | response to stress |
| Gma.12400.1.S1_at | -3.858 | Q1RVM0 |  | Hypothetical Protein | biological process unknown |
| Gma.7642.1.A1_at | -3.855 | Q1SC78 |  | Ammonium transporter | response to abiotic or biotic stimulus |
| GmaAffx.83638.1.S1_at | -3.851 | Q1SMQ2 |  | Hypothetical Protein | biological process unknown |
| GmaAffx.42249.1.S1_at | -3.844 | Q6T367 |  | Centromeric histone | cell organization and biogenesis |
| GmaAffx.68853.1.S1_at | -3.843 | Q8VY60 |  | Hypothetical Protein | biological process unknown |
| Gma.406.4.S1_s_at | -3.841 | Q1SFW5 |  | S-adenosylmethionine synthetase | other cellular |
| Gma.10032.1.S1_at | -3.839 | Q9FQF1 |  | Glutathione S-transferase | other cellular |
| GmaAffx.24470.1.S1_at | -3.836 | Q8LJC6 |  | UDP-glucosyltransferase | other metabolic |
| GmaAffx.93541.2.S1_at | -3.83 | Q93Z89 |  | Peptidase, metallopeptidases | protein metabolism |
| GmaAffx.377.1.S1_at | -3.824 | Q2HTL9 |  | Mevalonate kinase/phosphomevalonate kinase | other cellular |
| GmaAffx.73405.1.S1_at | -3.822 |  |  | No Hits on UniProt |  |
| GmaAffx.81688.1.S1_at | -3.82 | Q9SK66 |  | NADH dehydrogenase |  |
| Gma.8220.1.S1_at | -3.817 | Q1SWY0 |  | Uroporphiryn-III C-methyltransferase | other metabolic |
| GmaAffx.59053.1.S1_at | -3.817 | Q9LUH2 |  | MATE efflux like protein | response to abiotic or biotic stimulus |
| GmaAffx.41396.1.S1_s_at | -3.816 |  |  | No Hits on UniProt |  |
| GmaAffx.88024.1.A1_at | -3.816 | Q9M1S9 |  | Hypothetical Protein | DNA or RNA metabolism |
| Gma.5744.2.S1_at | -3.812 | Q1RXS8 |  | Pectinesterase |  |
| GmaAffx.87031.1.S1_at | -3.811 | O24093 |  | L-ascorbate oxidase precursor |  |
| Gma.2450.1.A1_at | -3.807 | Q43716 |  | UDP-glucosyltransferase | other metabolic |
| Gma.702.1.S1_at | -3.797 | P93698 |  | Loxc homolog (lipoxygenase) | response to stress |
| GmaAffx.93405.1.S1_s_at | -3.795 | Q9XFG0 |  | Cystathionine-gamma-synthase precursor | other cellular |
| Gma.1907.1.S1_at | -3.786 | Q1SDJ4 |  | Lipolytic enzyme, G-D-S-L | other metabolic |
| GmaAffx.61513.1.A1_at | -3.781 |  |  | No Hits on UniProt |  |
| Gma.8466.1.S1_at | -3.779 | Q43458 |  | Heat shock transcription factor | transcription |
| Gma.16292.2.S1_a_at | -3.778 | Q6EP31 |  | Rac-like GTP-binding protein | other biological |
| GmaAffx.86552.2.S1_at | -3.776 | Q501D5 |  | Hypothetical Protein | biological process unknown |
| Gma.16912.1.S1_at | -3.775 | O81297 |  | Hypothetical Protein | biological process unknown |
| Gma.8556.1.S1_at | -3.774 | Q1S5G3 |  | Proline/glycine betaine transporter | transport |
| GmaAffx.91641.1.S1_s_at | -3.77 | Q8S5C1 |  | 4-coumarate:CoA ligase isoenzyme 2 | other cellular |
| Gma.10709.2.S1_a_at | -3.766 | Q9ZS51 |  | Peroxisomal membrane protein PMP22 | biological process unknown |
| GmaAffx.82528.2.S1_at | -3.758 | Q45NI7 |  | Hypothetical Protein | biological process unknown |
| GmaAffx.91768.1.S1_s_at | -3.751 | Q2PJR9 |  | WRKY transcription factor | transcription |
| GmaAffx.32647.1.S1_at | -3.75 | Q6UEJ2 |  | Minichromosomal maintenance factor | DNA or RNA metabolism |
| Gma.9611.1.A1_at | -3.748 | Q6QUQ3 |  | Auxin and ethylene responsive GH3-like protein | other biological |
| Gma.11601.1.A1_at | -3.737 | Q1SDG3 |  | Phosphoinositide-specific phospholipase C | signal transduction |
| GmaAffx.88173.1.S1_at | -3.734 | Q9ASV9 |  | Hypothetical Protein |  |
| Gma.16787.1.S1_at | -3.734 | Q1SQA1 |  | Lipase/hydrolase, putative | other metabolic |
| GmaAffx.64305.1.S1_s_at | -3.732 |  |  | No Hits on UniProt |  |
| Gma.16484.1.S1_at | -3.73 | Q8W3K5 |  | Kunitz-type trypsin inhibitor KTI1 precursor | biological process unknown |
| Gma.4563.1.A1_at | -3.726 | Q41101 |  | Phaseolin G-box binding protein PG1 | transcription |
| GmaAffx.58494.1.S1_at | -3.719 | Q9SLF1 |  | Nodulin-like protein |  |
| GmaAffx.42044.1.A1_at | -3.717 |  |  | No Hits on UniProt |  |
| Gma.12547.1.A1_at | -3.717 | Q94KS0 |  | Histidine-containing phosphotransfer protein | signal transduction |
| GmaAffx.63832.1.S1_at | -3.717 | Q9FHS0 |  | Histone like transcription factor, putative | transcription |
| Gma.13259.2.S1_a_at | -3.711 |  |  | No Hits on UniProt | developmental |
| Gma.4549.1.S1_s_at | -3.705 | Q1SXA5 |  | Stromal cell-derived factor 2-like protein | biological process unknown |
| Gma.11453.1.S1_at | -3.703 | Q2HT39 |  | Wax synthase isoform | biological process unknown |
| GmaAffx.51983.1.S1_at | -3.702 |  |  | No Hits on UniProt | signal transduction |
| Gma.2776.2.S1_s_at | -3.699 | Q8W3Y3 |  | 1-aminocyclopropane-1-carboxylic acid oxidase | other cellular |
| GmaAffx.34785.8.S1_s_at | -3.698 |  |  | No Hits on UniProt |  |
| PsAffx.C16000098_at | -3.697 |  |  | No Hits on UniProt |  |
| GmaAffx.87358.1.S1_at | -3.695 |  |  | No Hits on UniProt |  |
| Gma.6602.1.S1_at | -3.694 | Q6EJD1 |  | Isopentenyl pyrophosphate isomerase | other cellular |
| GmaAffx.91743.1.S1_s_at | -3.691 | Q9SRM4 | * | Nucleic acid binding protein | transcription |
| GmaAffx.30749.1.S1_at | -3.688 | O81226 |  | Glutamine cyclotransferase-like protein | biological process unknown |
| Gma.18018.1.S1_at | -3.683 | Q1S9D3 |  | Protein kinase | protein metabolism |
| GmaAffx.89246.1.A1_at | -3.682 | Q8L683 |  | Lectin precursor | protein metabolism |
| Gma.13662.1.S1_at | -3.674 | Q700C7 |  | Similarity to helix-loop-helix DNA-binding protein | transcription |
| GmaAffx.91491.1.S1_s_at | -3.674 | O49875 |  | ADP, ATP carrier protein 1, mitocondrial precursor | transport |
| Gma.7467.1.A1_at | -3.671 | Q9ZPL6 |  | DNA-binding protein 2 (WRKY) | transcription |
| GmaAffx.91200.1.S1_s_at | -3.667 | P35016 |  | Endoplasmin homolog precursor | protein metabolism |
| Gma.17372.3.S1_a_at | -3.661 | Q9XGM1 |  | Mitochondrial ATP synthesis coupled proton transport protein | transport |
| GmaAffx.21782.1.A1_at | -3.659 | Q60D21 |  | Hypothetical Protein | biological process unknown |
| Gma.17368.1.S1_at | -3.659 | Q2PEU0 | * | Serine/threonine kinase | protein metabolism |
| Gma.16111.3.S1_at | -3.658 | O81221 |  | Actin | cell organization and biogenesis |
| Gma.406.5.S1_s_at | -3.658 | P49613 |  | S-adenosylmethionine synthetase | other cellular |
| GmaAffx.4776.1.A1_at | -3.648 | Q9FJY1 |  | Sts14 | biological process unknown |
| GmaAffx.38155.1.S1_at | -3.648 | Q9SAI7 |  | Transducin / WD-40 repeat protein-like | biological process unknown |
| Gma.16367.2.S1_a_at | -3.647 | O80501 |  | GTP-binding protein Rab6 | transport |
| GmaAffx.82443.1.S1_at | -3.647 | Q9FW47 |  | Disease resistance protein | response to abiotic or biotic stimulus |
| AFFX-Gm_P450_3_s_at | -3.646 |  |  | No Hits on UniProt |  |
| Gma.4162.1.S1_at | -3.644 | Q9C9E1 |  | Auxin induced protein | other biological |
| Gma.13796.2.S1_at | -3.643 | Q9FPR6 |  | Two-component response regulator ARR17 | signal transduction |
| GmaAffx.45957.1.S1_at | -3.641 | Q8L924 |  | Integral membrane protein | biological process unknown |
| Gma.10634.1.S1_at | -3.641 | O81872 |  | protease inhibitor | transport |
| GmaAffx.56061.1.S1_at | -3.641 | Q1SNV1 |  | SAM (And some other nucleotide) binding motif | other cellular |
| GmaAffx.23620.1.S1_s_at | -3.637 |  |  | No Hits on UniProt |  |
| Gma.5214.1.S1_at | -3.634 | Q9SUR3 |  | Seed maturation protein | biological process unknown |
| Gma.406.4.S1_at | -3.632 | Q1SFW5 |  | S-adenosylmethionine synthetase | other cellular |
| GmaAffx.92536.1.S1_s_at | -3.627 | Q8W3Y3 |  | 1-aminocyclopropane-1-carboxylic acid oxidase | other cellular |
| GmaAffx.87872.1.S1_at | -3.621 | Q9SY26 |  | CTP synthase | other cellular |
| GmaAffx.51085.1.S1_at | -3.616 | O04840 |  | Nitrite reductase | electron transport or energy pathways |
| Gma.16563.2.S1_a_at | -3.616 | Q9FK79 |  | 26S proteasome subunit-like protein | protein metabolism |
| GmaAffx.89990.1.S1_s_at | -3.612 | Q307Z3 |  | Fructokinase-like | other cellular |
| Gma.15167.1.S1_at | -3.61 | Q1SIC0 |  | Leucoanthocyanidin dioxygenase-like protein |  |
| GmaAffx.12694.1.S1_at | -3.608 | Q8H271 |  | Myb-like transcription factor 1 | transcription |
| Gma.16052.1.S1_at | -3.608 |  | * | No Hits on UniProt |  |
| GmaAffx.92422.1.S1_s_at | -3.603 | Q9SE40 | * | Putative integral membrane protein | other physiological |
| Gma.16718.1.A1_at | -3.602 |  |  | No Hits on UniProt |  |
| Gma.11336.1.S1_at | -3.601 | Q1SSC0 |  | Thaumatin, pathogenesis-related | response to stress |
| GmaAffx.93348.1.S1_s_at | -3.597 | Q1S825 |  | Translation factor; Elongation factor G, III and V | response to stress |
| GmaAffx.6250.1.A1_at | -3.596 | Q9M9F9 |  | Myosin hyeavy chain like protein | biological process unknown |
| GmaAffx.92986.1.S1_at | -3.591 | Q9FQ95 |  | In2-1 protein | biological process unknown |
| GmaAffx.17634.1.S1_at | -3.589 | Q1S6Z1 |  | Ribonuclease T2 | developmental |
| GmaAffx.88242.1.S1_at | -3.589 | Q9SUN5 |  | Putative snRNP protein | other cellular |
| GmaAffx.36627.1.S1_at | -3.586 | Q43207 | * | 70 kDa peptidyl-prolyl isomerase | response to stress |
| Gma.431.1.S1_at | -3.578 | O82134 |  | Proliferating cell nuclear antigen | DNA or RNA metabolism |
| GmaAffx.82528.2.S1_s_at | -3.572 | Q45NI7 |  | Hypothetical Protein | biological process unknown |
| Gma.6081.1.S1_at | -3.569 | Q6JX03 |  | Chitinase-like protein | other cellular |
| GmaAffx.73193.1.S1_at | -3.562 |  |  | No Hits on UniProt |  |
| Gma.15584.2.S1_a_at | -3.561 | Q38JG0 |  | Adenylate kinase family-like protein | electron transport or energy pathways |
| GmaAffx.71230.1.S1_at | -3.561 | Q56WM6 |  | Hypothetical Protein | biological process unknown |
| GmaAffx.42955.1.S1_at | -3.547 | O04865 |  | Phospholipase D alpha 1 | other metabolic |
| GmaAffx.46214.2.S1_s_at | -3.545 | Q7Y249 |  | Polyphenol oxidase (EC 1.10.3.1) |  |
| Gma.4220.2.S1_at | -3.542 | Q8LDW9 |  | Xyloglucan endotransglucosylase/hydrolase protein 9 precursor ( | other metabolic |
| GmaAffx.17904.2.S1_at | -3.535 | Q1SA78 |  | Mini-chromosome maintenance protein MCM3 | DNA or RNA metabolism |
| Gma.7599.1.S1_a_at | -3.535 | Q944G0 | * | Mevalonate disphosphate decarboxylase | other cellular |
| Gma.6613.1.A1_at | -3.526 |  |  | No Hits on UniProt |  |
| Gma.4284.2.S1_s_at | -3.524 | Q1SMR6 |  | Polyphosphoinositide binding protein Ssh2p | transport |
| Gma.1034.4.S1_s_at | -3.522 | Q43095 |  | Caffeoyl-CoA O-methyltransferase |  |
| GmaAffx.71811.1.S1_at | -3.516 | Q67UF5 |  | disulfide-isomerase related protein | electron transport or energy pathways |
| GmaAffx.84607.1.S1_at | -3.511 | Q9FHM9 |  | Phosphate induced protein | biological process unknown |
| Gma.17605.1.S1_at | -3.51 | P24826 |  | Chalcone synthase | other cellular |
| GmaAffx.93348.1.S1_at | -3.508 | Q1S825 |  | Translation factor; Elongation factor G, III and V | response to stress |
| GmaAffx.71901.1.S1_s_at | -3.504 | Q94EN5 |  | Beta-1,3-glucanase | other metabolic |
| Gma.15985.1.S1_at | -3.496 | Q1SZR5 |  | Auxin-induced protein |  |
| GmaAffx.88371.1.S1_at | -3.495 | Q9MBD4 |  | Acyltransferase | developmental |
| GmaAffx.92536.1.S1_at | -3.491 | Q8W3Y3 |  | 1-aminocyclopropane-1-carboxylic acid oxidase | other cellular |
| Gma.2371.1.S1_at | -3.488 | Q2R482 |  | Minichromosomal maintenance factor | DNA or RNA metabolism |
| GmaAffx.64720.1.S1_at | -3.485 | O04450 |  | T-complex protein 1 subunit epsilon | protein metabolism |
| Gma.13186.1.S1_at | -3.483 | Q1SEA5 |  | Inositol oxygenase 4 | other cellular |
| GmaAffx.53477.1.S1_at | -3.474 | Q2PEV4 |  | 60S ribosomal protein L1 | protein metabolism |
| Gma.12692.1.A1_at | -3.471 |  |  | No Hits on UniProt |  |
| Gma.16552.1.A1_s_at | -3.463 |  |  | No Hits on UniProt |  |
| GmaAffx.4919.1.S1_at | -3.462 | Q60D01 |  | Origin recognition complex subunit 6-like protein | DNA or RNA metabolism |
| Gma.5979.1.S1_at | -3.462 | Q8LAB7 |  | Hypothetical Protein | biological process unknown |
| GmaAffx.78026.1.S1_at | -3.461 | Q1T4N5 |  | Peroxiredoxin/thioredocin peroxidase | biological process unknown |
| Gma.12419.1.S1_at | -3.461 | Q2QQS4 |  | Iron-sulfur assembly protein IscA-like 1, mitochondrial precursor | biological process unknown |
| Gma.12928.1.A1_at | -3.46 |  |  | No Hits on UniProt |  |
| GmaAffx.58057.1.S1_at | -3.459 | Q9AS90 |  | Hypothetical Protein | biological process unknown |
| GmaAffx.50239.1.A1_at | -3.454 | Q1S047 | * | Minichromosomal maintenance factor | DNA or RNA metabolism |
| GmaAffx.71287.1.S1_at | -3.454 | Q9C536 |  | Zinc finger protein/ Copia type polyprotein | protein metabolism |
| GmaAffx.74855.1.S1_at | -3.453 | Q1RYA5 |  | Auxin Efflux Carrier Protein | transport |
| Gma.6332.1.S1_at | -3.452 | Q84P23 |  | 4-coumarate-CoA ligase-like protein | response to stress |
| GmaAffx.68649.1.S1_at | -3.451 | Q6K3R8 |  | Chaperonin gamma chain | protein metabolism |
| GmaAffx.85323.1.S1_at | -3.45 | O80786 |  | Minichromosome maintenance deficient protein 5 | DNA or RNA metabolism |
| Gma.2892.2.S1_a_at | -3.45 | Q7X5X9 |  | 26S proteasome subunit RPN6a | protein metabolism |
| GmaAffx.83805.1.S1_at | -3.447 | Q949S3 |  | Hypothetical Protein | biological process unknown |
| Gma.11848.1.S1_at | -3.443 | Q1RX96 |  | Monogalactosyldiacylglycerol synthase | response to stress |
| GmaAffx.88678.1.S1_at | -3.443 | Q9S791 |  | Hypothetical Protein |  |
| GmaAffx.55792.1.S1_at | -3.439 | Q43798 |  | Inorganic pyrophosphatase | transport |
| PsAffx.C62000070_at | -3.439 |  |  | No Hits on UniProt |  |
| GmaAffx.90396.1.S1_s_at | -3.426 | O49875 |  | ADP,ATP carrier protein 1, mitochondrial precursor | transport |
| GmaAffx.88690.1.S1_at | -3.425 | Q7PCB1 |  | Phytosulfokine peptide precursor | other cellular |
| Gma.4300.1.S1_s_at | -3.416 | P30081 |  | Chalcone synthase 7 | other cellular |
| GmaAffx.87379.1.S1_at | -3.406 | O49022 |  | Cytosine-5 DNA methyltransferase | developmental |
| GmaAffx.39393.1.S1_at | -3.406 | P30236 |  | 22.0 kDa class IV heat shock protein precursor | response to stress |
| Gma.6682.1.A1_at | -3.399 | Q6T5H5 |  | Alpha-expansin 3 | developmental |
| GmaAffx.77908.1.S1_at | -3.398 | Q8W465 | * | Hypothetical Protein | biological process unknown |
| GmaAffx.68770.2.S1_at | -3.394 | Q1SPW5 |  | Beta-1,3-glucanase- | other metabolic |
| GmaAffx.83421.2.S1_at | -3.391 | Q1T1K0 | * | Alanyl tRNA synthetase | protein metabolism |
| GmaAffx.93614.1.S1_s_at | -3.391 | Q1RV18 |  | Hypothetical Protein |  |
| Gma.17420.1.S1_at | -3.39 |  |  | No Hits on UniProt |  |
| Gma.14101.1.A1_at | -3.383 |  |  | No Hits on UniProt |  |
| Gma.15947.2.A1_at | -3.38 |  |  | No Hits on UniProt |  |
| Gma.12014.1.A1_at | -3.375 | Q58IU5 |  | ABC transporter 1 | other cellular |
| Gma.2892.2.S1_at | -3.374 | Q7X5X9 |  | Proteasome regulatory particle subunit | protein metabolism |
| Gma.6638.2.S1_a_at | -3.37 | Q75GI1 |  | Symbiotic ammonium transport protein (Putative transcription factor) | transcription |
| GmaAffx.3215.1.A1_at | -3.368 | Q9LY88 |  | Hypothetical Protein | biological process unknown |
| GmaAffx.64309.2.S1_at | -3.366 | Q9LS09 |  | Anti-silencing protein-like | other cellular |
| Gma.11398.4.S1_at | -3.366 | Q7F2H4 |  | ADP-ribosylation factor | protein metabolism |
| GmaAffx.93126.1.S1_s_at | -3.362 | Q1S8N3 |  | Pyruvate kinase, barrel domain | other cellular |
| Gma.10997.1.S1_at | -3.362 | Q9FZ86 |  | Glycosyl hydrolase family 17-.like protein | biological process unknown |
| GmaAffx.59986.1.A1_at | -3.361 |  |  | No Hits on UniProt |  |
| Gma.13864.1.A1_at | -3.358 | Q1SDA6 |  | MYB like DNA binding domain |  |
| Gma.10357.2.S1_at | -3.352 | Q9SQR6 |  | T-complex protein 1, theta subunit | protein metabolism |
| Gma.1382.2.S1_at | -3.352 | O82064 |  | Beta-subunit of K+ channels | transport |
| Gma.11220.3.S1_at | -3.332 | Q1SWD4 |  | AIM1 protein | other cellular |
| Gma.5757.1.S1_at | -3.328 | Q1SCG2 |  | Hypothetical Protein | biological process unknown |
| GmaAffx.90582.1.S1_s_at | -3.326 | Q6VAF9 |  | Alpha-tubulin 1 | cell organization and biogenesis |
| GmaAffx.45780.1.S1_at | -3.321 | Q9FRK6 |  | Hypothetical Protein | biological process unknown |
| GmaAffx.34646.1.S1_at | -3.319 | Q93WZ7 | * | NADH-dependent glutamate synthase | other cellular |
| GmaAffx.83224.1.S1_at | -3.314 | P51851 |  | Pyruvate decarboxylase isozyme | response to stress |
| GmaAffx.85836.1.S1_at | -3.311 | Q9C6M4 |  | Hypothetical Protein | transcription |
| GmaAffx.92715.1.S1_s_at | -3.309 |  |  | No Hits on UniProt | response to abiotic or biotic stimulus |
| GmaAffx.92474.1.S1_s_at | -3.308 | P29516 |  | Tubulin beta | response to stress |
| GmaAffx.45461.1.S1_at | -3.307 | Q7XBI0 |  | SWI2/SNF2-like protein | transcription |
| PsAffx.C101000019_at | -3.306 |  |  | No Hits on UniProt |  |
| Gma.16563.2.S1_x_at | -3.299 | Q9FK79 |  | 26S proteasome subunit-like protein | protein metabolism |
| GmaAffx.86830.1.S1_at | -3.28 | Q1SCG8 |  | Anthocyanin acyltransferase like protein | biological process unknown |
| Gma.13896.1.A1_at | -3.277 |  |  | No Hits on UniProt |  |
| GmaAffx.11155.1.A1_at | -3.276 |  |  | No Hits on UniProt | biological process unknown |
| GmaAffx.65487.1.S1_at | -3.274 | Q38JG4 |  | 26S proteasome regulatory subunit | protein metabolism |
| Gma.2507.2.S1_at | -3.267 | Q651X8 |  | Cellulose synthase-like protein OsCslE1 | other cellular |
| GmaAffx.35147.1.S1_at | -3.267 |  |  | No Hits on UniProt |  |
| GmaAffx.60624.1.S1_at | -3.266 | Q2PEQ3 |  | RAS GTPase activating protein | transport |
| GmaAffx.34595.2.A1_at | -3.265 |  |  | No Hits on UniProt |  |
| GmaAffx.93348.1.S1_x_at | -3.263 | Q1S825 |  | ATP citrate lyase a-subunit | response to stress |
| Gma.9637.1.S1_at | -3.252 | Q7FPX7 |  | Polygalacturonase inhibitor protein | response to abiotic or biotic stimulus |
| GmaAffx.93451.1.S1_s_at | -3.251 | Q2PEW2 |  | Aconitate hydratase, cytoplasmic ( | other metabolic |
| GmaAffx.93550.1.S1_at | -3.247 | Q6TKR0 |  | Ribosomal protein L3 | protein metabolism |
| GmaAffx.83022.1.S1_at | -3.245 | Q84UZ5 |  | Alpha-tubulin | cell organization and biogenesis |
| GmaAffx.77203.1.S1_at | -3.244 | Q8GWT6 |  | Hypothetical Protein | biological process unknown |
| GmaAffx.8550.1.S1_at | -3.238 | Q4VPE6 |  | Phantastica transcription factor a | other cellular |
| Gma.11398.4.S1_a_at | -3.232 | Q7F2H4 |  | ADP-ribosylation factor | protein metabolism |
| GmaAffx.81318.1.S1_at | -3.232 | Q96255 |  | Phosphoserine aminotransferase | other cellular |
| GmaAffx.26732.1.A1_at | -3.226 |  |  | No Hits on UniProt |  |
| Gma.2096.3.S1_s_at | -3.225 | Q9ASX2 |  | Tropinone reductase | other metabolic |
| GmaAffx.87042.2.S1_at | -3.224 | Q9SU40 |  | Monocopper oxidase precursor | developmental |
| GmaAffx.25145.1.A1_at | -3.22 | Q9SML5 |  | Knolle | transport |
| GmaAffx.42464.1.S1_at | -3.22 | Q8LJS7 |  | Homeodomain protein | transcription |
| Gma.16563.4.S1_x_at | -3.216 | Q94E72 |  | 26S proteasome subunit RPN9b | protein metabolism |
| HgAffx.11519.2.S1_at | -3.216 |  |  | No Hits on UniProt |  |
| GmaAffx.92278.1.S1_s_at | -3.214 | O22111 |  | 6-phosphogluconate dehydrogenase | other cellular |
| Gma.56.1.S1_at | -3.214 | Q8H1P3 |  | Phosphoenolpyruvate carboxylase kinase | protein metabolism |
| Gma.2190.1.S1_a_at | -3.201 | O04300 |  | Alpha-1,4-glucan-protein synthase | other cellular |
| GmaAffx.54742.1.S1_at | -3.198 | Q1SD62 |  | DEAD-box ATP-dependent RNA helicase 56 | biological process unknown |
| Gma.12539.1.S1_at | -3.197 | Q6GKX1 |  | Hypothetical Protein | biological process unknown |
| Gma.12483.1.A1_at | -3.188 | Q1SNI6 |  | Sugar transporter superfamily |  |
| Gma.17805.1.A1_s_at | -3.187 | Q1RXM7 |  | Haem peroxidase, plant/fungal/bacterial | response to stress |
| Gma.1917.1.S1_at | -3.177 | Q9FQE8 |  | Glutathione S-transferase | response to stress |
| Gma.2474.2.S1_a_at | -3.173 | Q9SMR7 |  | Hypothetical Protein | biological process unknown |
| Gma.15939.1.S1_at | -3.164 | Q8GW75 |  | Myb related protein | transcription |
| GmaAffx.78740.1.S1_at | -3.164 | Q1SP60 |  | Hypothetical Protein | biological process unknown |
| GmaAffx.84803.1.S1_at | -3.163 | Q1KUM7 |  | Chaperonin | protein metabolism |
| GmaAffx.59784.1.S1_at | -3.162 | Q9SBS1 |  | Ran GTPase activating protein | transport |
| Gma.11109.1.A1_at | -3.157 | Q9SJW9 |  | Hypothetical Protein | DNA or RNA metabolism |
| GmaAffx.75645.1.A1_at | -3.145 | Q1S2D1 |  | UDP-glucose glucosyltransferase | other cellular |
| GmaAffx.90508.1.S1_s_at | -3.145 | Q5ME66 | * | Alpha tubulin 1 | cell organization and biogenesis |
| Gma.1955.3.S1_at | -3.141 | Q67XJ2 |  | Nuclear transcription factor | transcription |
| GmaAffx.56767.1.A1_at | -3.133 | Q9FNI1 | * | Cyclin B like proptein | biological process unknown |
| GmaAffx.66195.1.S1_at | -3.132 | Q1RV75 |  | Lysine decarboxylase-like protein | other cellular |
| GmaAffx.6038.1.A1_at | -3.13 | Q1SI22 |  | NPH3 |  |
| Gma.7778.1.S1_at | -3.126 | O22880 |  | Hypothetical Protein | biological process unknown |
| GmaAffx.59591.1.S1_s_at | -3.124 | O81016 |  | Pleiotropic drug resistance protein | transport |
| Gma.4434.1.S1_at | -3.119 | Q9M510 |  | Dicyanin | electron transport or energy pathways |
| GmaAffx.36979.1.S1_at | -3.117 |  |  | No Hits on UniProt |  |
| Gma.2205.1.S1_at | -3.116 | Q9SWS4 |  | Ripening related protein | biological process unknown |
| GmaAffx.66280.1.S1_at | -3.114 | Q9FJT8 |  | Histone acetyltransferase type B catalytic subunit | biological process unknown |
| Gma.7646.1.A1_at | -3.098 | Q9FHM8 |  | Receptor-like protein kinase | protein metabolism |
| GmaAffx.82721.1.S1_at | -3.093 | Q1RSM6 |  | 26S proteasome subunit P45 | protein metabolism |
| Gma.4218.2.S1_a_at | -3.083 | Q1S275 |  | Hypothetical Protein | biological process unknown |
| GmaAffx.83415.1.S1_at | -3.078 | Q2ABE7 |  | Cyclin D | other cellular |
| GmaAffx.54698.1.S1_at | -3.07 | Q5XV70 |  | Hypothetical Protein | biological process unknown |
| Gma.11296.1.S1_at | -3.063 | Q6KAJ4 |  | Minichromosome maintenance deficient protein 5 | DNA or RNA metabolism |
| Gma.2190.2.S1_x_at | -3.053 | Q9ZR33 |  | Reversibly glycosylated polypeptide | other cellular |
| Gma.16514.2.S1_a_at | -3.038 | Q9AXI4 | * | Hypothetical Protein |  |
| Gma.16524.2.S1_a_at | -3.028 | Q1S125 |  | Single Hybrid Motif |  |
| GmaAffx.88676.1.A1_at | -3.02 |  | * | No Hits on UniProt |  |
| GmaAffx.70690.1.S1_at | -3.017 | P93698 |  | Lipoxygenase LOX1 | response to stress |
| GmaAffx.37109.1.S1_at | -3.015 | Q5NJB1 |  | Receptor protein kinase | protein metabolism |
| Gma.5205.2.S1_at | -3.009 | Q1S7M1 | * | Hypothetical Protein |  |
| GmaAffx.47045.1.S1_at | -3.007 | Q1SVI1 |  | DNA binding protein |  |
| Gma.2507.1.S1_at | -3.004 | Q3Y6V1 |  | Cellulose synthase-like protein CslG | other cellular |
| GmaAffx.86770.1.S1_at | -2.99 | Q8H9D4 |  | 26S proteasome AAA-ATPase subunit RPT4a | protein metabolism |
| Gma.2422.2.S1_at | -2.99 | Q93Y42 | * | Coatomer delta subunit (Delta-coat protein) | transport |
| GmaAffx.91121.1.S1_at | -2.986 | Q6YZD2 |  | Coated vesicle membrane protein-like | transport |
| GmaAffx.54397.2.S1_at | -2.986 | Q948P2 |  | Replication factor, putative | DNA or RNA metabolism |
| Gma.6962.2.S1_at | -2.984 | Q8VYI4 |  | Aspartate-semialdehyde dehydrogenase | other cellular |
| GmaAffx.39814.1.S1_at | -2.981 | Q9FME0 |  | Replication protein A1 | DNA or RNA metabolism |
| GmaAffx.23647.1.S1_at | -2.978 |  |  | No Hits on UniProt |  |
| Gma.2379.1.A1_at | -2.974 | Q2QM08 | * | Hypothetical Protein | biological process unknown |
| GmaAffx.86851.1.S1_at | -2.966 | Q9MAY4 |  | Iron Inhibitted ABC transporter homolog |  |
| GmaAffx.51889.1.S1_at | -2.964 | Q9SUV2 |  | Phosphate/phosphoenolpyruvate translocator protein |  |
| Gma.4707.3.S1_s_at | -2.961 | Q677H6 |  | ADP-ribosylation factor | protein metabolism |
| GmaAffx.54607.1.S1_at | -2.953 | Q94C32 |  | Hypothetical Protein | DNA or RNA metabolism |
| Gma.322.2.S1_at | -2.952 |  |  | No Hits on UniProt | biological process unknown |
| GmaAffx.941.2.S1_s_at | -2.947 | Q9M6T7 | * | Proline-rich protein |  |
| Gma.14851.1.S1_at | -2.941 | Q4ABY1 |  | ATP/GTP binding protein | biological process unknown |
| GmaAffx.80532.2.S1_at | -2.938 | Q69V70 |  | Transmembrane protein/transporter related |  |
| Gma.16674.1.S1_s_at | -2.934 | Q5ME66 | * | Alpha tubulin 1 | cell organization and biogenesis |
| Gma.6493.1.S1_at | -2.932 | Q1SGU9 |  | Hypothetical Protein | biological process unknown |
| Gma.7731.2.S1_at | -2.93 | Q9M9S0 |  | ZF-HD protein dimerisation region | biological process unknown |
| GmaAffx.84030.1.A1_at | -2.93 | Q1SJ61 |  | Zinc finger protein, RING-type;RINGv |  |
| Gma.12279.1.A1_at | -2.924 | Q1T454 |  | basic Helix-loop helix DNA binding protein | transcription |
| Gma.2190.1.S1_x_at | -2.924 | O04300 |  | Alpha-1,4-glucan-protein synthase | other cellular |
| GmaAffx.27899.1.A1_at | -2.917 | Q3LVI1 |  | Hypothetical Protein |  |
| GmaAffx.92555.1.S1_s_at | -2.916 | Q8S2Z7 |  | GTP-binding protein | transport |
| GmaAffx.61002.1.S1_at | -2.912 | O65154 |  | RNA plymerase II transcriptional coactivating protein | transcription |
| GmaAffx.64606.1.S1_at | -2.907 | Q9MBB5 |  | Glucan endo-1-3-beta-glucosidase | other metabolic |
| Gma.2534.3.S1_x_at | -2.902 | Q9S7H2 | * | Ubiquitin | protein metabolism |
| GmaAffx.88134.1.S1_at | -2.888 | Q9FSH5 |  | Cyclin-dependent kinase B | other cellular |
| GmaAffx.73010.1.S1_s_at | -2.883 | Q1SAV1 |  | Pyruvate dehydrogenase E1 component alpha subunit, mitochondrial precursor | other metabolic |
| GmaAffx.36148.1.S1_at | -2.875 | Q9LN83 |  | O-acyl transferase like protein | biological process unknown |
| Gma.8067.1.A1_at | -2.871 | Q1SRY0 |  | Zinc finger-like protein | transcription |
| Gma.2937.1.S1_at | -2.864 |  |  | No Hits on UniProt |  |
| Gma.1394.2.S1_at | -2.863 | Q84LK2 |  | Granule-bound starch synthase Ib precursor | other metabolic |
| Gma.2241.2.S1_at | -2.862 | Q1T2S0 |  | Beta-glucosidase | other metabolic |
| Gma.12894.1.S1_at | -2.859 | Q9S957 |  | Cyclin A homolog | other cellular |
| GmaAffx.80651.1.S1_at | -2.846 | Q9SBS1 |  | Ran GTPase activating protein | other cellular |
| Gma.13695.1.A1_at | -2.844 |  |  | No Hits on UniProt |  |
| HgAffx.134.1.S1_at | -2.842 |  |  | No Hits on UniProt |  |
| GmaAffx.27192.1.S1_at | -2.816 | Q71F77 |  | Thymidine kinase | electron transport or energy pathways |
| GmaAffx.27698.1.S1_at | -2.794 | Q8L924 |  | Integral membrane protein | biological process unknown |
| GmaAffx.45999.1.A1_at | -2.741 |  |  | No Hits on UniProt |  |
| Gma.16913.1.S1_s_at | 3.026 | Q39887 |  | Soybean (G.max) hydroproline-rich protein | cell organization and biogenesis |
| GmaAffx.38484.1.S1_s_at | 3.862 | Q1T2U5 | * | SOUL heme-binding protein; Bacterial regulatory factor, effector | protein metabolism |
| GmaAffx.38484.1.S1_at | 3.888 | Q1T2U5 |  | SOUL heme-binding protein; Bacterial regulatory factor, effector | protein metabolism |
| Gma.1096.1.S1_at | 44.124 | Q9M5X6 |  | Lipid transfer protein precursor | transport |
| Gma.12562.1.A1_at | 63.159 | Q1S8F9 |  | Lipolytic enzyme, G-D-S-L | other biological |
| GmaAffx.63464.1.S1_at | 138.75 |  |  | No Hits on UniProt |  |
| Gma.13296.3.S1_at | 190.34 | Q8H1Z0 |  | Lipid transfer protein; glossy1 homolog | other cellular |
| Gma.1720.1.S1_a_at | 229.624 | Q1SPF8 |  | Hypothetical Protein | protein metabolism |
| GmaAffx.79522.1.A1_s_at | 312.001 |  |  | No Hits on UniProt |  |
| Gma.15677.1.A1_at | 407.404 | Q1S8F9 |  | Lipolytic enzyme, G-D-S-L | other biological |
| GmaAffx.76337.1.S1_at | 519.17 | Q69X62 |  | Beta-ketoacyl-CoA synthase | other metabolic |
| Gma.13367.1.A1_at | 936.68 |  |  | No Hits on UniProt |  |
| GmaAffx.6533.1.A1_at | 2401.068 | Q39048 |  | Fatty acid elongase-like protein (Cer2-like) | other cellular |
| GmaAffx.18376.1.A1_at | 2457.383 | Q677E9 |  | Ubiquitin conjugating enzyme | protein metabolism |
| Gma.17369.1.S1_at | 6232.589 |  |  | No Hits on UniProt |  |
| Gma.17707.1.A1_at | 603126.398 | Q2HVU0 |  | Oxidase/dehydrogenase | other metabolic |
| Gma.12669.1.A1_at | 7984349402 | Q33B71 |  | Lipase, putative | other cellular |
| Gma.12828.1.A1_at | 2.62E+19 | Q1SBU7 |  | Ripening related protein | biological process unknown |
| Gma.16867.1.A1_at | 1.85E+23 |  |  | No Hits on UniProt |  |
| Gma.12584.2.A1_at | 3.48E+25 | Q1SUU0 |  | Acyl-CoA synthetase | other cellular |
| GmaAffx.28196.2.A1_s_at | 5.79E+40 |  |  | No Hits on UniProt |  |
