# Supplementary material for: Integrating microarray analysis and the soybean genome to understand the soybeans iron deficiency response
Source: BMC Genomics. 2009 Aug 13;10:376. doi: 10.1186/1471-2164-10-376 (PMC2907705; doi:10.1186/1471-2164-10-376)
Supplement: Additional file 2 — Differentially expressed transcripts in the IsoClark genotype between plants grown in iron sufficient and iron deficient conditions. A table of differentially expressed genes in the IsoClark genotype comparing plants grown in iron sufficient and iron deficient conditions including the identified fold changes and gene annotations. [file 1471-2164-10-376-S2.doc]

Additional file 2: Differentially Expressed Transcripts in the IsoClark Genotype Between Plants Grown in Iron Sufficient and Iron Deficient Conditions

| Affymetrix Probe ID | Average Fold Change between Iron Sufficient and Iron Deficient | Best Hit UniProt ID | In  QTL | Confirmed Annotation from UniProt | PlantGOSlim |
| --- | --- | --- | --- | --- | --- |
| Gma.12584.2.A1_at | 66.103 | Q1SUU0 |  | Putative acyl-CoA synthetase | other cellular |
| GmaAffx.28196.2.A1_s_at | 57.793 |  |  | No Hits on UniProt |  |
| Gma.16867.1.A1_at | 53.684 |  |  | No Hits on UniProt |  |
| Gma.15718.1.A1_at | 45.976 |  |  | No Hits on UniProt |  |
| Gma.10658.1.A1_at | 42.565 | Q9SWS4 |  | Ripening related protein | biological process unknown |
| Gma.3233.1.S1_s_at | 41.557 | P28759 |  | Iron-superoxide dismutase | response to abiotic or biotic stimulus |
| GmaAffx.81790.1.S1_at | 37.913 |  |  | No Hits on UniProt |  |
| Gma.7557.1.S1_at | 37.627 | Q8H7E2 |  | Ripening related protein | biological process unknown |
| GmaAffx.61521.1.A1_s_at | 36.599 | Q1S8F6 |  | Lipolytic enzyme, G-D-S-L | other biological |
| Gma.17979.1.S1_a_at | 36.119 | Q9M1J5 |  | strictosidine synthase, putative | other metabolic |
| Gma.17724.3.S1_at | 31.571 | Q5J7N0 |  | Putative GDSL-motif lipase/acylhydrolase | other metabolic |
| Gma.12665.1.S1_at | 31.206 | O23414 |  | Hypothetical Protein | biological process unknown |
| Gma.17825.1.A1_at | 29.650 | Q1S8F6 |  | Lipolytic enzyme, G-D-S-L | other biological |
| Gma.16827.1.S1_at | 29.552 | Q71UA1 |  | Iron-superoxide dismutase | response to abiotic or biotic stimulus |
| Gma.34.1.S1_at | 29.403 | Q43461 | * | Nitrate reductase [NADH] 2 (EC 1.7.1.1) (NR-2) | response to abiotic or biotic stimulus |
| GmaAffx.70981.1.S1_at | 28.770 | Q3C1F4 |  | Hemoglobin-2 (Hemoglobin II) | response to stress |
| Gma.17947.1.S1_at | 25.004 | P02519 |  | class I heat shock protein | response to stress |
| Gma.17979.1.S1_x_at | 24.169 | Q9M1J5 |  | strictosidine synthase, putative | other metabolic |
| Gma.1329.1.S1_at | 24.048 | Q8GVQ3 | * | Thiamine biosynthetic enzyme | response to stress |
| GmaAffx.75796.1.S1_at | 23.913 |  |  | No Hits on UniProt |  |
| Gma.13296.3.S1_at | 23.768 | Q8H1Z0 |  | lipid-transfer protein | other cellular |
| Gma.11189.1.S1_at | 23.739 | Q2HTB6 |  | Thylakoid membrane phosphoprotein 14 kDa, chloroplast | other physiological |
| Gma.17724.1.A1_at | 23.730 |  |  | No Hits on UniProt | other metabolic |
| Gma.2480.1.S1_at | 23.542 | Q6EJD0 |  | 1-deoxy-D-xylulose 5-phosphate reductoisomerase | other cellular |
| GmaAffx.63680.1.S1_at | 23.288 | P39866 | * | Nitrate reductase [NADH] 2 (EC 1.7.1.1) (NR-2) | response to abiotic or biotic stimulus |
| Gma.15781.1.A1_at | 22.962 | Q9LR59 |  | F21B7.27 (only hit on UniProt) |  |
| Gma.12665.2.A1_at | 22.438 |  |  | No Hits on UniProt | biological process unknown |
| Gma.18.1.S1_at | 21.259 | Q39819 |  | class IV heat shock protein precursor | response to stress |
| Gma.13367.1.A1_at | 20.814 |  |  | No Hits on UniProt |  |
| Gma.11767.1.A1_a_at | 20.448 | Q2MGQ1 |  | Hypothetical Protein | biological process unknown |
| Gma.13341.1.S1_at | 20.236 | Q94IA1 |  | Trypsin inhibitor A precursor (Kunitz-type trypsin inhibitor A) | biological process unknown |
| Gma.2362.1.S1_at | 19.253 | Q1SPM2 |  | Chlorophyll A-B binding protein | other physiological |
| GmaAffx.76337.1.S1_at | 17.051 | Q69X62 |  | Putative beta-ketoacyl-CoA synthase | other metabolic |
| Gma.13161.1.S1_at | 16.720 | P01070 |  | Trypsin inhibitor A precursor (Kunitz-type trypsin inhibitor A) | biological process unknown |
| Gma.1303.1.S1_at | 16.100 | P39657 |  | putative chitinase |  |
| GmaAffx.71308.1.S1_at | 16.097 | O82042 |  | Heat shock transcription factor (HSFA) (Fragment) |  |
| Gma.17724.2.S1_at | 16.025 |  |  | No Hits on UniProt | other metabolic |
| Gma.10713.2.S1_at | 15.565 | Q41301 |  | Beta-ketoacyl-CoA synthase | other metabolic |
| GmaAffx.89896.1.S1_at | 15.320 | Q2HTU2 |  | class I heat shock protein | response to stress |
| Gma.11162.1.S1_at | 15.317 | Q5QET3 |  | CP12 precursor (Chloroplast protein 12) | biological process unknown |
| Gma.12270.1.S1_at | 15.079 | Q5I2Q5 |  | Benzoyl coenzyme A: benzyl alcohol benzoyl transferase |  |
| Gma.15091.2.S1_at | 14.507 | Q2HTZ6 |  | Hypothetical Protein (only 3 hits on UniProt) | biological process unknown |
| Gma.4340.2.S1_a_at | 13.929 | Q1T3W1 | * | O-methyltransferase, family 2 |  |
| Gma.1061.1.A1_at | 13.684 | Q6K4Z3 |  | Putative esterase | other metabolic |
| GmaAffx.89896.1.S1_s_at | 13.440 | Q2HTU2 |  | class I heat shock protein | response to stress |
| Gma.10980.1.S1_at | 13.287 | Q43681 |  | lipid-transfer protein | transport |
| Gma.13823.2.A1_s_at | 12.928 |  |  | No Hits on UniProt |  |
| Gma.9265.1.S1_at | 12.439 | Q7Y1B9 |  | Ammonium transporter | response to abiotic or biotic stimulus |
| Gma.2446.2.S1_at | 12.361 | Q69NF7 |  | Hypothetical Protein | developmental |
| Gma.10282.1.A1_at | 12.307 | Q1T3Y4 |  | Small heat shock protein | response to stress |
| GmaAffx.43122.1.S1_s_at | 12.267 | Q9M332 | * | Putative Glyoxal oxidase | biological process unknown |
| Gma.13454.1.A1_at | 12.167 | Q8GTM4 |  | Chlorophyllase 1 (EC 3.1.1.14) | other cellular |
| Gma.16866.1.A1_at | 11.913 | O22189 |  | Cytochrome P450 | electron transport or energy pathways |
| Gma.12669.1.A1_at | 11.871 | Q33B71 |  | Lipase, putative | other cellular |
| Gma.764.1.S1_s_at | 11.824 | O23414 |  | Hypothetical Protein | biological process unknown |
| Gma.12852.1.A1_at | 11.692 |  |  | No Hits on UniProt |  |
| Gma.13196.1.A1_at | 11.476 | Q9ST63 |  | Putative internal rotenone-insensitive NADH dehydrogenase | electron transport or energy pathways |
| Gma.17707.1.A1_at | 11.181 | Q2HVU0 | * | putative pod specific dehydrogenase | other metabolic |
| GmaAffx.93268.1.S1_at | 10.969 | P04794 |  | class I heat shock protein | response to stress |
| Gma.13161.2.A1_at | 10.820 | Q94IA1 |  | Trypsin inhibitor A precursor (Kunitz-type trypsin inhibitor A) |  |
| GmaAffx.63464.1.S1_at | 10.643 |  |  | No Hits on UniProt |  |
| Gma.11024.6.S1_s_at | 10.453 | Q1SSD4 |  | Major intrinsic protein | transport |
| Gma.11530.1.A1_at | 10.370 |  |  | No Hits on UniProt |  |
| Gma.12562.1.A1_at | 10.218 | Q1S8F9 |  | Lipolytic enzyme, G-D-S-L | other biological |
| Gma.13800.1.A1_at | 9.844 | Q1SSI3 |  | Hypothetical Protein | biological process unknown |
| Gma.13317.1.A1_at | 9.825 |  |  | No Hits on UniProt |  |
| GmaAffx.69311.1.S1_at | 9.804 | P04794 |  | 17.5 kDa class I heat shock protein (HSP 17.5-E) | response to stress |
| GmaAffx.34657.1.A1_s_at | 9.712 |  | * | No Hits on UniProt |  |
| Gma.12828.1.A1_at | 9.461 | Q1SBU7 |  | Ripening related protein | biological process unknown |
| Gma.7880.1.S1_at | 9.443 | Q1S265 |  | Hypothetical Protein | biological process unknown |
| Gma.2317.1.A1_a_at | 9.370 | Q1SZC4 |  | BURP domain | response to stress |
| Gma.13369.1.S1_at | 9.181 | Q7XA30 |  | S-receptor kinase-like protein 1 | protein metabolism |
| GmaAffx.93268.1.S1_s_at | 9.115 | P04794 |  | class I heat shock protein | response to stress |
| GmaAffx.93221.1.S1_s_at | 9.104 | Q1T3W1 | * | Orcinol O-methyltransferase |  |
| Gma.12688.1.A1_at | 8.981 | Q1SGR4 |  | BHLH transcription factor, | transcription |
| Gma.17712.1.S1_at | 8.947 | Q1T020 |  | Peptidylprolyl isomerase, FKBP-type | protein metabolism |
| Gma.12811.1.A1_at | 8.926 | Q9SGS2 |  | T23E18.4 (only hit on UniProt) | transcription |
| Gma.10713.1.S1_a_at | 8.876 | Q69X62 |  | Beta-ketoacyl-CoA synthase | other metabolic |
| GmaAffx.15792.1.A1_at | 8.808 |  |  |  |  |
| Gma.2522.1.S1_at | 8.758 | P46276 |  | Fructose-1,6-bisphosphatase, cytosolic (EC 3.1.3.11) (D-fructose-1,6-bisphosphate 1-phosphohydrolase) (FBPase) (CY-F1) | other metabolic |
| Gma.2542.1.S1_at | 8.700 | Q9LKH8 |  | Protochlorophyllide reductase, chloroplast precursor (EC 1.3.1.33) (PCR) (NADPH-protochlorophyllide oxidoreductase) (POR) | other cellular |
| Gma.14164.1.A1_at | 8.675 |  |  | No Hits on UniProt |  |
| Gma.14152.1.S1_at | 8.518 | O23758 | * | Nonspecific lipid-transfer protein precursor | transport |
| Gma.1061.2.S1_at | 8.495 | Q1SYC4 |  | Lipolytic enzyme, G-D-S-L | other metabolic |
| GmaAffx.24026.1.S1_at | 8.411 | Q9SSL0 |  | Phosphoglucomutase precursor (EC 5.4.2.2) | other metabolic |
| GmaAffx.19467.1.S1_at | 8.305 | Q8GXZ5 |  | Putative serine/threonine protein kinase | protein metabolism |
| Gma.1221.1.S1_s_at | 8.295 | P54233 |  | Inducible nitrate reductase [NADH] 1 (EC 1.7.1.1) (NR) | response to abiotic or biotic stimulus |
| Gma.4164.3.S1_at | 8.258 | Q1XAN1 | * | Sucrose responsive element binding protein |  |
| Gma.4744.1.S1_x_at | 8.257 |  |  | No Hits on UniProt |  |
| Gma.9036.1.S1_at | 8.148 |  |  | No Hits on UniProt |  |
| Gma.11415.1.A1_at | 8.141 | Q1SAY6 |  | Putative proline-rich protein APG isolog | other metabolic |
| Gma.14152.2.A1_at | 7.991 |  | * | No Hits on UniProt |  |
| GmaAffx.54366.1.S1_at | 7.990 | Q84VI8 |  | Beta-ketoacyl-CoA-synthase | other metabolic |
| Gma.13407.1.A1_at | 7.987 | Q1STK3 |  | Hypothetical Protein |  |
| Gma.16191.1.S1_at | 7.880 | Q7XTZ0 |  | Mandelonitrile lyase, putative | electron transport or energy pathways |
| Gma.8416.1.S1_at | 7.786 | Q9SYR0 |  | Nitrate reductase | response to abiotic or biotic stimulus |
| Gma.4331.2.S1_at | 7.609 | Q1S9L0 |  | lipid-transfer protein | transport |
| Gma.7832.1.S1_at | 7.554 | Q2QCW7 |  | Fatty Acid condensing enzyme CUT1, putative | other cellular |
| Gma.15677.1.A1_at | 7.489 | Q1S8F9 |  | Lipolytic enzyme, G-D-S-L | other biological |
| Gma.3766.1.S1_at | 7.458 | Q1SG42 |  | PsAD2 |  |
| Gma.15490.2.S1_a_at | 7.451 | Q5NE21 |  | Carbonic anhydrase (EC 4.2.1.1) | other metabolic |
| GmaAffx.40837.1.S1_at | 7.434 | Q75W19 |  | Cytochrome P450 | other cellular |
| Gma.17130.1.S1_at | 7.358 | Q1RTA0 |  | Sucrose-phosphate synthase (EC 2.4.1.14) |  |
| Gma.15776.1.A1_at | 7.325 | Q6IDJ6 |  | Hypothetical Protein (only 1 hits on UniProt) | biological process unknown |
| Gma.2516.2.S1_s_at | 7.325 | Q1T4I0 |  | Proline Rich Protein | transport |
| GmaAffx.51832.1.S1_at | 7.172 | Q1S7H7 | * | Hypothetical Protein (only 2 hits on UniProt) | biological process unknown |
| Gma.9647.1.S1_at | 7.100 | Q9C5D7 |  | Caffeoyl-CoA O-methyltransferase (EC 2.1.1.104) (Trans-caffeoyl-CoA 3-O-methyltransferase) (CCoAMT) (CCoAOMT) |  |
| GmaAffx.38010.2.S1_at | 7.043 | Q1S2I7 |  | Plant lipid transfer/seed storage/trypsin-alpha amylase inhibitor | transport |
| Gma.15469.1.S1_at | 6.950 | Q38HT4 |  | Alpha tubulin | cell organization and biogenesis |
| Gma.7477.1.A1_at | 6.875 | Q9SM59 |  | Phosphoglucomutase, chloroplast precursor (EC 5.4.2.2) (Glucose phosphomutase) (PGM) | other cellular |
| GmaAffx.20432.1.S1_at | 6.800 | Q84M38 |  | Hypothetical Protein (only 3 hits on UniProt) | biological process unknown |
| GmaAffx.76027.1.S1_at | 6.758 | Q8L9J7 | * | Putative MtN3 protein | biological process unknown |
| Gma.3272.1.S1_at | 6.740 |  |  | No Hits on UniProt |  |
| GmaAffx.36428.1.A1_at | 6.722 |  |  | No Hits on UniProt |  |
| Gma.10648.1.S1_at | 6.630 | Q9ZPZ4 |  | Hypothetical Protein | biological process unknown |
| Gma.5447.1.S1_at | 6.534 | Q43062 |  | Pectinesterase PPE8B precursor (EC 3.1.1.11) (Pectin methylesterase) (PE) | cell organization and biogenesis |
| Gma.12675.1.A1_at | 6.432 | Q8LB81 |  | Putative GDSL-motif lipase/acylhydrolase | other metabolic |
| Gma.15490.1.S1_a_at | 6.425 | Q84Y09 |  | Carbonic anhydrase 3 (EC 4.2.1.1) | other metabolic |
| GmaAffx.80581.1.S1_at | 6.310 | O04840 |  | Nitrite reductase (EC 1.7.7.1) | electron transport or energy pathways |
| Gma.3310.1.S1_at | 6.285 | Q1RYZ0 |  | putative chaperon |  |
| GmaAffx.32326.1.S1_at | 6.243 | O22817 |  | Putative polygalacturonase (Polygalacturonase/pectinase) | other metabolic |
| GmaAffx.31196.1.S1_s_at | 6.209 | Q9ZTT3 |  | Subtilisin like serine protease | protein metabolism |
| Gma.2773.2.S1_at | 6.184 | Q9SS03 |  | Disease resistance-responsive family protein | response to abiotic or biotic stimulus |
| GmaAffx.40675.1.S1_at | 6.001 | Q94LX1 |  | Chlorophyllase-1, chloroplast precursor (EC 3.1.1.14) (Chlorophyll-chlorophyllido hydrolase 1) (Chlase 1) | other cellular |
| GmaAffx.11213.1.A1_at | 5.896 |  |  | No Hits on UniProt | biological process unknown |
| GmaAffx.25220.1.S1_at | 5.870 | Q2HVU1 |  | putative pod specific dehydrogenase | other metabolic |
| GmaAffx.76826.1.S1_at | 5.866 | Q9STM6 |  | Putative GDSL-like lipase/acylhydrolase | other cellular |
| GmaAffx.73005.1.S1_at | 5.843 |  |  | No Hits on UniProt |  |
| GmaAffx.5924.1.S1_at | 5.827 | Q8L7T2 |  | Small heat shock protein | response to stress |
| GmaAffx.23295.1.S1_at | 5.754 | Q9SRN0 |  | Putative harpin-induced protein |  |
| Gma.7623.1.A1_at | 5.754 | Q6K237 |  | Hypothetical Protein (only 2 hits on UniProt) | other cellular |
| GmaAffx.6533.1.A1_at | 5.726 | Q39048 |  | Fatty acid elongase-like protein (Cer2-like) | other cellular |
| GmaAffx.33640.1.S1_at | 5.338 | Q1SFG3 |  | Hypothetical Protein |  |
| Gma.625.1.S1_at | 5.333 | Q1T1D0 |  | Farnesene synthase | other cellular |
| GmaAffx.56801.1.A1_at | 5.318 | Q1SMU6 |  | Hypothetical Protein | biological process unknown |
| Gma.1174.1.S1_at | 5.290 | Q84MP5 |  | Hypothetical Protein (only 3 hits on UniProt) |  |
| GmaAffx.73904.1.S1_at | 5.256 | Q94ET0 | * | Hydroxymethylglutaryl coenzyme A synthase | other cellular |
| Gma.17.1.S1_at | 5.243 | Q39820 |  | class IV heat shock protein precursor | response to stress |
| Gma.14094.1.A1_at | 5.085 |  |  | No Hits on UniProt |  |
| Gma.8333.1.S1_at | 5.038 | O24292 |  | CP12 precursor | biological process unknown |
| GmaAffx.12145.1.S1_at | 4.928 | Q8LAP4 |  | Myb transcription factor | response to stress |
| Gma.13789.1.A1_at | 4.898 | Q1T048 |  | Peptidase M14, carboxypeptidase A | biological process unknown |
| Gma.3723.1.S1_at | 4.847 | Q43681 |  | nonspecific lipid-transfer protein | transport |
| GmaAffx.76187.1.S1_at | 4.843 | Q8LP23 |  | UDP Glucosyltransferase | other metabolic |
| Gma.1102.1.S1_at | 4.839 | Q5N8N4 |  | Hypothetical Protein (only 3 hits on UniProt) | biological process unknown |
| Gma.14748.1.S1_s_at | 4.770 | Q2PHF4 |  | Aldehyde oxidase 2 (EC 1.2.3.1) (AtAO-2) (AtAO3) | other cellular |
| GmaAffx.6785.1.A1_at | 4.712 |  |  | No Hits on UniProt |  |
| Gma.10581.1.S1_at | 4.703 | Q8GUC2 |  | Putative phosphatase | other metabolic |
| GmaAffx.61184.1.S1_at | 4.672 | Q6J540 |  | Cytochrome P450 | electron transport or energy pathways |
| GmaAffx.49404.1.S1_at | 4.634 | Q8GT20 |  | Benzoyl coenzyme A | biological process unknown |
| GmaAffx.86212.1.S1_at | 4.557 | O65709 |  | Similarity to long chain fatty alcohol oxidase (At3g23410/MLM24_23) | electron transport or energy pathways |
| Gma.11944.1.S1_at | 4.523 | Q7XWZ6 |  | Flavin containing monooxygenase 3 like protein | electron transport or energy pathways |
| Gma.1281.2.S1_at | 4.519 | O64866 |  | putative calcium binding EF hand family protein | biological process unknown |
| GmaAffx.4270.1.S1_s_at | 4.434 |  |  | No Hits on UniProt |  |
| Gma.2252.1.S1_at | 4.429 | Q8GW58 |  | Hypothetical Protein | biological process unknown |
| Gma.768.1.S1_at | 4.419 | Q6NLE8 |  | Hypothetical Protein | biological process unknown |
| Gma.2554.1.S1_at | 4.410 | Q652J5 |  | Alpha/beta hydrdolase fold family | response to stress |
| Gma.2574.1.S1_at | 4.409 | Q8LPT9 |  | Alpha-glucan water dikinase, chloroplast precursor (EC 2.7.9.4) (Starch-related R1 protein) | response to stress |
| Gma.2062.2.S1_a_at | 4.374 | Q1RV82 |  | Hypothetical Protein (only 3 hits on UniProt) | biological process unknown |
| Gma.5382.1.S1_at | 4.374 | Q9SXE6 |  | Extensin like protein | transport |
| GmaAffx.77637.1.S1_at | 4.305 | Q2ENC4 |  | Chalcone synthase | other cellular |
| GmaAffx.48040.1.A1_at | 4.273 |  |  | No Hits on UniProt |  |
| Gma.3233.1.S1_at | 4.251 | P28759 |  | Iron-superoxide dismutase | response to abiotic or biotic stimulus |
| GmaAffx.5738.1.S1_at | 4.247 | Q1SMF6 |  | Esterase/lipase/thioesterase | biological process unknown |
| GmaAffx.20412.1.A1_at | 4.230 |  |  |  |  |
| Gma.17341.1.S1_at | 4.206 | Q2HVI0 | * | Hypothetical Protein | biological process unknown |
| GmaAffx.9536.1.S1_at | 4.201 | Q2HUY0 |  | Hypothetical Protein | biological process unknown |
| GmaAffx.27496.2.S1_at | 4.189 | Q7X9G5 |  | Lipoxygenase (EC 1.13.11.12) | electron transport or energy pathways |
| Gma.13004.1.S1_at | 4.174 | Q5ZF88 |  | Hypothetical Protein | biological process unknown |
| Gma.6552.1.S1_at | 4.158 | Q1SJX4 |  | Natural resistance-associated macrophage protein ( Root-specific metal transporter) | transport |
| GmaAffx.78009.1.S1_at | 4.120 | P81392 |  | MYB-related protein 306 | response to stress |
| Gma.16947.1.S1_at | 4.117 |  |  | No Hits on UniProt | cell organization and biogenesis |
| Gma.13140.4.S1_at | 4.099 | Q1SCN9 |  | Aldehyde dehydrogenase (NAD) family protein | other metabolic |
| Gma.13613.1.A1_at | 4.093 |  |  | No Hits on UniProt |  |
| Gma.5173.2.S1_at | 4.087 |  |  | No Hits on UniProt |  |
| GmaAffx.79522.1.A1_s_at | 4.068 |  |  | No Hits on UniProt |  |
| Gma.13140.1.A1_at | 4.061 | Q1SCN9 |  | Aldehyde dehydrogenase (NAD) family protein | other metabolic |
| Gma.10095.1.A1_at | 4.031 | Q1SZ14 |  | NADPH HC toxin reductase | other cellular |
| GmaAffx.6001.1.S1_at | 4.027 |  |  | No Hits on UniProt | cell organization and biogenesis |
| Gma.832.1.S1_a_at | 3.997 | Q2HVR6 |  | RNA-binding region RNP-1 (RNA recognition motif) | biological process unknown |
| Gma.13186.1.S1_at | 3.991 | Q1SEA5 |  | Probable inositol oxygenase (EC 1.13.99.1) (Myo-inositol oxygenase) | other cellular |
| Gma.48.1.S1_at | 3.964 | Q39889 |  | Heat shock protein 101 | response to stress |
| Gma.3384.1.S1_at | 3.954 | Q5QIV3 | * | Acyl CoA Reductase | developmental |
| Gma.1937.1.S1_at | 3.941 | Q26YQ4 | * | Hypothetical Protein (only 2 hits on UniProt) |  |
| GmaAffx.41460.1.S1_at | 3.939 |  |  | No Hits on UniProt |  |
| Gma.79.1.S1_s_at | 3.924 | Q39887 |  | Proline-rich protein | cell organization and biogenesis |
| GmaAffx.53903.1.A1_at | 3.923 |  |  | No Hits on UniProt |  |
| Gma.14039.1.A1_at | 3.911 |  |  | No Hits on UniProt |  |
| Gma.10104.1.S1_at | 3.892 |  |  | No Hits on UniProt | response to stress |
| GmaAffx.37697.1.S1_at | 3.858 | Q1ST72 |  | GPI anchored protein | biological process unknown |
| GmaAffx.20374.2.S1_at | 3.848 | Q5ZF88 |  | Hypothetical Protein | biological process unknown |
| GmaAffx.11155.1.A1_at | 3.806 |  |  | No Hits on UniProt | biological process unknown |
| GmaAffx.82556.1.S1_at | 3.804 | Q67WQ7 |  | Putative Glossy1 Protein | other cellular |
| GmaAffx.30428.1.S1_at | 3.753 | P26413 |  | Heat shock 70 kDa protein | response to stress |
| GmaAffx.87934.1.S1_at | 3.742 | Q1T1D0 |  | Alpha-farnesene synthase | other cellular |
| Gma.1294.1.S1_at | 3.733 | Q8VZU6 |  | Hypothetical Protein | biological process unknown |
| GmaAffx.65393.1.S1_s_at | 3.709 |  |  | No Hits on UniProt |  |
| Gma.12832.1.A1_at | 3.591 | Q8VZW5 |  | Dynein light chain protein | cell organization and biogenesis |
| Gma.13190.1.A1_at | 3.567 | P54240 |  | Glucose-6-phosphate isomerase, cytosolic 1 (EC 5.3.1.9) (GPI) (Phosphoglucose isomerase) (PGI) (Phosphohexose isomerase) (PHI) | other cellular |
| GmaAffx.48977.1.S1_at | 3.550 | Q9C8T5 |  | Hypothetical Protein | biological process unknown |
| GmaAffx.13179.1.S1_at | 3.429 | Q1RYE3 |  | Myosin heavy chain like | biological process unknown |
| Gma.13204.2.A1_at | 3.411 | Q1SNE3 |  | Plasma membrane associated protein, putative | biological process unknown |
| Gma.6613.1.A1_at | 3.386 |  |  | No Hits on UniProt |  |
| Gma.10447.2.A1_a_at | 3.304 | Q1T635 |  | MATE efflux protein | transport |
| GmaAffx.3256.2.S1_at | 3.299 | Q8LP22 |  | Flavonol synthase/flavanone 3-hydroxylase (EC 1.14.11.23) (EC 1.14.11.9) (FLS) | other cellular |
| Gma.4512.2.S1_at | 3.291 | O04341 |  | Putative glutaredoxin (At2g30540/T6B20.11) | electron transport or energy pathways |
| Gma.12966.1.S1_at | 3.274 | Q67ZI9 |  | Putative APG protein | other metabolic |
| Gma.6477.1.S1_at | 3.259 | Q9XFI8 |  | Peroxidase | response to stress |
| Gma.7010.1.S1_at | 3.244 | P53537 |  | Alpha-glucan phosphorylase, H isozyme (EC 2.4.1.1) (Starch phosphorylase H) | other metabolic |
| GmaAffx.30771.1.S1_at | 3.210 | Q9FNI1 |  | Cyclin B like Protein | biological process unknown |
| GmaAffx.71692.1.S1_at | 3.145 | Q1STD8 |  | UDP-glucuronosyl/UDP-glucosyltransferase | response to stress |
| Gma.2574.1.S1_a_at | 3.136 | Q8LPT9 |  | Alpha-glucan water dikinase, chloroplast precursor (EC 2.7.9.4) (Starch-related R1 protein) | response to stress |
| GmaAffx.28861.1.S1_at | 3.100 | Q1S0Z4 |  | Putative subtilisin serine protease | protein metabolism |
| Gma.9838.1.S1_at | 3.095 | Q9AR82 |  | Thioredoxin H-type 1 (TRX-H-1) | electron transport or energy pathways |
| GmaAffx.80064.1.S1_at | 2.933 | Q93YH3 |  | ATP citrate lyase b-subunit (EC 4.1.3.8) | other cellular |
| Gma.4457.1.S1_a_at | 2.925 | Q9LZJ5 |  | Multidrug resistance-associated protein 10 (EC 3.6.3.44) (Glutathione S-conjugate transporting ATPase 10) (ATP-energized glutathione S-conjugate pump 10) | transport |
| GmaAffx.20374.1.A1_at | 2.890 |  |  | No Hits on UniProt |  |
| GmaAffx.45393.1.S1_at | 2.858 | Q1RXH4 |  | UDP-glucuronosyl/UDP-glucosyltransferase | other metabolic |
| Gma.79.1.S1_x_at | 2.857 | Q39887 |  | Proline-rich protein | cell organization and biogenesis |
| GmaAffx.89697.1.S1_s_at | 2.736 | O23961 |  | Peroxidase | response to stress |
| GmaAffx.28120.1.S1_at | 2.623 | Q9ZWS2 |  | UDP-galactose:flavonol 3-O-galactosyltransferase | other metabolic |
| Gma.15538.1.S1_at | 2.592 | Q8L8Z8 |  | Glutaredoxin-like protein | electron transport or energy pathways |
| Gma.10073.2.S1_at | 2.481 | Q6Y0F0 | * | GDSL-motif lipase (Fragment) | other metabolic |
| GmaAffx.89407.1.A1_s_at | 2.446 | Q1KL62 |  | Lipid transfer protein-like protein | transport |
| GmaAffx.87400.1.S1_at | 2.262 | Q94CH6 | * | Lipolytic enzyme, G-D-S-L | other biological |
| Gma.3539.2.S1_at | 2.242 | Q9STY1 |  | Glycerol 3 phosphate transporter | transport |
| Gma.1008.1.A1_at | 2.231 | Q8RY68 |  | IDS4 like protein | biological process unknown |
| GmaAffx.88690.1.S1_at | 2.196 | Q7PCB1 |  | Putative phytosulfokine peptide precursor | other cellular |
| GmaAffx.58559.1.A1_at | 2.166 | Q1S6U2 |  | Anthranilate N-benzoyltransferase protein 1 (EC 2.3.1.144) (Anthranilate N-hydroxycinnamoyl/benzoyltransferase 1) | protein metabolism |
| GmaAffx.90865.1.S1_s_at | 2.157 | Q39817 |  | Calnexin homolog precursor |  |
| GmaAffx.83079.1.S1_at | 2.134 |  |  | No Hits on UniProt |  |
| Gma.3881.2.S1_at | 2.081 | P37115 | * | Trans-cinnamate 4-monooxygenase (EC 1.14.13.11) (Cinnamic acid 4-hydroxylase) (CA4H) (C4H) (P450C4H) (Cytochrome P450 73) | response to abiotic or biotic stimulus |
| GmaAffx.23059.1.S1_at | 2.071 | Q9ZWS2 |  | Flavonoid 3-O-galactosyl transferase | other metabolic |
| GmaAffx.90703.1.A1_at | 2.021 | Q7XYR7 |  | Class III peroxidase | response to stress |
| GmaAffx.68386.1.S1_at | 3.410 |  |  | No Hits on UniProt | biological process unknown |
| Gma.2342.1.S1_at | 3.524 | Q43457 |  | Heat shock transcription factor | response to stress |
| Gma.612.1.A1_at | 4.036 | Q6SA75 |  | putative transcription factor (only 2 hits on uniprot) |  |
| GmaAffx.23237.1.S1_at | 4.183 | Q94XE1 |  | Cytochrome oxidase subunit I | electron transport or energy pathways |
| GmaAffx.90642.1.S1_s_at | 4.277 | Q2L989 |  | Putative DnaJ protein | protein metabolism |
| GmaAffx.14166.1.S1_at | 4.834 | P11995 |  | Larval serum protein 1 alpha chain precursor (Hexamerin 1 alpha) |  |
| Gma.10216.3.A1_x_at | 5.284 | Q2HTB5 |  | O-methyltransferase, family 2 | other cellular |
| GmaAffx.30562.1.S1_at | 5.815 |  |  | No Hits on UniProt |  |
| Gma.6211.1.S1_at | 8.876 | Q9FLM1 |  | glycerophosphoryl diester phosphodiesterase | other cellular |
| Gma.17733.1.S1_s_at | 9.063 | Q1S278 |  | Proteinase inhibitor | response to stress |
| Gma.6211.2.A1_at | 11.771 | Q84WI7 |  | glycerophosphoryl diester phosphodiesterase | other cellular |
| GmaAffx.51208.1.S1_at | 14.838 | Q8HQ04 |  | NADH dehydrogenase subunit 1 | other physiological |
| Gma.7769.1.A1_at | 17.020 | Q6H5Z0 | * | Hypothetical Protein |  |
| GmaAffx.78368.1.S1_at | 20.263 | Q94XD8 |  | Cytochrome oxidase subunit III | electron transport or energy pathways |
| Gma.197.1.S1_at | 31.956 | Q42792 |  | Asparagine synthetase (EC 6.3.5.4) | other cellular |
| GmaAffx.8505.1.A1_s_at | 117.272 | P42791 |  | Cytoplasmic ribosomal protein L18 | protein metabolism |
