# Supplementary material for: Integrating microarray analysis and the soybean genome to understand the soybeans iron deficiency response
Source: BMC Genomics. 2009 Aug 13;10:376. doi: 10.1186/1471-2164-10-376 (PMC2907705; doi:10.1186/1471-2164-10-376)
Supplement: Additional file 3 — Differentially Expressed Genes between Clark and IsoClark genotypes grown under Iron Deficient Conditions. A table of differentially expressed genes between Clark and IsoClark genotypes grown under iron deficient conditions including the identified fold changes and gene annotations. [file 1471-2164-10-376-S3.doc]

Additional file 3: Differentially Expressed Genes between Clark and IsoClark Genotypes Grown Under Iron Deficient Conditions

| Affymetrix Probe ID | Fold Change Between Clark and IsoClark | UniProt ID | UniProt Annotation | PlantGOSlim |
| --- | --- | --- | --- | --- |
| GmaAffx.28196.2.A1_s_at | -111.83 | Q6K4D2 | Putative ABC transporter |  |
| Gma.12584.2.A1_at | -67.97 | Q56WP3 | Putative Acetyl Co A Synthetase | Other Metabolic Processes |
| Gma.16867.1.A1_at | -49.82 |  | No UniProt ID |  |
| Gma.13367.1.A1_at | -38.99 |  | No UniProt ID |  |
| Gma.15718.1.A1_at | -36.70 |  | No UniProt ID |  |
| GmaAffx.81790.1.S1_at | -32.85 | Q9FFD2 | Amylogenin |  |
| Gma.12828.1.A1_at | -32.59 | Q1SBU7 | Ripening Related Protein | Unknown Biological Process |
| Gma.12669.1.A1_at | -31.50 | Q33B71 | Lipase | Other Metabolic Processes |
| GmaAffx.76337.1.S1_at | -26.78 | Q1SHA0 | DNA-directed DNA polymerase | Other Metabolic Processes |
| GmaAffx.63464.1.S1_at | -26.51 |  | No UniProt ID |  |
| GmaAffx.62046.1.S1_at | -25.91 | Q4ZJ73 | 12-oxophytodienoate reductase | Developmental Processes |
| Gma.13296.3.S1_at | -19.37 | Q9FJN4 | Lipid transfer protein | Other Metabolic Processes |
| Gma.17707.1.A1_at | -18.91 | Q8LCE7 | Pod Specific Dehydrogenase | Other Metabolic Processes |
| Gma.34.1.S1_at | -16.94 |  | No UniProt ID | Response to Abiotic Stimuli |
| Gma.10713.2.S1_at | -14.06 | Q41301 | Beta-ketoacyl-CoA synthase | Other Metabolic Processes |
| Gma.13341.1.S1_at | -13.97 | Q94IA1 | Kunitz-type trypsin inhibitor A | Unknown Biological Process |
| Gma.12562.1.A1_at | -12.63 | Q1S8F9 | Lipolytic enzyme, G-D-S-L | Developmental Processes |
| Gma.1061.1.A1_at | -11.28 | Q9LHX8 | Esterase | Other Metabolic Processes |
| Gma.13196.1.A1_at | -11.26 | Q9ST63 | Internal rotenone-insensitive NADH dehydrogenase | Electron Transport |
| GmaAffx.6533.1.A1_at | -11.15 | Q6A1I2 | Cathepsin B | Other Metabolic Processes |
| Gma.13161.2.A1_at | -10.98 | Q94IA1 | Kunitz-type trypsin inhibitor A |  |
| GmaAffx.34657.1.A1_s_at | -10.51 |  | No UniProt ID |  |
| Gma.15677.1.A1_at | -10.46 | Q1S8F9 | Lipolytic enzyme, G-D-S-L | Developmental Processes |
| Gma.12852.1.A1_at | -9.95 |  | No UniProt ID |  |
| Gma.2516.2.S1_s_at | -9.74 | Q2R482 | Minichromosomal maintenance factor | Transport |
| Gma.1329.1.S1_at | -9.60 | Q8GVQ3 | Thiamine biosynthetic enzyme | Response to stress |
| Gma.7832.1.S1_s_at | -8.93 | O04946 | Enoyl-ACP reductase | Other Metabolic Processes |
| Gma.13161.1.S1_at | -8.74 | P01070 | Kunitz-type trypsin inhibitor | Unknown Biological Process |
| Gma.11767.1.A1_a_at | -8.65 | Q2MGQ1 | Hypothetical | Unknown Biological Process |
| Gma.4331.2.S1_at | -8.55 | O82134 | Proliferating cell nuclear antigen | Transport |
| Gma.13454.1.A1_at | -7.87 | Q8GTM4 | Chlorophyllase 1 | Response to Stress |
| GmaAffx.75796.1.S1_at | -7.80 | Q4LAW1 | Replication factor A |  |
| Gma.14161.1.A1_at | -7.76 |  | No UniProt ID |  |
| Gma.13317.1.A1_at | -7.70 |  | No UniProt ID |  |
| GmaAffx.19467.1.S1_at | -7.59 |  | No UniProt ID | Protein Metabolism |
| Gma.3233.1.S1_at | -7.31 | Q9FX43 | MAP kinase | Response to Abiotic Stimuli |
| Gma.15091.2.S1_at | -7.05 | Q2HTZ6 | Hypothetical | Unknown Biological Process |
| GmaAffx.89671.1.A1_at | -7.04 | P15490 | Vegetative Storage Protein |  |
| Gma.9265.1.S1_at | -7.02 | Q7M1S6 | Trypsin inhibitor | Response to Biotic Stimuli |
| Gma.5447.1.S1_at | -6.85 |  | No UniProt ID | Cell Organization |
| GmaAffx.38010.2.S1_at | -6.79 | Q6DU55 | S-domain receptor kinase | Transport |
| Gma.7832.1.S1_x_at | -6.74 | Q9XF43 | Fatty Acid Condensing Enzyme | Other Metabolic Processes |
| Gma.12811.1.A1_at | -6.74 | Q9SGS2 | T23E18.4 | Transcription |
| GmaAffx.54366.1.S1_at | -6.51 | Q9FYG6 | Hypothetical | Other Metabolic Processes |
| Gma.2522.1.S1_at | -6.50 | Q41125 | Proline-rich protein | Other Metabolic Processes |
| Gma.11415.1.A1_at | -6.36 | Q9FT04 | Proline-rich protein | Other Metabolic Processes |
| GmaAffx.79522.1.A1_s_at | -6.16 | Q94XD8 | Cytochrome oxidase |  |
| Gma.3705.1.S1_at | -6.06 | Q43461 | Nitrate reductase | Other Metabolic Processes |
| Gma.7407.1.S1_at | -5.78 | Q7XAB7 | Cyclin D3-2 | Developmental Processes |
| GmaAffx.62877.1.A1_at | -5.67 |  | No UniProt ID |  |
| Gma.2040.1.A1_at | -5.42 | Q9FQE8 | Glutathione S-transferase | Unknown Biological Process |
| GmaAffx.43343.2.S1_s_at | -5.31 |  | No UniProt ID |  |
| GmaAffx.12196.1.S1_at | -5.28 |  | No UniProt ID |  |
| Gma.2213.1.S1_at | -5.08 | Q7F1F2 | 70 kDa peptidylprolyl isomerase | Other Metabolic Processes |
| Gma.4744.1.S1_x_at | -5.05 |  | No UniProt ID |  |
| Gma.3233.1.S1_s_at | -4.99 | P28759 | Iron-superoxide dismutase | Response to Abiotic Stimuli |
| Gma.13375.1.A1_s_at | -4.86 | Q8LCJ5 | DnaJ protein | Protein Metabolism |
| Gma.13789.1.A1_at | -4.52 | Q1S5R9 | Peptidase M14 | Unknown Biological Process |
| Gma.3272.1.S1_at | -4.47 | P28759 | Iron-superoxide dismutase |  |
| Gma.6552.1.S1_at | -4.35 | Q9ZT91 | Translational elongation factor Tu | Transport |
| GmaAffx.20412.1.A1_at | -4.22 |  | No UniProt ID |  |
| GmaAffx.48202.1.S1_at | -4.19 | Q41122 | Proline-rich protein |  |
| Gma.15490.1.S1_a_at | -4.18 | Q84Y09 | Carbonic anhydrase | Other Metabolic Processes |
| Gma.8185.1.A1_at | -4.04 | Q67XP0 | Zinc finger-like protein |  |
| GmaAffx.20469.1.S1_at | -3.88 |  | No UniProt ID | Energy Pathways |
| GmaAffx.85164.1.S1_at | -3.53 | Q6ZDW5 | Cytoplasmic ribosomal protein | Other Metabolic Processes |
| Gma.10151.1.S1_at | -3.53 |  | No UniProt ID |  |
| GmaAffx.72.1.S1_at | -3.51 | Q9LUE7 | ATFP3 | Other Metabolic Processes |
| Gma.13004.1.S1_at | -3.50 | Q5ZF88 | Hypothetical | Unknown Biological Process |
| GmaAffx.32606.1.S1_at | -3.29 | Q8VZT0 | Ribonucleoprotein complex | Transport |
| GmaAffx.5738.1.S1_at | -3.27 | Q9FNI1 | Cyclin B-type protein | Unknown Biological Process |
| Gma.3766.1.S1_at | -3.26 | Q39734 | Phosphate dikinase |  |
| GmaAffx.48977.1.S1_at | -3.07 | Q9CA59 | Nucleosome | Unknown Biological Process |
| GmaAffx.20374.1.A1_at | -2.87 | Q8RXC8 | Serine/threonine protein kinase |  |
| Gma.2569.1.S1_s_at | -2.87 | P46276 | Fructose-1,6-bisphosphatase |  |
| Gma.12660.1.A1_at | -2.85 | Q8H2B1 | DnaJ-like | Protein Metabolism |
| Gma.15925.1.S1_at | -2.63 | Q9SW05 | Pathogenesis-related | Unknown Biological Process |
| GmaAffx.49167.1.S1_at | -2.61 | Q9C8T5 | Hypothetical | Response to Abiotic Stimuli |
| GmaAffx.58990.1.S1_at | -2.49 | Q9ZWQ4 | Glucosyltransferase | Other Metabolic Processes |
| Gma.12731.1.S1_at | -2.26 | Q9FK43 | Aquaporin SIP1.2 | Transport |
| Gma.7766.1.S1_at | -2.26 |  | No UniProt ID | Response to Abiotic Stimuli |
| Gma.15538.1.S1_at | -2.26 | Q8L8Z8 | Glutaredoxin | Electron Transport |
| GmaAffx.25010.1.S1_at | -2.11 | Q94XE1 | Cytochrome oxidase | Other Metabolic Processes |
| GmaAffx.65393.1.S1_s_at | -2.06 | Q39048 | Cer2 |  |
| GmaAffx.90913.1.S1_s_at | -2.02 | Q5QLR2 | Glutaredoxin-like | Response to Abiotic Stimuli |
| GmaAffx.72251.1.S1_at | 2.01 |  | No UniProt ID | DNA Metabolism |
| Gma.17839.1.S1_at | 2.01 | Q9FME9 | Hypothetical | Unknown Biological Process |
| GmaAffx.90001.1.S1_at | 2.04 | Q8W2E3 | 3-hydroxy-3-methylglutaryl coenzyme A | Other Metabolic Processes |
| GmaAffx.18773.1.S1_at | 2.06 | Q1SA78 | Mini-chromosome maintenance | RNA Metabolism |
| GmaAffx.766.1.S1_at | 2.07 | Q93W30 | Hypothetical | Unknown Biological Process |
| Gma.18014.1.S1_a_at | 2.07 | Q6H525 | Protodermal Factor |  |
| GmaAffx.10162.1.S1_at | 2.12 | Q41350 | Osmotin | Unknown Biological Process |
| Gma.1614.1.S1_at | 2.12 | Q84J37 | Laccase | Other Metabolic Processes |
| GmaAffx.13543.1.A1_at | 2.13 |  | No UniProt ID | Other Metabolic Processes |
| GmaAffx.87089.1.S1_at | 2.14 |  | No UniProt ID | Unknown Biological Process |
| Gma.1379.3.S1_at | 2.16 | Q1S753 | Gibberellin Regulated Protein | Other Biological Processes |
| GmaAffx.25551.1.S1_at | 2.20 | Q2HZ19 | Polygalacturonase | Unknown Biological Process |
| GmaAffx.87379.1.S1_at | 2.21 |  | No UniProt ID | Developmental Processes |
| GmaAffx.17904.2.S1_at | 2.22 | Q1SA78 | Mini-chromosome maintenance | DNA Metabolism |
| GmaAffx.64502.1.S1_at | 2.22 |  | No UniProt ID |  |
| Gma.1439.1.S1_at | 2.24 | Q07185 | Alternative oxidase | Electron Transport |
| Gma.17174.1.S1_s_at | 2.29 | Q75GV2 | Hypothetical | Unknown Biological Process |
| Gma.15947.1.S1_at | 2.31 | Q6Z1Z2 | HMG type nucleosome | Transcription |
| Gma.4630.1.S1_at | 2.33 | Q1SG45 | Hypothetical | Transport |
| Gma.18014.2.S1_x_at | 2.36 | Q6H525 | Protodermal |  |
| GmaAffx.37281.1.S1_at | 2.37 | Q6NLG7 | Hypothetical | DNA Metabolism |
| Gma.15048.2.S1_at | 2.39 | Q3S345 | Zinc finger | Unknown Biological Process |
| Gma.5496.1.S1_s_at | 2.39 |  | No UniProt ID | Other Metabolic Processes |
| GmaAffx.27527.1.S1_at | 2.42 | Q9S750 | Thymidine kinase |  |
| Gma.15610.1.S1_at | 2.75 | Q1T055 | MLP Like Protein | Unknown Biological Process |
| GmaAffx.72063.1.S1_at | 2.77 | Q1SDJ4 | G-D-S-L Lipolytic enzyme |  |
| Gma.3026.1.S1_at | 2.89 |  | No UniProt ID |  |
| GmaAffx.51208.1.S1_at | 3.02 | Q1S047 | Mini-chromosome maintenance | Other Biological Processes |
| Gma.5465.2.S1_at | 3.12 | Q43062 | Pectinesterase |  |
| Gma.11362.1.S1_at | 3.22 | Q9ST53 | MADS-box | Response to Abiotic Stimuli |
| Gma.1704.1.S1_at | 3.31 | Q9SGP6 | Glutaredoxin | Electron Transport |
| GmaAffx.40775.1.S1_at | 3.35 | Q9FME0 | Replication protein A1 |  |
| Gma.9839.2.S1_at | 3.37 | Q41350 | Thaumatin | Response to Biotic Stimuli |
| Gma.9202.1.S1_at | 3.41 |  | No UniProt ID | Unknown Biological Process |
| Gma.431.2.S1_x_at | 3.42 | O82134 | Proliferating cell nuclear antigen | DNA Metabolism |
| Gma.431.2.S1_a_at | 3.45 | O82134 | Proliferating cell nuclear antigen | DNA Metabolism |
| GmaAffx.87101.1.S1_at | 3.49 | Q60D21 | Hypothetical |  |
| Gma.17489.1.S1_at | 3.88 |  | No UniProt ID |  |
| Gma.12326.1.S1_at | 3.90 |  | No UniProt ID |  |
| Gma.5350.1.S1_s_at | 3.93 |  | No UniProt ID | Transport |
| Gma.4295.1.S1_s_at | 3.94 | Q9XFI8 | Peroxidase | Other Metabolic Processes |
| GmaAffx.93132.1.S1_at | 3.98 |  | No UniProt ID | Response to Abiotic Stimuli |
| GmaAffx.81059.1.S1_at | 4.11 | Q5N9T4 | Hypothetical | Unknown Biological Process |
| GmaAffx.93616.1.S1_at | 4.15 | O24320 | Lipoxygenase | Other Metabolic Processes |
| Gma.12863.1.S1_at | 4.16 | Q5GQ66 | Alpha-dioxygenase | Response to Biotic Stimuli |
| GmaAffx.50095.4.S1_s_at | 4.34 | Q9C7F9 | Hypothetical | Unknown Biological Process |
| Gma.5496.1.S1_at | 4.44 | Q75GU4 | Hypothetical | Other Metabolic Processes |
| Gma.14850.1.S1_at | 4.70 | Q1S104 | Embryo-specific 3 | Unknown Biological Process |
| GmaAffx.50095.1.S1_at | 4.76 | Q39889 | Heat shock protein | Unknown Biological Process |
| Gma.10342.1.S1_at | 4.76 | Q84R94 | Hypothetical | Unknown Biological Process |
| Gma.17974.1.S1_at | 4.91 | Q43019 | Lipid transfer | Transport |
| GmaAffx.93591.1.S1_s_at | 4.93 | Q6A4W8 | Glutathione peroxidase | Electron Transport |
| GmaAffx.81791.1.S1_at | 5.00 |  | No UniProt ID | Developmental Processes |
| GmaAffx.92464.1.S1_s_at | 5.23 | Q5F304 | Beta-amylase | Other Metabolic Processes |
| GmaAffx.58494.1.S1_at | 5.30 | Q52QR3 | NAC domain |  |
| Gma.10073.1.A1_at | 5.31 | Q8LB81 | GDSL-motif lipase | Other Metabolic Processes |
| Gma.3189.2.S1_at | 5.31 | Q1SJ01 | Serine/threonine protein kinase | Protein Metabolism |
| GmaAffx.37908.1.A1_at | 5.70 | Q1S047 | Mini Chromosomal Maintinence | Protein Metabolism |
| GmaAffx.68386.1.S1_at | 5.85 | Q9LSD0 | Cathepsin B | Unknown Biological Process |
| Gma.3893.3.S1_at | 5.89 | Q8L799 | Inositol Oxygenase | Unknown Biological Process |
| GmaAffx.92185.1.S1_s_at | 6.23 | P05478 | Heat shock protein | Other Metabolic Processes |
| Gma.10897.1.S1_s_at | 6.30 | Q9LUV4 | Hydroxyproline-rich glycoprotein | Unknown Biological Process |
| Gma.12400.1.S1_at | 6.61 | Q1RVM0 | Hypothetical | Unknown Biological Process |
| GmaAffx.90206.1.S1_s_at | 6.66 | Q9ZSQ6 | T-complex protein | Electron Transport |
| Gma.15715.1.S1_at | 6.70 | Q1SL51 | CPRD12 protein | Transport |
| Gma.4305.1.S1_at | 6.80 | Q8S3F8 | S-adenosylmethionine decarboxylase | Other Metabolic Processes |
| GmaAffx.8505.1.A1_s_at | 6.80 |  | No UniProt ID | Protein Metabolism |
| Gma.14554.1.S1_at | 6.86 | Q1SJ63 | Hypothetical |  |
| Gma.3189.1.S1_a_at | 6.93 | O49858 | Cytochrome P450 | Protein Metabolism |
| Gma.1917.1.S1_at | 6.96 | Q1RX21 | Fibrillarin | Response to Abiotic Stimuli |
| GmaAffx.27787.1.A1_at | 7.17 |  | No UniProt ID |  |
| Gma.4305.3.S1_a_at | 7.48 | Q43854 | Peroxidase | Transport |
| Gma.11035.1.S1_at | 7.54 | Q9LMA8 | Hypothetical | Unknown Biological Process |
| GmaAffx.64505.1.A1_at | 8.11 |  | No UniProt ID |  |
| Gma.11191.1.S1_at | 8.47 | Q5ZBH8 | Auxin-induced protein | Other Biological Processes |
| Gma.13342.1.A1_at | 8.67 | P52581 | Isoflavone reductase | Unknown Biological Process |
| GmaAffx.51733.1.A1_at | 8.87 | Q8HQ04 | NADH dehydrogenase | Response to Stress |
| Gma.413.1.S1_s_at | 10.06 | Q9XFI8 | Peroxidase | Other Metabolic Processes |
| Gma.1917.1.S1_s_at | 11.10 | Q9FQE8 | Glutathione S-transferase | Response to Abiotic Stimuli |
| Gma.17989.2.S1_s_at | 11.90 | Q1SUM2 | Hypothetical | Unknown Biological Process |
| Gma.3888.2.S1_at | 12.91 | Q1SG42 | PsAD2 | Unknown Biological Process |
| GmaAffx.57970.2.S1_at | 13.12 | Q1SMF6 | Esterase | Developmental Processes |
| Gma.16812.1.S1_s_at | 13.48 | O23961 | Peroxidase | Other Metabolic Processes |
| GmaAffx.82745.1.S1_at | 13.80 | Q9FY93 | NAM-like |  |
| Gma.1594.1.S1_at | 17.66 | P93697 | CPRD12 protein | Response to Abiotic Stimuli |
| Gma.413.1.S1_at | 17.78 | Q1SBD4 | Chaperonin | Other Metabolic Processes |
| GmaAffx.6142.1.S1_at | 17.84 | Q8VZW5 | Dynein light chain | Response to Stress |
| Gma.10216.1.S1_at | 18.06 | Q2HTB5 | O-methyltransferase | Other Metabolic Processes |
| Gma.10216.3.A1_x_at | 18.18 | Q2HTB5 | O-methyltransferase | Other Metabolic Processes |
| GmaAffx.93342.1.S1_s_at | 18.27 | Q39827 | Arginine decarboxylase | Other Metabolic Processes |
| GmaAffx.89245.1.S1_s_at | 19.69 | P32110 | Glutathione S-transferase | Other Metabolic Processes |
| GmaAffx.88762.1.S1_at | 31.71 | P32110 | Glutathione S-transferase | Other Metabolic Processes |
| GmaAffx.88762.1.S1_x_at | 32.31 | P32110 | Glutathione S-transferase | Other Metabolic Processes |
| GmaAffx.87317.1.S1_s_at | 50.33 | Q1SWY3 | Hypothetical |  |
