# Supplementary material for: Integrating microarray analysis and the soybean genome to understand the soybeans iron deficiency response
Source: BMC Genomics. 2009 Aug 13;10:376. doi: 10.1186/1471-2164-10-376 (PMC2907705; doi:10.1186/1471-2164-10-376)
Supplement: Additional file 4 — Differentially Expressed Genes in Clusters identified in the Clark genotype with a sliding window of 1,000,000 bases. A table of differentially expressed genes in the Clark genotype illustrating the identified gene clusters using a sliding window of 1,000,000 bases, their chromosomal location, and gene annotation. [file 1471-2164-10-376-S4.doc]

Additional file 4: Differentially Expressed Genes in Clusters Identified in the Clark Genotype With a Sliding Window of 1,000,000 bases.

| Cluster Number | Affy Probe ID | Chromosome | UniProt Top Hit | Annotation |
| --- | --- | --- | --- | --- |
| 8_1 | Gma.4284.2.S1_s_at | 6 | Q1SMR6 | Cellular retinaldehyde binding/alpha-tocopherol transport |
| 8_1 | Gma.436.1.S1_at | 6 | Q07502 | 8.4 kDa sulfur-rich protein precursor (SE60 protein |
| 8_1 | Gma.6427.3.S1_a_at | 6 | Q39817 | Calnexin homolog precursor |
| 8_1 | GmaAffx.12694.1.S1_at | 6 | Q8H271 | Myb-like transcription factor 1 |
| 8_1 | GmaAffx.61002.1.S1_at | 6 | O65154 | RNA polymerase II transcriptional coactivator KIWI |
| 8_1 | GmaAffx.83638.1.S1_at | 6 | Q1SMQ2 | Hypothetical protein |
| 8_1 | GmaAffx.89896.1.S1_at | 6 | Q2HTU2 | Heat shock protein Hsp20 |
| 8_1 | GmaAffx.90865.1.S1_s_at | 6 | Q39817 | Calnexin homolog precursor |
| 7_2 | Gma.12974.1.S1_at | 12 | Q1SN75 | Beta-Ig-H3/fasciclin |
| 7_2 | Gma.16111.3.S1_at | 12 | O81221 | Actin |
| 7_2 | Gma.16984.1.A1_at | 12 | Q1RW87 | Hypothetical protein |
| 7_2 | Gma.9839.1.S1_at | 12 | Q41350 | Osmotin-like protein precursor |
| 7_2 | Gma.9839.2.S1_at | 12 | Q41350 | Osmotin-like protein precursor |
| 7_2 | GmaAffx.87358.1.S1_at | 12 | No Hit |  |
| 7_2 | GmaAffx.88571.1.A1_s_at | 12 | No Hit |  |
| 7_1 | Gma.12966.1.S1_at | 2 | Q67ZI9 | Putative GDSL-motif lipase/hydrolase |
| 7_1 | Gma.2776.2.S1_s_at | 2 | Q8W3Y3 | 1-aminocyclopropane-1-carboxylic acid oxidase |
| 7_1 | Gma.5632.1.S1_at | 2 | Q1S608 | E1 protein and Def2/Der2 allergen |
| 7_1 | Gma.6942.1.S1_at | 2 | Q9ZVN4 | Probable tyrosine-protein phosphatase At1g05000 (EC 3.1.3.48 |
| 7_1 | GmaAffx.13543.1.A1_at | 2 | O82074 | Beta-D-glucosidase precursor (EC 3.2.1.21 |
| 7_1 | GmaAffx.68770.2.S1_at | 2 | Q1SPW5 | Glycoside hydrolase, family 17 |
| 7_1 | GmaAffx.92536.1.S1_at | 2 | Q8W3Y3 | 1-aminocyclopropane-1-carboxylic acid oxidase |
| 6_2 | Gma.16913.1.S1_s_at | 9 | Q39887 | Proline-rich protein |
| 6_2 | Gma.79.1.S1_s_at | 9 | Q39887 | Proline-rich protein |
| 6_2 | Gma.79.2.S1_at | 9 | P08012 | Repetitive proline-rich cell wall protein 1 precursor |
| 6_2 | Gma.79.4.S1_s_at | 9 | Q39887 | Proline-rich protein |
| 6_2 | GmaAffx.77758.1.S1_x_at | 9 | Q39887 | Proline-rich protein |
| 6_2 | GmaAffx.84342.1.S1_x_at | 9 | O49226 | Proline-rich protein sbPRP1 (Fragment |
| 6_1 | Gma.14851.1.S1_at | 8 | Q4ABY1 | 4D11_6 |
| 6_1 | Gma.15113.1.S1_a_at | 8 | Q8RU51 | Glucan 1,3-beta-glucosidase (Cellulase, putative |
| 6_1 | Gma.1545.2.S1_at | 8 | O82515 | Mannitol dehydrogenase (EC 1.1.1.255 |
| 6_1 | Gma.6613.1.A1_at | 8 |  |  |
| 6_1 | GmaAffx.75159.1.S1_at | 8 | Q1SQB5 | Actin/actin-like |
| 6_1 | GmaAffx.84030.1.A1_at | 8 | Q1SJ61 | Zinc finger, RING-type; RINGv |
| 5_9 | Gma.12160.1.S1_at | 13 | Q5N800 | Hypothetical protein P0452F10.6 (Hypothetical protein P0443E07.35 |
| 5_9 | Gma.4589.2.S1_at | 13 | Q1RY98 | Hydroxymethylglutaryl-coenzyme A reductase, putative |
| 5_9 | GmaAffx.56975.1.S1_at | 13 | Q1RY98 | Hydroxymethylglutaryl-coenzyme A reductase, putative |
| 5_9 | GmaAffx.89783.1.S1_s_at | 13 | Q8W2E3 | 3-hydroxy-3-methylglutaryl coenzyme A |
| 5_9 | GmaAffx.91167.1.S1_s_at | 13 | Q9LS40 | CND41, chloroplast nucleoid DNA binding protein-like |
| 5_8 | Gma.1345.1.S1_at | 13 | No Hit |  |
| 5_8 | GmaAffx.23059.1.S1_at | 13 | Q9ZWS2 | Flavonoid 3-O-galactosyl transferase |
| 5_8 | GmaAffx.24879.1.S1_at | 13 | Q1SGA4 | Hypothetical protein |
| 5_8 | GmaAffx.28120.1.S1_at | 13 | Q9ZWS2 | Flavonoid 3-O-galactosyl transferase |
| 5_8 | GmaAffx.6250.1.A1_at | 13 | Q9M9F9 | Hypothetical protein At1g78430 |
| 5_7 | Gma.4438.3.S1_x_at | 6 | Q1T6K5 | 2OG-Fe(II |
| 5_7 | Gma.6550.2.S1_s_at | 6 | Q3SCM5 | Caffeic acid O-methyltransferase |
| 5_7 | Gma.6550.3.S1_s_at | 6 | P28002 | Caffeic acid 3-O-methyltransferase (EC 2.1.1.68 |
| 5_7 | Gma.7778.1.S1_at | 6 | O22880 | Hypothetical protein At2g40550 |
| 5_7 | GmaAffx.45805.2.S1_at | 6 | Q3SCM5 | Caffeic acid O-methyltransferase |
| 5_6 | Gma.4558.1.A1_at | 14 | Q1S8F9 | Lipolytic enzyme, G-D-S-L |
| 5_6 | Gma.6540.1.S1_at | 14 | Q1S8H7 | Alcohol dehydrogenase superfamily, zinc-containing |
| 5_6 | GmaAffx.76520.1.S1_at | 14 | P35016 | Endoplasmin homolog precursor |
| 5_6 | GmaAffx.87400.1.S1_at | 14 | Q94CH6 | Family II lipase EXL3 |
| 5_6 | GmaAffx.91200.1.S1_s_at | 14 | P35016 | Endoplasmin homolog precursor |
| 5_5 | Gma.17805.1.A1_s_at | 14 | Q1RXM7 | Haem peroxidase, plant/fungal/bacterial |
| 5_5 | GmaAffx.71811.1.S1_at | 14 | Q67UF5 | Hypothetical protein OJ1163_C07.26-2 |
| 5_5 | GmaAffx.82745.1.S1_at | 14 | No Hit |  |
| 5_5 | GmaAffx.87317.1.S1_at | 14 | No Hit |  |
| 5_5 | GmaAffx.89245.1.S1_s_at | 14 | No Hit |  |
| 5_4 | Gma.14319.1.A1_at | 14 | Q8W1X8 | Gamma-glutamyltransferase |
| 5_4 | Gma.4468.1.S1_at | 14 | Q2L8A7 | Acetoacetyl-CoA thiolase |
| 5_4 | Gma.8067.1.A1_at | 14 | Q1SRY0 | Hypothetical protein |
| 5_4 | GmaAffx.89899.1.S1_s_at | 14 | Q2L8A7 | Acetoacetyl-CoA thiolase |
| 5_4 | GmaAffx.90393.1.S1_s_at | 14 | Q2L8A7 | Acetoacetyl-CoA thiolase |
| 5_3 | Gma.6281.1.S1_at | 11 | Q7X9B3 | 9/13 hydroperoxide lyase |
| 5_3 | GmaAffx.37281.1.S1_at | 11 | Q1S047 | MCM; Nucleic acid-binding, OB-fold |
| 5_3 | GmaAffx.50239.1.A1_at | 11 | Q1S047 | MCM; Nucleic acid-binding, OB-fold |
| 5_3 | GmaAffx.70981.1.S1_at | 11 | Q3C1F4 | Nonsymbiotic hemoglobin |
| 5_3 | GmaAffx.91313.1.S1_s_at | 11 | Q7X9B3 | 9/13 hydroperoxide lyase |
| 5_2 | Gma.10581.1.S1_at | 8 | Q8GUC2 | Putative phosphatase |
| 5_2 | Gma.1532.1.S1_at | 8 | O49855 | Acid phosphatase |
| 5_2 | Gma.5786.2.S1_at | 8 | Q1SAY6 | Lipolytic enzyme, G-D-S-L |
| 5_2 | GmaAffx.89635.1.A1_s_at | 8 | O49855 | Acid phosphatase |
| 5_2 | GmaAffx.89772.9.A1_s_at | 8 | Q9LEN5 | Hypothetical protein |
| 5_13 | Gma.17606.2.S1_at | 10 | Q1SD30 | Hypothetical protein |
| 5_13 | Gma.17967.1.S1_at | 10 | No Hit |  |
| 5_13 | GmaAffx.83079.1.S1_at | 10 | No Hit |  |
| 5_13 | GmaAffx.90538.1.A1_s_at | 10 | Q9FSG7 | Thaumatin-like protein 1a precursor |
| 5_13 | GmaAffx.93126.1.S1_s_at | 10 | Q1S8N3 | Pyruvate kinase |
| 5_12 | Gma.12928.1.A1_at | 7 | No Hit |  |
| 5_12 | Gma.17372.3.S1_a_at | 7 | Q9XGM1 | Vacuolar ATP synthase subunit D |
| 5_12 | Gma.6638.2.S1_a_at | 7 | Q75GI1 | Putative symbiotic ammonium transport |
| 5_12 | GmaAffx.39734.2.S1_at | 7 | O81348 | Symbiotic ammonium transporter |
| 5_12 | GmaAffx.88134.1.S1_at | 7 | Q9FSH5 | B1-type cyclin dependent kinase |
| 5_11 | Gma.13352.1.S1_at | 18 | P19976 | Ferritin-1, chloroplast precursor |
| 5_11 | Gma.7642.1.A1_at | 18 | Q1SC78 | Ammonium transporter |
| 5_11 | GmaAffx.70608.1.S1_at | 18 | Q1S3G7 | UDP-glucuronosyl/UDP-glucosyltransferase |
| 5_11 | GmaAffx.75645.1.A1_at | 18 | Q1S2D1 | UDP-glucose glucosyltransferase |
| 5_11 | GmaAffx.93603.1.S1_s_at | 18 | P19976 | Ferritin-1, chloroplast precursor |
| 5_10 | Gma.12482.1.A1_at | 13 | Q8LC41 | Hypothetical protein |
| 5_10 | Gma.13259.2.S1_a_at | 13 | O22059 | Transcription factor CAPRICE |
| 5_10 | Gma.4220.2.S1_at | 13 | Q8LDW9 | Xyloglucan endotransglucosylase/hydrolase protein |
| 5_10 | Gma.795.1.A1_at | 13 | No Hit |  |
| 5_10 | GmaAffx.73405.1.S1_at | 13 | No Hit |  |
| 5_1 | Gma.48.1.S1_at | 5 | Q39889 | Heat shock protein |
| 5_1 | GmaAffx.23381.1.S1_at | 5 | Q9SD82 | Replication factor A-like protein |
| 5_1 | GmaAffx.39814.1.S1_at | 5 | Q9FME0 | Replication protein A1 |
| 5_1 | GmaAffx.59591.1.S1_s_at | 5 | O81016 | Probable pleiotropic drug resistance protein 4 |
| 5_1 | GmaAffx.92244.1.S1_at | 5 | O81016 | Probable pleiotropic drug resistance protein 4 |
| 4_9 | Gma.12483.1.A1_at | 2 | Q1SNI6 | Sugar transporter superfamily |
| 4_9 | Gma.15939.1.S1_at | 2 | Q8GW75 | Putative myb-related protein (MYB transcription factor |
| 4_9 | Gma.5461.1.S1_at | 2 | Q53B69 | Flavanone 3-hydroxylase (EC 1.14.11.9 |
| 4_9 | GmaAffx.70258.1.S1_s_at | 2 |  |  |
| 4_8 | Gma.5649.3.S1_a_at | 2 | Q1T4Z3 | Thioredoxin domain 2 |
| 4_8 | GmaAffx.18376.1.A1_at | 2 | Q677E9 | Ubi |
| 4_8 | GmaAffx.25607.1.A1_at | 2 | No Hit |  |
| 4_8 | GmaAffx.92441.1.S1_s_at | 2 | Q1SWS7 | TIR |
| 4_7 | Gma.11215.1.S1_at | 6 | Q41666 | Phloem specific protein |
| 4_7 | Gma.15233.1.S1_at | 6 | Q8VXF4 | Putative metallophosphatase |
| 4_7 | GmaAffx.16966.1.S1_at | 6 | Q8VXF4 | Putative metallophosphatase |
| 4_7 | GmaAffx.89821.1.A1_at | 6 | Q2I0H4 | Glyceraldehyde-3-phosphate dehydrogenase |
| 4_6 | Gma.1221.1.S1_s_at | 6 | P54233 | Inducible nitrate reductase [NADH] |
| 4_6 | Gma.13327.1.S1_at | 6 | Q2ENC2 | Chalcone synthase |
| 4_6 | Gma.13327.2.S1_a_at | 6 | P48399 | Chalcone synthase C |
| 4_6 | Gma.8416.1.S1_at | 6 | Q9SYR0 | Nitrate reductase |
| 4_5 | Gma.13796.1.A1_at | 4 | Q71BZ3 | Type-A response regulator |
| 4_5 | Gma.13796.2.S1_at | 4 | Q9FPR6 | Two-component response regulator ARR17 |
| 4_5 | Gma.9622.1.S1_at | 4 | P28002 | Caffeic acid 3-O-methyltransferase |
| 4_5 | GmaAffx.86552.2.S1_at | 4 | Q501D5 | At2g40550 |
| 4_4 | Gma.11871.2.S1_at | 11 | Q3Y6V1 | Cellulose synthase-like protein CslG |
| 4_4 | Gma.12096.1.A1_at | 11 | No Hit |  |
| 4_4 | Gma.17420.1.S1_at | 11 | No Hit |  |
| 4_4 | Gma.957.1.S1_at | 11 | Q27JA0 | Dirigent-like protein pDIR12 |
| 4_36 | Gma.13402.1.A1_at | 10 | Q75HS3 | Putative crooked neck protein |
| 4_36 | Gma.13994.1.A1_at | 10 | Q1S1C2 | Fatty acid elongase [imported]-Arabidopsis thaliana |
| 4_36 | Gma.15947.2.A1_at | 10 |  |  |
| 4_36 | Gma.7599.3.S1_at | 10 | Q944G0 | Mevalonate disphosphate decarboxylase |
| 4_35 | Gma.16827.1.S1_at | 10 | Q71UA1 | Iron-superoxide dismutase |
| 4_35 | Gma.2450.1.A1_at | 10 | Q43716 | Anthocyanidin 3-O-glucosyltransferase (EC 2.4.1.115 |
| 4_35 | GmaAffx.76187.1.S1_at | 10 | Q8LP23 | Rhamnosyl transferase |
| 4_35 | GmaAffx.85836.1.S1_at | 10 | Q9FPE8 | Hypothetical protein At1g25550 |
| 4_34 | Gma.10482.1.A1_a_at | 3 | Q6UEJ2 | Mini-chromosome maintenance 7 |
| 4_34 | Gma.10482.2.S1_a_at | 3 | Q6UEJ2 | Mini-chromosome maintenance 7 |
| 4_34 | Gma.406.5.S1_s_at | 3 | P49613 | S-adenosylmethionine synthetase 2 |
| 4_34 | GmaAffx.32647.1.S1_at | 3 | Q6UEJ2 | Mini-chromosome maintenance 7 |
| 4_33 | Gma.13058.1.A1_at | 7 | Q1RV95 | E-class P450, group I |
| 4_33 | Gma.17768.1.A1_at | 7 | Q1RV93 | Glycoside hydrolase, family 1 |
| 4_33 | Gma.338.2.S1_s_at | 7 | Q9XFI6 | Peroxidase |
| 4_33 | GmaAffx.34532.1.S1_at | 7 | Q1RV93 | Glycoside hydrolase, family 1 |
| 4_32 | Gma.12365.2.S1_at | 7 | No Hit |  |
| 4_32 | GmaAffx.12887.1.S1_at | 7 | Q84YI1 | Polyphenol oxidase |
| 4_32 | GmaAffx.20332.1.A1_at | 7 | No Hit |  |
| 4_32 | GmaAffx.92420.1.S1_s_at | 7 | P04793 | 17.5 kDa class I heat shock protein |
| 4_31 | Gma.1127.1.S1_at | 7 | No Hit |  |
| 4_31 | Gma.16552.1.A1_s_at | 7 | No Hit |  |
| 4_31 | GmaAffx.26732.1.A1_at | 7 | No Hit |  |
| 4_31 | GmaAffx.84474.1.S1_at | 7 | Q8LBR3 | Alcohol dehydrogenase, putative |
| 4_30 | Gma.12599.1.S1_at | 7 | P42814 | Ribonuclease 2 precursor |
| 4_30 | Gma.7623.1.A1_at | 7 | Q6K237 | Hypothetical protein B1178F07.20 |
| 4_30 | GmaAffx.24470.1.S1_at | 7 | Q8LJC6 | Putative UDP-glucosyltransferase |
| 4_30 | GmaAffx.91725.1.S1_at | 7 | Q1SI90 | Disease resistance protein; AAA ATPase |
| 4_3 | Gma.2141.1.S1_at | 11 | Q1S2D1 | UDP-glucose glucosyltransferase |
| 4_3 | Gma.5205.2.S1_at | 11 | Q1S7M1 | AT5g55180/MCO15_13, putative |
| 4_3 | GmaAffx.25369.1.S1_s_at | 11 | No Hit |  |
| 4_3 | GmaAffx.82240.1.S1_at | 11 | Q1S2F4 | UDP-glucose glucosyltransferase |
| 4_29 | Gma.10216.1.S1_at | 7 | Q2HTB5 | O-methyltransferase, family 2 |
| 4_29 | Gma.10216.2.S1_at | 7 | Q2HTB5 | O-methyltransferase, family 2 |
| 4_29 | Gma.10216.3.A1_x_at | 7 | Q2HTB5 | O-methyltransferase, family 2 |
| 4_29 | Gma.4162.1.S1_at | 7 | Q9C9E1 | Hypothetical protein T10D10.10 |
| 4_28 | Gma.11166.1.S1_at | 7 | O24320 | Lipoxygenase |
| 4_28 | Gma.16500.1.S1_at | 7 | P27480 | Lipoxygenase 1 |
| 4_28 | GmaAffx.81415.1.S1_at | 7 | O24320 | Lipoxygenase |
| 4_28 | GmaAffx.93591.1.S1_s_at | 7 | O24320 | Lipoxygenase |
| 4_27 | Gma.1359.1.S1_at | 7 | Q8GUC2 | Putative phosphatase |
| 4_27 | GmaAffx.16212.1.S1_at | 7 | P10742 | Stem 31 kDa glycoprotein precursor (Vegetative storage protein VSP25 |
| 4_27 | GmaAffx.90263.1.S1_s_at | 7 | P24095 | Seed lipoxygenase |
| 4_27 | GmaAffx.90937.1.A1_at | 7 | P24095 | Seed lipoxygenase |
| 4_26 | Gma.12584.2.A1_at | 19 | Q1SUU0 | AMP-dependent synthetase and ligase |
| 4_26 | Gma.406.4.S1_at | 19 | Q1SFW5 | S-adenosylmethionine synthetase |
| 4_26 | Gma.4707.3.S1_s_at | 19 | Q677H6 | ADP-ribosylation factor |
| 4_26 | GmaAffx.28196.2.A1_s_at | 19 | No Hit |  |
| 4_25 | Gma.3189.1.S1_at | 9 | Q1SJ01 | Serine/threonine protein kinase, active site |
| 4_25 | Gma.385.1.S1_at | 9 | Q9ZR53 | Annexin-like protein |
| 4_25 | GmaAffx.56061.1.S1_at | 9 | Q1SNV1 | SAM |
| 4_25 | GmaAffx.89971.1.S1_s_at | 9 | Q9ZR53 | Annexin-like protein |
| 4_24 | Gma.13918.1.A1_at | 9 | Q1SSW0 | Protein phosphatase 2C |
| 4_24 | Gma.17032.1.S1_at | 9 | Q9AYM8 | CPRD2 protein |
| 4_24 | GmaAffx.55661.1.S1_at | 9 | Q9FGX1 | ATP-citrate lyase subunit B |
| 4_24 | GmaAffx.8902.1.A1_at | 9 | Q1S4Z1 | Protein tyrosine kinase |
| 4_23 | Gma.11026.1.S1_at | 16 | Q1SMD8 | Kunitz inhibitor ST1-like |
| 4_23 | Gma.6231.1.S1_at | 16 | Q8RVX2 | Protease inhibitor precursor |
| 4_23 | GmaAffx.87089.1.S1_at | 16 | Q60D21 | Putative kinase interacting protein |
| 4_23 | GmaAffx.93550.1.S1_at | 16 | Q6TKR0 | Ribosomal protein L3A |
| 4_22 | Gma.15856.1.S1_at | 16 | Q9FVC2 | Apyrase GS52 |
| 4_22 | Gma.15856.2.S1_at | 16 | Q9FVC2 | Apyrase GS52 |
| 4_22 | Gma.4386.1.S1_at | 16 | Q9ZWQ5 | UDP-glycose:flavonoid glycosyltransferase |
| 4_22 | GmaAffx.93030.1.S1_s_at | 16 | Q3LHL1 | Short chain dehydrogenase |
| 4_21 | Gma.17733.1.S1_s_at | 20 | Q1S278 | Proteinase inhibitor I13 |
| 4_21 | Gma.4218.2.S1_a_at | 20 | Q1S275 | Targeting for Xklp2 |
| 4_21 | Gma.7646.2.S1_at | 20 | Q9FHM8 | Receptor-like protein kinase |
| 4_21 | Gma.7880.1.S1_at | 20 | Q1S265 | Hypothetical protein |
| 4_20 | GmaAffx.52252.1.A1_at | 12 | No Hit |  |
| 4_20 | GmaAffx.59986.1.A1_at | 12 | No Hit |  |
| 4_20 | GmaAffx.59986.2.S1_at | 12 | Q94C37 | At1g05230/YUP8H12_16 |
| 4_20 | GmaAffx.93451.1.S1_s_at | 12 | Q2PEW2 | Putative cytoplasmic aconitate hydratase |
| 4_2 | Gma.10689.1.S1_at | 8 | P25272 | Kunitz-type trypsin inhibitor |
| 4_2 | Gma.5510.2.S1_s_at | 8 | Q9XHC6 | Cytochrome P450 monooxygenaseCYP93D1 |
| 4_2 | Gma.9202.1.S1_at | 8 | P25272 | Kunitz-type trypsin inhibitor KTI1 precursor |
| 4_2 | GmaAffx.64606.1.S1_at | 8 | Q9MBB5 | Beta-1,3-glucanase |
| 4_19 | Gma.13140.1.A1_at | 12 | Q1SCN9 | Aldehyde dehydrogenase |
| 4_19 | Gma.13140.4.S1_at | 12 | Q1SCN9 | Aldehyde dehydrogenase |
| 4_19 | Gma.2096.2.S1_a_at | 12 | Q9ZW03 | Tropinone reductase |
| 4_19 | Gma.2096.3.S1_s_at | 12 | Q9ASX2 | Tropinone reductase homolog At1g07440 |
| 4_18 | Gma.11848.1.S1_at | 13 | Q1RX96 | Monogalactosyldiacylglycerol synthase |
| 4_18 | Gma.12014.1.A1_at | 13 | Q58IU5 | Non-intrinsic ABC protein |
| 4_18 | Gma.529.1.S1_at | 13 | Q6YGT9 | Purple acid phosphatase-like protein |
| 4_18 | Gma.529.2.S1_at | 13 | Q764C1 | Acid phosphatase precursor |
| 4_17 | GmaAffx.12887.2.S1_at | 13 | Q84YI1 | Polyphenol oxidase |
| 4_17 | GmaAffx.66617.1.S1_at | 13 | O82432 | Leucine-rich receptor-like protein kinase |
| 4_17 | GmaAffx.69311.1.S1_at | 13 | P04794 | 17.5 kDa class I heat shock protein |
| 4_17 | GmaAffx.93268.1.S1_at | 13 | P04794 | 17.5 kDa class I heat shock protein |
| 4_16 | Gma.3893.3.S1_at | 13 | Q9LMA8 | T29M8.5 protein |
| 4_16 | GmaAffx.6142.1.S1_at | 13 | Q4ZJ73 | 12-oxophytodienoate reductase |
| 4_16 | GmaAffx.83805.1.S1_at | 13 | Q949S3 | Hypothetical protein At3g42725 |
| 4_16 | GmaAffx.93442.1.S1_at | 13 | Q4ZJ73 | 12-oxophytodienoate reductase |
| 4_15 | Gma.4457.1.S1_at | 15 | Q9LZJ5 | Multidrug resistance-associated protein 10 |
| 4_15 | Gma.6081.1.S1_at | 15 | Q6JX03 | Chitinase-like protein |
| 4_15 | GmaAffx.43575.1.S1_at | 15 | Q9MB25 | S1-1 protein |
| 4_15 | GmaAffx.93250.1.S1_at | 15 | Q93YH4 | ATP citrate lyase a-subunit |
| 4_14 | Gma.16812.1.S1_s_at | 15 | O23961 | Peroxidase precursor |
| 4_14 | GmaAffx.42517.1.A1_at | 15 | Q1RVA3 | Hypothetical protein |
| 4_14 | GmaAffx.85292.1.S1_at | 15 | Q6Z671 | Chloroplast nucleoid DNA-binding protein |
| 4_14 | GmaAffx.91168.1.S1_at | 15 | O23961 | Peroxidase precursor |
| 4_13 | Gma.10752.2.S1_at | 15 | Q2HUA9 | Lipase/lipooxygenase, PLAT/LH2 |
| 4_13 | GmaAffx.3021.1.A1_at | 15 | No Hit |  |
| 4_13 | GmaAffx.43239.1.S1_at | 15 | Q75GI1 | Putative symbiotic ammonium transport protein |
| 4_13 | GmaAffx.93522.1.S1_s_at | 15 | Q9M631 | Cinnamoyl CoA reductase |
| 4_12 | Gma.17369.1.S1_at | 2 |  |  |
| 4_12 | Gma.3216.2.S1_at | 2 | O48781 | Hypothetical protein At2g26660 |
| 4_12 | Gma.3216.3.A1_at | 2 | Q8GWZ3 | Hypothetical protein At2g26660 |
| 4_12 | GmaAffx.51889.1.S1_at | 2 | Q9SUV2 | Hypothetical protein F8B4.90 |
| 4_11 | Gma.13296.3.S1_at | 2 | Q8H1Z0 | Cuticle protein |
| 4_11 | GmaAffx.15794.1.S1_at | 2 | Q5MJZ4 | 1-deoxy-D-xylulose-5-phosphate synthase |
| 4_11 | GmaAffx.30428.1.S1_at | 2 | P26413 | Heat shock 70 kDa protein |
| 4_11 | GmaAffx.63464.1.S1_at | 2 | No Hit |  |
| 4_10 | Gma.12692.1.A1_at | 2 | No Hit |  |
| 4_10 | Gma.3043.1.S1_at | 2 | Q9S728 | En/Spm-like transposon protein |
| 4_10 | GmaAffx.46567.1.S1_at | 2 | Q9FJ26 | DNA polymerase alpha subunit IV |
| 4_10 | GmaAffx.88242.1.S1_at | 2 | Q9SUN5 | snRNP protein |
| 4_1 | Gma.11336.1.S1_at | 5 | Q1SSC0 | Thaumatin, pathogenesis-related |
| 4_1 | Gma.13045.1.S1_at | 5 | Q707M7 | Acid phosphatase |
| 4_1 | Gma.6962.2.S1_at | 5 | Q8VYI4 | Aspartate-semialdehyde dehydrogenase, putative |
| 4_1 | GmaAffx.93041.1.S1_s_at | 5 | Q1SSC0 | Thaumatin, pathogenesis-related |
