# Supplementary material for: Integrating microarray analysis and the soybean genome to understand the soybeans iron deficiency response
Source: BMC Genomics. 2009 Aug 13;10:376. doi: 10.1186/1471-2164-10-376 (PMC2907705; doi:10.1186/1471-2164-10-376)
Supplement: Additional file 5 — Differentially Expressed Genes in Clusters identified in the IsoClark genotype with a sliding window of 1,000,000 bases. A table of differentially expressed genes in the IsoClark genotype illustrating the identified gene clusters using a sliding window of 1,000,000 bases, their chromosomal location, and gene annotation. [file 1471-2164-10-376-S5.doc]

Additional file 5: Differentially Expressed Genes in Clusters Identified in the IsoClark Genotype With a Sliding Window of 1,000,000 Bases.

| Cluster Number | Affy Probe ID | Chromosome | UniProt Top Hit | Annotation |
| --- | --- | --- | --- | --- |
| 4_1 | Gma.13058.2.S1_at | 17 | Q1RV95 | E-class P450 |
| 4_1 | Gma.12665.1.S1_at | 17 | O23414 | Hypothetical protein |
| 4_1 | Gma.12665.2.A1_at | 17 |  |  |
| 4_1 | Gma.15538.1.S1_at | 17 | Q8L8Z8 | Glutaredoxin |
| 4_2 | GmaAffx.53903.1.A1_at | 17 |  |  |
| 4_2 | Gma.13296.3.S1_at | 17 | Q8H1Z0 | YORE-YORE protein |
| 4_2 | GmaAffx.63464.1.S1_at | 17 |  |  |
| 4_2 | GmaAffx.30428.1.S1_at | 17 | P26413 | Heat shock 70 kDa protein |
| 4_3 | Gma.4534.1.S1_at | 15 | Q1SG45 | Hypothetical |
| 4_3 | Gma.3766.1.S1_at | 15 | Q1SG42 | PsAD2 |
| 4_3 | Gma.10104.1.S1_at | 15 |  |  |
| 4_3 | Gma.4457.1.S1_a_at | 15 | Q9LZJ5 | Multidrug resistance-associated protein |
| 4_4 | GmaAffx.30771.1.S1_at | 13 | Q9FNI1 | B-type cyclin |
| 4_4 | Gma.768.1.S1_at | 13 | Q6NLE8 | Hypothetical protein |
| 4_4 | GmaAffx.87934.1.S1_at | 13 | Q1T1D0 | Terpene synthase |
| 4_4 | Gma.625.1.S1_at | 13 | Q1T1D0 | Terpene synthase |
| 4_5 | Gma.3539.2.S1_at | 9 | Q9STY1 | Hypothetical |
| 4_5 | GmaAffx.4270.1.S1_s_at | 9 |  |  |
| 4_5 | Gma.2554.1.S1_at | 9 | Q652J5 | Deoxyribodipyrimidine photolyase |
| 4_5 | Gma.7557.1.S1_at | 9 | Q8H7E2 | Hypothetical |
| 4_6 | GmaAffx.33640.1.S1_at | 19 | Q1SFG3 | Transferase |
| 4_6 | GmaAffx.82795.1.S1_at | 19 | Q8LFH3 | Hypothetical |
| 4_6 | Gma.17724.3.S1_at | 19 | Q5J7N0 | GSDL-motif lipase |
| 4_6 | Gma.17724.1.A1_at | 19 |  |  |
| 4_7 | Gma.12270.1.S1_at | 3 | Q5I2Q5 | alcohol acyl-transferase |
| 4_7 | GmaAffx.49404.1.S1_at | 3 | Q8GT20 | Benzoyl coenzyme A |
| 4_7 | Gma.17724.2.S1_at | 3 | Q9M8Y5 | GDSL-motif lipase |
| 4_7 | Gma.12675.1.A1_at | 3 | Q8LB81 | GDSL-motif lipase |
