# Supplementary material for: Integrating microarray analysis and the soybean genome to understand the soybeans iron deficiency response
Source: BMC Genomics. 2009 Aug 13;10:376. doi: 10.1186/1471-2164-10-376 (PMC2907705; doi:10.1186/1471-2164-10-376)
Supplement: Additional file 6 — Identified and annotated SFPs between two NILs Soybean Genome Chip consensus sequences. A table identifying the Affymetrix probes containing a SFP between the Clark and IsoClark genotypes. The data also identifies the chromosome containing the identified SFP and the annotation of the gene containing the SFP. [file 1471-2164-10-376-S6.doc]

Additional file 6: Identified SFPs

| Affy Probe ID containing SFP | Chromosome | Best Hit UniProt ID | GO TERM | GO Annotation |
| --- | --- | --- | --- | --- |
| Gma.978.1.S1_at | 1 | Q6H4P7 | GO:0006418 | ATP binding |
| GmaAffx.83700.1.S1_at | 1 | Q1S6W0 | GO:0016787 | Hydrolase Activity |
| Gma.2101.1.S1_at | 1 | Q9M6N2 | GO:0016301 | Kinase Activity |
| GmaAffx.78358.2.S1_at | 1 | Q948H2 | GO:0019252 | Starch Biosynthesis |
| GmaAffx.28966.1.S1_at | 1 | No UniProt Hit |  | No UniProt Hit |
| GmaAffx.61076.1.S1_at | 1 | No UniProt Hit |  | No UniProt Hit |
| Gma.4188.3.S1_a_at | 1 | Q8RWD4 | GO:0005554 | Unknown Function |
| GmaAffx.52400.1.S1_s_at | 1 | Q9FHY6 | GO:0005554 | Unknown Function |
| GmaAffx.92301.1.S1_s_at | 1 | Q1SZU3 | GO:0005554 | Unknown Function |
| Gma.2424.2.S1_a_at | 2 | Q1SSV5 | GO:0006914 | Autophagy |
| Gma.10827.3.S1_at | 2 | Q9LJ66 | GO:0005506 | Cation transport |
| Gma.17592.1.S1_at | 2 | P49299 | GO:0004108 | Citrate Synthase |
| GmaAffx.91660.1.S1_at | 2 | Q6RIB6 | GO:0016615 | Dehydrogenase |
| GmaAffx.86105.1.S1_at | 2 | Q71QD5 |  | Protein Metabolism |
| GmaAffx.1301.47.A1_at | 2 | No UniProt Hit | GO:0003700 | Transcription Factor |
| Gma.13209.1.S1_at | 2 | No UniProt Hit |  | No UniProt Hit |
| Gma.14204.1.A1_at | 2 | No UniProt Hit |  | No UniProt Hit |
| Gma.2811.1.S1_at | 2 | No UniProt Hit |  | No UniProt Hit |
| Gma.3997.1.S1_at | 2 | Q1S258 |  | No UniProt Hit |
| GmaAffx.53416.1.S1_at | 2 | No UniProt Hit |  | No UniProt Hit |
| GmaAffx.82973.1.S1_at | 2 | No UniProt Hit |  | No UniProt Hit |
| GmaAffx.83133.1.S1_at | 2 | No UniProt Hit |  | No UniProt Hit |
| Gma.3265.1.S1_at | 2 | Q6EP64 | GO:0005554 | Unknown Function |
| GmaAffx.72674.1.S1_at | 2 | Q9SU16 | GO:0005554 | Unknown Function |
| Gma.1955.4.A1_at | 3 | Q5PYQ5 | GO:0005509 | Cation transport |
| Gma.7757.1.S1_at | 3 | Q93XA0 | GO:0003700 | Transcription Factor |
| Gma.7757.1.S1_at | 3 | Q93XA0 | GO:0003700 | Transcription Factor |
| GmaAffx.47461.1.S1_at | 3 | Q1RWC1 | GO:0005554 | Unknown Function |
| GmaAffx.3025.1.S1_at | 4 | Q69ST5 | GO:0005524 | ATP binding |
| Gma.2977.2.S1_a_at | 4 | Q9FKI4 | GO:0008080 | N-acetyltransferase activity |
| Gma.17343.1.A1_at | 4 | No UniProt Hit |  | No UniProt Hit |
| GmaAffx.8019.1.S1_at | 4 | No UniProt Hit |  | No UniProt Hit |
| Gma.404.1.A1_at | 4 | Q76DY0 | GO:0003700 | Transcription Factor |
| Gma.7761.1.S1_a_at | 4 | Q9SLZ4 | GO:0003700 | Transcription Factor |
| Gma.3712.1.S1_s_at | 4 | Q7XYY0 |  | Unknown Function |
| Gma.15061.1.S1_at | 5 | P35100 | GO:0005524 | ATP binding |
| GmaAffx.50907.1.S1_at | 5 | Q1SMQ9 | GO:0005509 | Cation transport |
| GmaAffx.61337.1.S1_at | 5 | Q2HU62 | GO:0004553 | Hydrolase Activity |
| Gma.8011.2.S1_a_at | 5 | Q9FV81 | GO:0006412 | Protein Biosynthesis |
| GmaAffx.93614.1.S1_at | 5 | Q1RV18 | GO:0005515 | Protein Folding |
| GmaAffx.82088.1.S1_at | 5 | Q6ZGX8 | GO:0006886 | Protein Transport |
| GmaAffx.90942.1.S1_at | 5 | Q40203 | GO:0015031 | Protein Transport |
| Gma.4040.1.S1_at | 5 | Q1RY71 | GO:0003700 | Transcription Factor |
| Gma.2069.1.S1_at | 5 | Q6Z918 | GO:0004306 | Transferase |
| Gma.12885.1.A1_at | 5 | No UniProt Hit |  | No UniProt Hit |
| Gma.5163.2.S1_at | 5 | Q1SBT2 | GO:0005554 | Unknown Function |
| GmaAffx.11464.1.S1_at | 5 | Q9LS48 | GO:0005554 | Unknown Function |
| GmaAffx.85311.1.S1_at | 5 | Q9FMN3 | GO:0005554 | Unknown Function |
| GmaAffx.3025.1.S1_at | 6 | Q69ST5 | GO:0006418 | ATP binding |
| GmaAffx.20345.1.S1_at | 6 | Q1T0B2 | GO:0016126 | Sterol Biosynthesis |
| Gma.4461.1.S1_at | 6 | No UniProt Hit |  | No UniProt Hit |
| GmaAffx.19097.1.S1_at | 6 | No UniProt Hit |  | No UniProt Hit |
| Gma.11156.1.S1_s_at | 6 | Q5JML5 | GO:0005554 | Unknown Function |
| Gma.3712.1.S1_s_at | 6 | Q7XYY0 | GO:0005554 | Unknown Function |
| Gma.5866.1.S1_x_at | 6 | Q9XIA7 | GO:0005554 | Unknown Function |
| Gma.8653.1.A1_at | 6 | Q9LXZ7 | GO:0005554 | Unknown Function |
| GmaAffx.31836.1.S1_at | 6 | Q8LCV7 | GO:0005554 | Unknown Function |
| GmaAffx.84246.1.S1_at | 6 | Q9LYD7 | GO:0005554 | Unknown Function |
| Gma.4374.1.S1_s_at | 7 | Q2MJ15 | GO:0005506 | Cation transport |
| Gma.5994.1.S1_at | 7 | Q1SRH3 | GO:0004869 | Cysteine Protease |
| GmaAffx.45517.1.S1_at | 7 | Q9M3Y4 | GO:0045735 | Nutrient Reservoir Activity |
| Gma.8776.1.S1_at | 7 | Q1SG75 | GO:0016491 | Oxidoreductase |
| Gma.12223.1.S1_at | 7 | Q1SF86 | GO:0005515 | Protein Folding |
| Gma.8911.1.S1_at | 7 | Q9SAG7 | GO:0003723 | RNA Binding |
| Gma.7058.1.A1_at | 7 | Q1T4P7 | GO:0003700 | Transcription Factor |
| GmaAffx.77266.1.S1_at | 7 | No UniProt Hit |  | No UniProt Hit |
| Gma.1289.1.S1_at | 7 | Q05929 | GO:0005554 | Unknown Function |
| Gma.7768.1.A1_s_at | 7 | Q9SH93 | GO:0005554 | Unknown Function |
| GmaAffx.26598.1.S1_at | 7 | Q9LFC2 | GO:0005554 | Unknown Function |
| Gma.4456.1.S1_at | 8 | Q9MUK5 | GO:0004040 | Amidase Activity |
| Gma.16024.1.S1_s_at | 8 | Q1T4L1 | GO:0015144 | Carbohydrate Transport |
| GmaAffx.79966.1.S1_at | 8 | Q2QQ50 | GO:0003824 | catalytic_activity |
| GmaAffx.50907.1.S1_at | 8 | Q1SMQ9 | GO:0005509 | Cation transport |
| GmaAffx.93166.1.S1_s_at | 8 | Q69F96 | GO:0016760 | Cellulose Synthase |
| Gma.181.1.S1_at | 8 | Q93Y50 | GO:0006633 | fatty_acid_biosynthesis |
| GmaAffx.31381.1.S1_at | 8 | Q8VYN6 | GO:0006096 | Glycolysis |
| GmaAffx.61337.1.S1_at | 8 | Q2HU62 | GO:0004553 | Hydrolase Activity |
| GmaAffx.79733.1.S1_s_at | 8 | Q39898 |  | Kunitz trypsin inhibitor |
| Gma.8776.1.S1_at | 8 | Q1SG75 | GO:0016491 | Oxidoreductase |
| Gma.8011.2.S1_a_at | 8 | Q9FV81 | GO:0006412 | Protein Biosynthesis |
| Gma.8911.1.S1_at | 8 | Q9SAG7 | GO:0003723 | RNA Binding |
| GmaAffx.74113.1.S1_at | 8 | Q58I04 | GO:0015770 | Sugar Transport |
| Gma.3510.1.S1_at | 8 | Q39658 | GO:0003700 | Transcription Factor |
| Gma.2069.1.S1_at | 8 | Q6Z918 | GO:0004306 | Transferase |
| GmaAffx.41460.1.S1_at | 8 | No UniProt Hit |  | No UniProt Hit |
| GmaAffx.64946.1.S1_at | 8 | No UniProt Hit |  | No UniProt Hit |
| GmaAffx.85112.1.S1_at | 8 | Q7XPH4 | GO:0005554 | Unknown Function |
| Gma.16023.2.S1_at | 9 | Q9FMX7 | GO:0003824 | catalytic_activity |
| Gma.1909.1.S1_at | 9 | No UniProt Hit |  | No UniProt Hit |
| GmaAffx.27319.1.S1_at | 9 | P93045 | GO:0005515 | Protein Folding |
| GmaAffx.77832.1.S1_at | 9 | Q9FX34 | GO:0006499 | Protein Metabolism |
| GmaAffx.18381.1.S1_at | 9 | Q9FI73 | GO:0006810 | Transport |
| Gma.3700.2.S1_at | 9 | O22164 | GO:0005554 | Unknown Function |
| Gma.4653.1.S1_at | 9 | Q5YJQ1 | GO:0005554 | Unknown Function |
| GmaAffx.72771.1.A1_at | 10 | Q1SKU8 | GO:0008270 | Cation transport |
| Gma.15264.1.S1_at | 10 | Q1SM16 | GO:0003676 | Nucleic Acid Binding |
| GmaAffx.76755.1.S1_at | 10 | Q8L960 | GO:0003723 | RNA Binding |
| Gma.5974.1.S1_at | 10 | Q1SCD5 | GO:0005525 | Translation |
| GmaAffx.92783.1.S1_s_at | 10 | Q58I24 | GO:0003746 | Translation |
| Gma.16925.1.A1_at | 10 | No UniProt Hit |  | No UniProt Hit |
| Gma.2811.1.S1_at | 10 | No UniProt Hit |  | No UniProt Hit |
| Gma.3997.1.S1_at | 10 | Q1S258 |  | No UniProt Hit |
| GmaAffx.20697.1.S1_at | 10 | No UniProt Hit |  | No UniProt Hit |
| GmaAffx.4898.2.S1_at | 10 | No UniProt Hit |  | No UniProt Hit |
| GmaAffx.51828.1.S1_s_at | 10 | No UniProt Hit |  | No UniProt Hit |
| GmaAffx.60948.1.S1_at | 10 | No UniProt Hit |  | No UniProt Hit |
| GmaAffx.67495.1.S1_at | 10 | No UniProt Hit |  | No UniProt Hit |
| Gma.978.1.S1_at | 11 | Q6H4P7 | GO:0005524 | ATP binding |
| Gma.2101.1.S1_at | 11 | Q9M6N2 | GO:0016301 | Kinase Activity |
| Gma.2977.2.S1_a_at | 11 | Q9FKI4 | GO:0008080 | N-acetyltransferase activity |
| Gma.6914.1.A1_at | 11 | Q1RW61 | GO:0006388 | Translation |
| Gma.17504.1.S1_at | 11 | No UniProt Hit |  | No UniProt Hit |
| Gma.9816.1.S1_at | 11 | No UniProt Hit |  | No UniProt Hit |
| GmaAffx.80241.1.S1_s_at | 11 | No UniProt Hit |  | No UniProt Hit |
| Gma.5866.1.S1_x_at | 11 | Q9XIA7 | GO:0005554 | Unknown Function |
| GmaAffx.92301.1.S1_s_at | 11 | Q1SZU3 | GO:0005554 | Unknown Function |
| GmaAffx.47078.1.A1_at | 11 | Q6Z1G7 | GO:0004739 | Dehydrogenase |
| Gma.2977.2.S1_a_at | 11 | Q9FKI4 | GO:0008080 | N-acetyltransferase activity |
| GmaAffx.80323.2.S1_at | 11 | No UniProt Hit |  | No UniProt Hit |
| Gma.1477.1.S1_at | 11 | Q1SZZ7 | GO:0005554 | Unknown Function |
| Gma.3265.1.S1_at | 11 | Q6EP64 | GO:0005554 | Unknown Function |
| GmaAffx.38910.1.S1_at | 11 | Q1SS74 | GO:0005554 | Unknown Function |
| GmaAffx.47461.1.S1_at | 11 | Q1RWC1 | GO:0005554 | Unknown Function |
| GmaAffx.72674.1.S1_at | 11 | Q9SU16 | GO:0005554 | Unknown Function |
| GmaAffx.88213.1.S1_at | 11 | Q8VY47 | GO:0005554 | Unknown Function |
| Gma.5734.1.S1_at | 12 | Q7XY14 | GO:0017004 | Cytochrome |
| Gma.6749.1.S1_at | 12 | Q2HSH7 | GO:0004407 | Histone Deaceetylase |
| Gma.14194.1.A1_at | 12 | Q8GYC2 | GO:0016925 | Protein Metabolism |
| Gma.16753.1.S1_at | 12 | Q9SAA2 | GO:0006508 | proteolysis |
| Gma.13456.1.S1_at | 12 | Q93YQ0 | GO:0003723 | RNA Binding |
| Gma.10222.1.A1_at | 12 | No UniProt Hit |  | No UniProt Hit |
| Gma.5866.1.S1_x_at | 12 | Q9XIA7 | GO:0005554 | Unknown Function |
| Gma.3904.1.S1_x_at | 13 | P49597 | GO:0003824 | catalytic_activity |
| GmaAffx.15192.1.S1_at | 13 | Q1T1I6 | GO:0006812 | Cation Transport |
| GmaAffx.31689.1.S1_at | 13 | Q9SYM7 | GO:0005506 | Cation Transport |
| Gma.13363.1.S1_s_at | 13 | Q1PCR8 | GO:0006118 | Electron Transport |
| GmaAffx.5496.1.S1_at | 13 | Q6S4R9 | GO:0016787 | Hydrolase Activity |
| Gma.9902.1.A1_at | 13 | Q94JU3 | GO:0004708 | Kinase Activity |
| GmaAffx.40133.1.S1_at | 13 | Q1SMG8 | GO:0016829 | Lyase Activity |
| GmaAffx.7213.1.A1_at | 13 | Q8S3S6 | GO:0003676 | Nucleic Acid Binding |
| Gma.14194.1.A1_at | 13 | Q8GYC2 | GO:0016925 | Protein Metabolism |
| Gma.16526.1.S1_at | 13 | Q2R4U5 | GO:0006508 | proteolysis |
| Gma.8911.1.S1_at | 13 | Q9SAG7 | GO:0003723 | RNA Binding |
| GmaAffx.92612.1.S1_s_at | 13 | Q2PEV8 | GO:0003723 | RNA Binding |
| Gma.4463.1.S1_at | 13 | Q6TKQ3 | GO:0006355 | Transcription Factor |
| Gma.7761.1.S1_a_at | 13 | Q9SLZ4 | GO:0003700 | Transcription Factor |
| GmaAffx.51287.1.S1_at | 13 | P56820 | GO:0003743 | Translation |
| Gma.9401.1.S1_at | 13 | No UniProt Hit |  | No UniProt Hit |
| GmaAffx.1268.1.S1_at | 13 | No UniProt Hit |  | No UniProt Hit |
| Gma.4188.3.S1_a_at | 13 | Q8RWD4 | GO:0005554 | Unknown Function |
| GmaAffx.16959.1.S1_s_at | 13 | Q9FN38 | GO:0005554 | Unknown Function |
| GmaAffx.73763.1.S1_at | 13 | Q9LKA5 | GO:0005554 | Unknown Function |
| GmaAffx.75685.1.S1_at | 13 | Q2HWC9 | GO:0005554 | Unknown Function |
| GmaAffx.88213.1.S1_at | 13 | Q8VY47 | GO:0005554 | Unknown Function |
| Gma.3204.1.S1_at | 15 | Q6PP98 | GO:0006418 | ATP binding |
| Gma.8774.1.A1_at | 15 | Q6ATY7 | GO:0015087 | Cation Transport |
| Gma.13363.1.S1_s_at | 15 | Q1PCR8 | GO:0006118 | Electron Transport |
| Gma.3203.1.S1_at | 15 | No UniProt Hit | GO:0016301 | Kinase Activity |
| GmaAffx.24398.1.A1_at | 15 | Q1SP65 | GO:0005515 | Protein Folding |
| GmaAffx.92612.1.S1_s_at | 15 | Q2PEV8 | GO:0003723 | RNA Binding |
| Gma.7761.1.S1_a_at | 15 | Q9SLZ4 | GO:0003700 | Transcription Factor |
| GmaAffx.18381.1.S1_at | 15 | Q9FI73 | GO:0006810 | Transport |
| GmaAffx.54736.1.S1_at | 15 | No UniProt Hit |  | No UniProt Hit |
| Gma.11156.1.S1_s_at | 15 | Q5JML5 |  | Unknown Function |
| GmaAffx.13040.1.S1_at | 15 | Q6Z1R9 |  | Unknown Function |
| GmaAffx.88272.1.S1_at | 16 | Q9C5K4 | GO:0005524 | ATP binding |
| Gma.4374.1.S1_s_at | 16 | Q2MJ15 | GO:0005506 | Cation transport |
| GmaAffx.16523.1.S1_at | 16 | Q5N8C4 | GO:0016301 | Kinase Activity |
| GmaAffx.77832.1.S1_at | 16 | Q9FX34 | GO:0006499 | Protein Metabolism |
| GmaAffx.1301.47.A1_at | 16 | No UniProt Hit | GO:0003700 | Transcription Factor |
| GmaAffx.11179.2.S1_s_at | 16 | No UniProt Hit |  | No UniProt Hit |
| GmaAffx.15811.1.A1_at | 16 | No UniProt Hit |  | No UniProt Hit |
| GmaAffx.77266.1.S1_at | 16 | No UniProt Hit |  | No UniProt Hit |
| GmaAffx.90082.1.S1_s_at | 16 | No UniProt Hit |  | No UniProt Hit |
| GmaAffx.15192.1.S1_at | 17 | Q1T1I6 | GO:0006812 | Cation transport |
| GmaAffx.89881.1.S1_at | 17 | Q8GUR9 | GO:0006118 | Electron Transport |
| Gma.3138.1.S1_at | 17 | Q9S9W2 | GO:0016491 | Oxidoreductase |
| Gma.10873.1.S1_s_at | 17 | No UniProt Hit |  | No UniProt Hit |
| Gma.17683.1.S1_at | 17 | No UniProt Hit |  | No UniProt Hit |
| Gma.10605.1.S1_at | 17 | Q9LYH6 | GO:0005554 | Unknown Function |
| Gma.5163.2.S1_at | 17 | Q1SBT2 | GO:0005554 | Unknown Function |
| Gma.8881.1.A1_at | 17 | Q9FJL6 | GO:0005554 | Unknown Function |
| GmaAffx.24966.1.S1_at | 17 | Q9SN77 | GO:0005554 | Unknown Function |
| Gma.3137.1.S1_at | 18 | Q1T3D6 | GO:0006812 | Cation Transport |
| GmaAffx.93166.1.S1_s_at | 18 | Q69F96 | GO:0016760 | Cellulose Synthase |
| GmaAffx.31381.1.S1_at | 18 | Q8VYN6 | GO:0006096 | Glycolysis |
| Gma.16673.1.A1_at | 18 | No UniProt Hit |  | No UniProt Hit |
| GmaAffx.87763.1.S1_at | 18 | Q2V398 | GO:0006730 | Protein Metabolism |
| Gma.3510.1.S1_at | 18 | Q39658 | GO:0003700 | Transcription Factor |
| Gma.7768.1.A1_s_at | 18 | Q9SH93 | GO:0005554 | Unknown Function |
| Gma.1955.4.A1_at | 19 | Q5PYQ5 | GO:0005509 | Cation transport |
| GmaAffx.47203.1.S1_at | 19 | Q9FV54 | GO:0005506 | Cation Transport |
| Gma.12704.1.A1_at | 19 | Q2MZW1 | GO:0019825 | Oxidase |
| GmaAffx.75422.1.S1_at | 19 | No UniProt Hit | GO:0006499 | Protein Metabolism |
| Gma.7757.1.S1_at | 19 | Q93XA0 | GO:0003700 | Transcription Factor |
| Gma.5974.1.S1_at | 19 | Q1SCD5 | GO:0005525 | Translation |
| Gma.13798.1.A1_at | 19 | No UniProt Hit |  | No UniProt Hit |
| Gma.602.1.S1_at | 19 | No UniProt Hit |  | No UniProt Hit |
| Gma.9330.1.S1_at | 19 | No UniProt Hit |  | No UniProt Hit |
| GmaAffx.33971.1.A1_at | 19 | No UniProt Hit |  | No UniProt Hit |
| Gma.2120.1.S1_at | 20 | Q330L2 | GO:0015385 | Cation transport |
| Gma.15264.1.S1_at | 20 | Q1SM16 | GO:0003676 | Nucleic Acid Binding |
| GmaAffx.20686.1.A1_at | 20 | No UniProt Hit | GO:0006457 | Protein Folding |
| GmaAffx.89933.1.S1_at | 20 | O65844 | GO:0000163 | Protein Metabolism |
| Gma.12340.1.S1_at | 20 | No UniProt Hit | GO:0005529 | Sugar Binding |
| Gma.13769.1.S1_at | Unknown | No UniProt Hit |  | No UniProt Hit |
| Gma.602.1.S1_at | Unknown | No UniProt Hit |  | No UniProt Hit |
| GmaAffx.32146.1.S1_at | Unknown | Q6QXZ8 | GO:0009775 | Electron Transport |
