# Supplementary material for: Integrating microarray analysis and the soybean genome to understand the soybeans iron deficiency response
Source: BMC Genomics. 2009 Aug 13;10:376. doi: 10.1186/1471-2164-10-376 (PMC2907705; doi:10.1186/1471-2164-10-376)
Supplement: Additional file 7 — Primer sequences used for semi quantitative real time RT-PCR. A table listing the Affymetrix probe IDs and the associated forward and reverse sequences of the primers used in the semi quantitative real time RT-PCR. [file 1471-2164-10-376-S7.doc]

Additional file 7: Primer sequences used for semi quantitative real time RT-PCR to confirm differential expression identified in the microarray.

| Affymetrix Probe Set | Primer Sequences |
| --- | --- |
| Gma.13296.3.S1_at | F: GTGTGATCCAGTATGCATGC |
|  | R: GCAGCAAGGGCTGATGAC |
| Gma.17724.3.S1_at | F: GGTGCAGAGGCACAGCG |
|  | R: CCGTCGAGCTCGGGATC |
| Gma.17825.1.A1_at | F: CTGGTTGGAGAGGACAGAGC |
|  | R: GGTTGAGGGAATCAAGCTCC |
| GmaAffx.89896.1.S1_at | F: CGGTGGTCGAAGGAGC |
|  | R: CCTTTGGAACGGTGACAG |
| GmaAffx.93268.1.S1_at | F: CTTCGGTGGCCGAAGG |
|  | R: CAGCAAAGGATAAGCTCACG |
| GmaAffx.51733.1.A1_at | F: GAAGGGTAAGATGCACTTGACACC |
|  | R: CCTCCAAGGTCTTACAAATGC |
| GmaAffx.88242.1.S1_at | F: CCAAGGGCAAGAAGCCAGC |
|  | R: CATCACAGGCGGTCCACC |
| Gma.16500.1.S1_at | F: GTGTTGTATGTGGACGTTATCTAG |
|  | R: ATCCCAACTGGTGCCATAG |
| Gma.3705.1.S1_at | F: GGATTATTGACTCTCGACAACCG |
|  | R: CGTTCAATCGCATTCAAGC |
| Gma.9609.1.S1_at | F: GAGTTCAGAGCAATGGGCAATGG |
|  | R: GCCAAGTTGCACACAGCCG |
| GmaAffx.36066.1.S1_at | F: GCTCCTCTGCGGATACC |
|  | R: GGACTGACTTTGGCAGC |
